# Ribonucleotide synthesis by NME6 fuels mitochondrial gene expression

**DOI:** 10.15252/embj.2022113256

**Published:** 2023-07-13

**Authors:** Nils Grotehans, Lynn McGarry, Hendrik Nolte, Vanessa Xavier, Moritz Kroker, Álvaro Jesús Narbona‐Pérez, Soni Deshwal, Patrick Giavalisco, Thomas Langer, Thomas MacVicar

**Affiliations:** ^1^ Max Planck Institute for Biology of Ageing Cologne Germany; ^2^ The CRUK Beatson Institute Glasgow UK; ^3^ Cologne Excellence Cluster on Cellular Stress Responses in Aging‐Associated Diseases (CECAD) University of Cologne Cologne Germany

**Keywords:** mitochondria, mitochondrial DNA, mitochondrial transcription, NME6, nucleotide metabolism, Organelles, Translation & Protein Quality

## Abstract

Replication of the mitochondrial genome and expression of the genes it encodes both depend on a sufficient supply of nucleotides to mitochondria. Accordingly, dysregulated nucleotide metabolism not only destabilises the mitochondrial genome, but also affects its transcription. Here, we report that a mitochondrial nucleoside diphosphate kinase, NME6, supplies mitochondria with pyrimidine ribonucleotides that are necessary for the transcription of mitochondrial genes. Loss of NME6 function leads to the depletion of mitochondrial transcripts, as well as destabilisation of the electron transport chain and impaired oxidative phosphorylation. These deficiencies are rescued by an exogenous supply of pyrimidine ribonucleosides. Moreover, NME6 is required for the maintenance of mitochondrial DNA when the access to cytosolic pyrimidine deoxyribonucleotides is limited. Our results therefore reveal an important role for ribonucleotide salvage in mitochondrial gene expression.

## Graphical abstract

A mitochondrial nucleoside diphosphate kinase supplies mitochondria with the pyrimidine ribonucleotides they need for transcription.

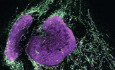

## Introduction

Oxidative phosphorylation (OXPHOS) drives the synthesis of ATP during aerobic respiration and regulates broad cellular functions including redox homeostasis and cell death (Winter *et al*, 2022). The OXPHOS protein complexes at the inner mitochondrial membrane are predominantly composed of nuclear DNA‐encoded subunits that are imported into mitochondria. However, the correct assembly and activity of complexes I, III, IV and V also depend on the integration of subunits encoded by mitochondrial DNA (mtDNA) in the mitochondrial matrix (Fernandez‐Vizarra & Zeviani, 2021). The compact and circular mtDNA encodes 13 OXPHOS subunits, two ribosomal RNAs (rRNAs) and 22 transfer RNAs (tRNAs) in mammalian cells and is maintained at a high, yet variable, copy number across tissues and developmental stages (Filograna *et al*, 2021).

**Figure 1:**
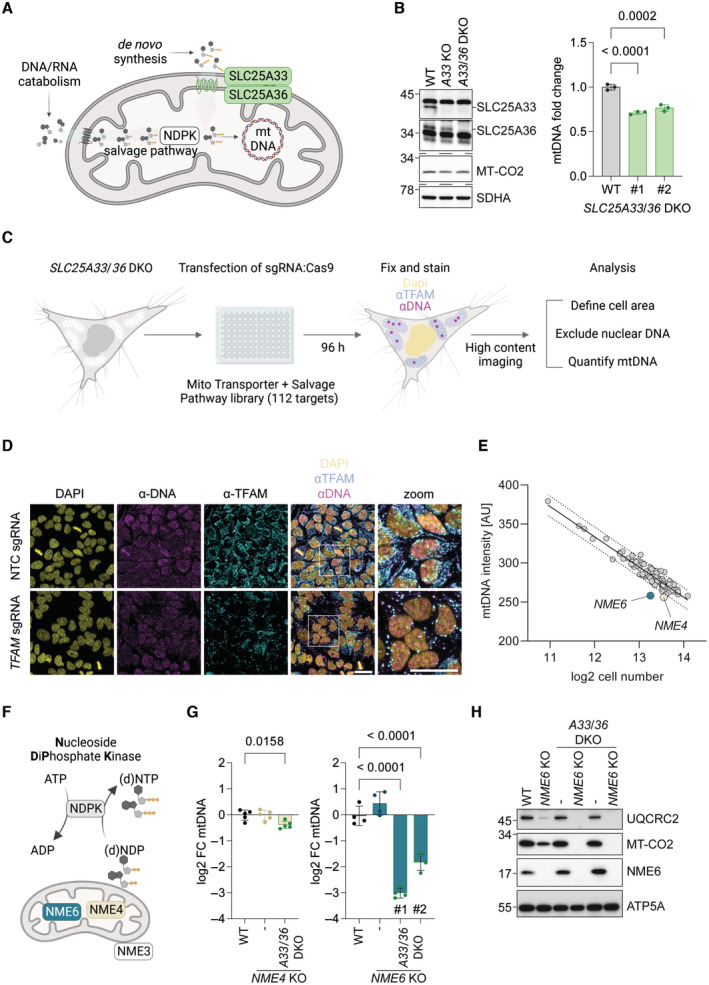
NME6 is required for the maintenance of mtDNA when mitochondrial pyrimidine import is blocked Scheme of the routes by which mitochondria obtain and metabolise pyrimidine nucleotides.Immunoblot analysis of SLC25A33 and SLC25A36 depletion in the indicated knockout (KO) and double knockout (DKO) HeLa cells (left) alongside the relative mtDNA levels of two monoclonal *SLC25A33*/*SLC25A36* DKO cell lines calculated by qPCR (*CYTB/ACTB*; right) (*n* = 3 independent cultures).Experimental flow chart of the arrayed CRISPR‐SpCas9 screen to identify regulators of mtDNA levels in the absence of mitochondrial pyrimidine import.Representative images taken from the arrayed CRISPR‐SpCas9 screen of *SLC25A33*/*SLC25A36* DKO cells showing one field of view from wells transfected with non‐targeting control (NTC) sgRNA or TFAM sgRNA (scale bar = 20 μm).The result of the CRISPR‐SpCas9 screen plotted as the mean mtDNA intensity values against the mean log2 cell number from each sgRNA target. The values for cells transfected with *NME6* sgRNA and *NME4* sgRNA lie below the 95% prediction bands (Least‐squares regression; *R* ^2^ = 0.90; *n* = 3 independent experiments; AU, arbitrary units).Scheme of nucleoside diphosphate kinase (NDPK) enzymatic activity and the reported locations of the mitochondrial NDPKs: NME3, NME4 and NME6.Relative mtDNA levels in *NME4* KO and *NME4*/*SLC25A33*/*SLC25A36* triple KO HeLa (left) or *NME6* KO and two clones of *NME6*/*SLC25A33*/*SLC25A36* triple KO HeLa (right). MtDNA calculated by qPCR (*CYTB/ACTB*) and presented as log2 fold change compared to levels in WT HeLa cells (*n* = 4–5 independent cultures).Representative immunoblot analysis of the indicated HeLa cell lines (UQCRC2, Ubiquinol‐cytochrome C reductase core protein 2; MT‐CO2, mitochondrial‐encoded cytochrome c oxidase II; ATP5A, ATP synthase F1 subunit alpha). Data information: *P*‐values were calculated using one‐way analysis of variance (ANOVA) with Tukey's multiple comparison test (B, G). FC, fold change. Data are means ± standard deviation (SD). Source data are available online for this figure.

The replication of mtDNA and synthesis of mitochondrial RNA (mtRNA) require a constant supply of deoxyribonucleoside triphosphates (dNTPs) and ribonucleoside triphosphates (rNTPs), respectively (Gustafsson *et al*, 2016; D'Souza & Minczuk, 2018). Disturbances in the nucleotide supply lead to mtDNA depletion and/or deletions causing mitochondrial disease (Vafai & Mootha, 2012; Russell *et al*, 2020). Mammalian cells synthesise dNTPs and rNTPs either *de novo* in the cytosol from multiple carbon and nitrogen sources or, in a process termed nucleotide salvage, from (deoxy)ribonucleosides via a series of phosphorylation reactions within the cytosol or within mitochondria (Lane & Fan, 2015). Mitochondria therefore depend on the import of dNTPs and rNTPs or of precursor (deoxy)ribonucleosides across the inner mitochondrial membrane (Mathews & Song, 2007; Wang, 2016) (Fig 1A).

The predominant source and supply route of dNTPs for mtDNA replication is defined by the cell cycle and tissue type (Wang, 2016). Within proliferating cells, mitochondria import *de novo* synthesised dNTPs from the cytosol, while quiescent cells have a greater dependence on mitochondrial nucleotide salvage as a consequence of downregulated cytosolic dNTP synthesis (Ferraro *et al*, 2005; Mathews & Song, 2007). Accordingly, patients with mutations in the mitochondrial pyrimidine salvage pathway enzyme thymidine kinase 2 (*TK2*) show severe depletion of mtDNA in the skeletal muscle (Saada *et al*, 2001; Suomalainen & Isohanni, 2010). Disturbances in mitochondrial nucleotide metabolism also impact cellular nucleotide balance with striking consequences for cellular signalling. Expression of proofreading‐deficient mtDNA polymerase gamma enhances the uptake of mitochondrial dNTPs, which results in the depletion of cytosolic dNTPs and nuclear genomic instability (Hamalainen *et al*, 2019). This was observed in mouse stem cells but not in whole mouse embryos, pointing to cell type specific regulation of mitochondrial dNTP levels (Sharma *et al*, 2020). An enhanced uptake of mitochondrial pyrimidines promotes mtDNA replication, but can also trigger mtDNA release from mitochondria and mtDNA‐dependent inflammatory pathways (Sprenger *et al*, 2021). The mitochondrial dNTP salvage pathway also contributes to innate immune signalling. Stimulation of macrophages induces the expression of the mitochondrial cytidine/uridine monophosphate kinase 2 (CMPK2), which drives the rapid synthesis of mtDNA and supports inflammasome activation (Zhong *et al*, 2018; Ernst *et al*, 2021).

While it has been demonstrated that the mitochondrial dNTP supply can tune mtDNA replication, how mitochondria obtain rNTPs for RNA synthesis, which is required for both transcription and mtDNA replication, remains unclear. By interrogating mitochondrial nucleotide supply pathways, we show here that the nucleoside diphosphate kinase, NME6, is a mitochondrial nucleotide salvage pathway enzyme, which is required for mtRNA synthesis and OXPHOS function, highlighting the critical role of mitochondrial ribonucleotide metabolism in mitochondrial gene expression.

## Results

To address how nucleotide supply regulates mitochondrial gene maintenance and expression, we explored the routes by which mitochondria obtain cytosolic pyrimidine nucleotides for mtDNA and mtRNA synthesis in proliferating cells. We decided to focus on the mitochondrial supply of pyrimidines, since we and others have observed that enhanced mitochondrial import or salvage of pyrimidines is sufficient to increase the abundance of mtDNA (Favre *et al*, 2010; Zhong *et al*, 2018; Sprenger *et al*, 2021). While several mitochondrial solute carriers are known to exchange adenine nucleotides across the inner membrane, only two mitochondrial pyrimidine (deoxy)ribonucleotide carriers (SLC25A33 and SLC25A36) have been identified in mammalian cells (Floyd *et al*, 2007; Di Noia *et al*, 2014), both of which also transport guanine nucleotides *in vitro* (Di Noia *et al*, 2014). Loss of the single homologue of *SLC25A33* and *SLC25A36* in yeast, Rim2, leads to mtDNA depletion and blocks growth on non‐fermentable carbon sources (Van Dyck *et al*, 1995). We generated HeLa cells lacking *SLC25A33* and *SLC25A36* by CRISPR‐SpCas9 mediated genome editing and monitored mtDNA levels. To our surprise, mtDNA levels were only reduced by 20% in these cells (Fig 1B), suggesting that mitochondria can obtain pyrimidine dNTPs via an alternative route when the pyrimidine nucleotide carriers are missing.

### The maintenance of mtDNA depends on NME6 when pyrimidine nucleotide import is blocked

**Figure EV1:**
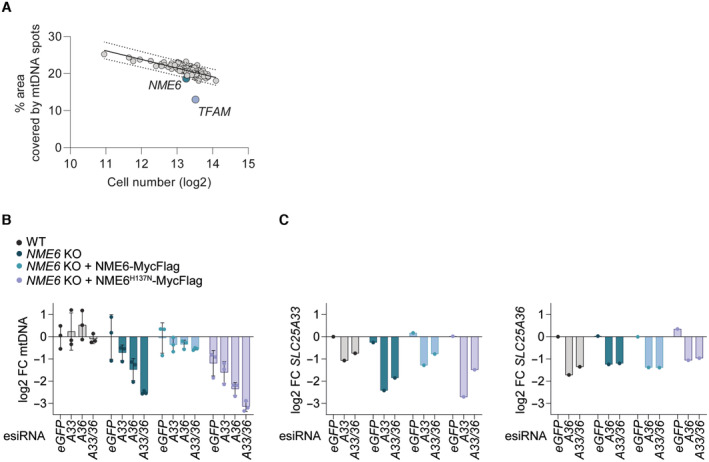
CRISPR‐SpCas9 screen analysis and validation by RNA interference The result of the CRISPR‐SpCas9 screen (Fig 1C) plotted as the percentage of cell cytoplasm area occupied by mtDNA spots against the mean log2 cell number from each sgRNA target. The values for cells transfected with *NME6* sgRNA and *TFAM* sgRNA lie below the 95% prediction bands (least‐squares regression; *R* ^2^ = 0.52; *n* = 3 independent experiments).MtDNA level monitored by qPCR (*CYTB/ACTB*) in the indicated HeLa cell lines treated with esiRNA targeting *GFP* (control), *SLC25A33* (A33), *SLC25A36* (A36) or *SLC25A33* + *SLC25A36* (A33/A36) relative to WT cells (log2; *n* = 3 independent cultures).
*SLC25A33* (left) and *SLC25A36* (right) transcript levels in samples from a representative experiment in (B) monitored by qRT‐PCR (log2; *n* = 1). Data information: FC, fold change. Data are means ± SD.

To identify genes that regulate mtDNA content in cells lacking the canonical mitochondrial pyrimidine transporters SLC25A33 and SLC25A36, we performed an arrayed CRISPR‐SpCas9 knockout high‐content microscopy screen. HeLa cells lacking both pyrimidine nucleotide carriers were transfected with SpCas9 nuclease and an arrayed CRISPR library, wherein each well contained three sgRNAs targeting individual genes with proposed roles in mitochondrial metabolite transport or pyrimidine nucleotide salvage (112 genes) (Dataset EV1). We visualised mtDNA 96 h following transfection by immunofluorescence with an anti‐DNA antibody and quantified the mean fluorescence intensity per cell after exclusion of the nuclear DNA signal (Fig 1C and D). The mtDNA level inversely correlated with cell confluency in each well, which led us to plot the mean fluorescence intensity of mtDNA against cell number to identify potential outliers (Fig 1E). Two outliers corresponded to sgRNA targeting the mitochondrial nucleoside diphosphate kinases (NDPK), *NME4* and *NME6*, which suggested that their loss renders cells unable to maintain mtDNA levels when pyrimidine nucleotide import is blocked (Fig 1E). The library also included sgRNA targeting mitochondrial transcription factor A (TFAM), a mtDNA‐binding protein that is essential for the packaging of mtDNA into compact nucleoids (Garrido *et al*, 2003; Legros *et al*, 2004). Although TFAM protein level correlates closely with the abundance of mtDNA (Larsson *et al*, 1998; Ekstrand *et al*, 2004; Kanki *et al*, 2004; Bonekamp *et al*, 2021) and TFAM was selectively and efficiently depleted in our screen (Fig 1D), *TFAM* sgRNA did not register as an outlier in our mtDNA fluorescence intensity analysis (Fig 1E). It should be noted, however, that the residual TFAM condensed with mtDNA in enlarged nucleoids in line with previous observations in cells transiently depleted of TFAM (West *et al*, 2015; Feric *et al*, 2022). We calculated the area of mtDNA puncta as an additional readout of mtDNA homeostasis and found that, analogous to the mean fluorescence intensity, the total area of mtDNA also declined with cell confluency (Fig EV1A). The total area of mtDNA puncta per cell was diminished in cells transfected with *TFAM* sgRNA and reduced modestly in cells transfected with *NME6* sgRNA (Fig EV1A).

The non‐metastatic (NME) gene family of NDPKs generate (d)NTPs by transferring the terminal phosphate group predominantly from ATP to dNDPs or rNDPs via a transient phospho‐histidine intermediate (Fig 1F) (Boissan *et al*, 2018). Three NME family members are reported to reside at mitochondria in different subcompartments; NME3 is located at the mitochondrial surface (Chen *et al*, 2019), NME4 has been detected in both the intermembrane space and matrix (Milon *et al*, 2000; Tokarska‐Schlattner *et al*, 2008) and NME6 is present in the mitochondrial matrix (Proust *et al*, 2021). The results from our CRISPR screen indicated that NME4 and NME6 maintain mtDNA in the absence of pyrimidine nucleotide transport. We therefore deleted *NME4* and *NME6* by CRISPR/Cas9‐mediated genome editing in WT cells and in cells lacking SLC25A33 and SLC25A36 and determined mtDNA levels by real‐time quantitative PCR (qPCR). Loss of NME4 or NME6 did not alter mtDNA levels (Fig 1G). However, the combined knockout of *NME6*, *SLC25A33* and *SLC25A36* caused a dramatic loss of mtDNA to 15–25% of WT levels (Fig 1G) and resulted in the loss of the mtDNA‐encoded protein cytochrome c oxidase II (MT‐CO2; Fig 1H). In contrast, deletion of *NME4* did not affect the accumulation of mtDNA in the absence of SLC25A33 and SLC25A36 (Fig 1G). We therefore conclude that NME6 but not NME4 is required for pyrimidine nucleotide salvage in these cells. To further validate our findings, we depleted SLC25A33 and SLC25A36 individually and together in WT and *NME6* knockout cells using short interfering RNA (esiRNA). Knockdown of the pyrimidine carriers significantly depleted mtDNA in cells lacking NME6 but not in WT cells (Fig EV1B and C). NME6 is ubiquitously expressed in humans and catalyses phosphotransfer through a conserved histidine residue within an NDPK consensus motif at position 137 (H137) (Tsuiki *et al*, 1999). Importantly, mtDNA levels were maintained in *NME6* knockout cells expressing WT NME6‐MycFlag but not in cells expressing kinase inactive mutant NME6 (NME6^H137N^‐MycFlag) (Fig EV1B). Collectively, these data demonstrate that NME6 maintains the mitochondrial genome, if the supply of pyrimidine nucleotides from the cytosol is limited, and indicate that NME6 generates dNTPs within mitochondria (Fig 1A).

### NME6 supports cell proliferation independent of mtDNA synthesis

**Figure 2:**
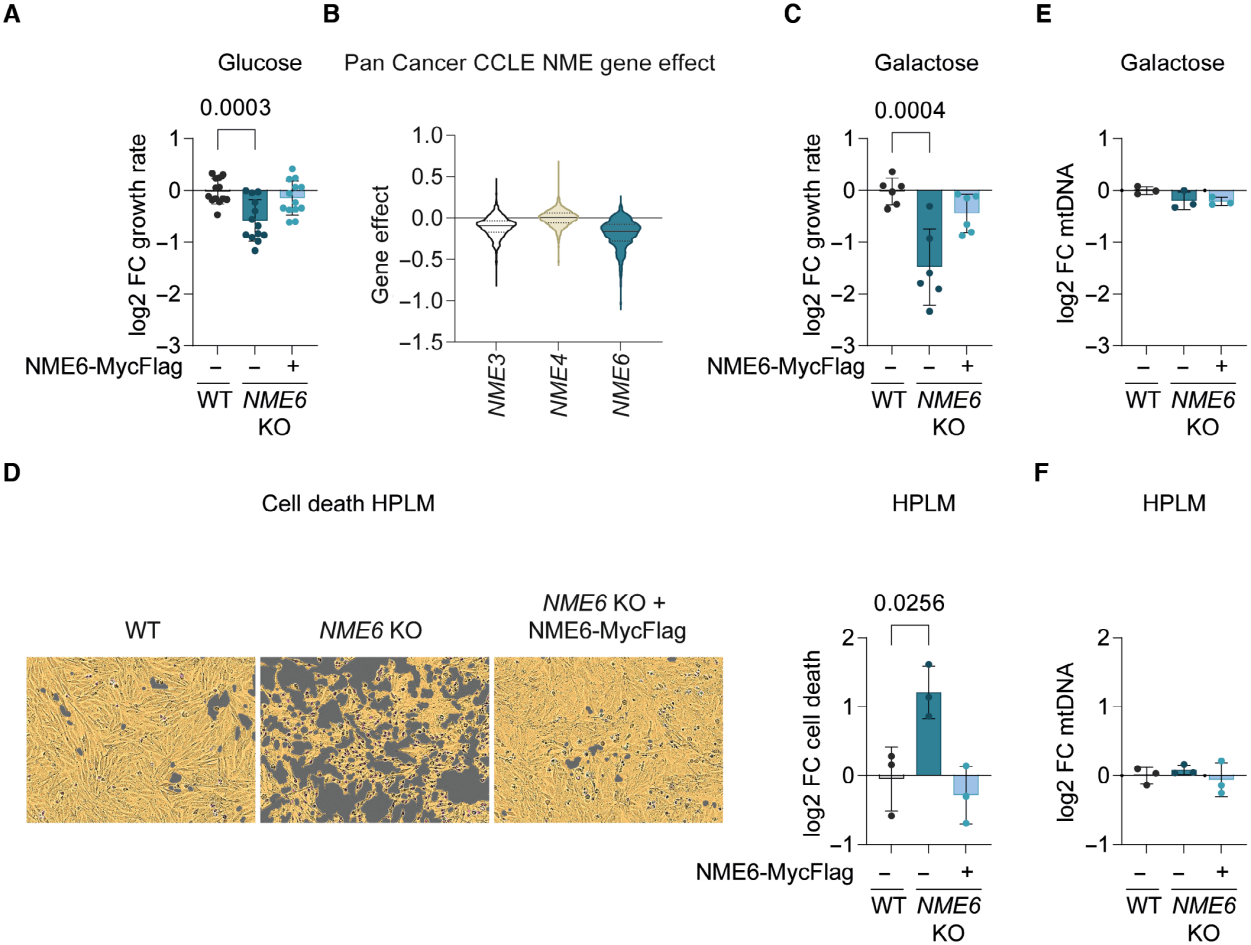
NME6 supports cell proliferation independent of mtDNA synthesis The growth rate of *NME6* KO and *NME6* KO + NME6‐MycFlag HeLa relative to WT HeLa cells incubated in DMEM containing 25 mM glucose (log2; *n* = 13 independent cultures).Violin plot of gene effects of *NME3*, *NME4* or *NME6* depletion in 1,086 cell lines from the Cancer Cell Line Encyclopaedia (CCLE) determined by CRISPR screening (DepMap 22Q2 Public+Score, Chronos; solid line denotes median, dotted line denotes 25% quartile).The growth rate of *NME6* KO and *NME6* KO + NME6‐MycFlag HeLa relative to WT HeLa cells incubated in DMEM containing 10 mM galactose (log2; *n* = 6 independent cultures).Representative live‐cell images of the indicated cell lines grown in Human Plasma‐Like Medium (HPLM) (left) and calculated cell death after 96 h (right). Cell confluency is depicted with the yellow mask and dead cells are identified by SYTOX green staining in purple (*n* = 3 independent cultures).MtDNA level monitored by qPCR (*CYTB/ACTB*) in *NME6* KO and *NME6* KO + NME6‐MycFlag HeLa relative to WT HeLa cells in DMEM containing 10 mM galactose (*n* = 3 independent cultures).MtDNA level monitored by qPCR (*CYTB/ACTB*) in *NME6* KO and *NME6* KO + NME6‐MycFlag HeLa relative to WT HeLa cells in HPLM (*n* = 3 independent cultures). Data information: *P*‐values were calculated using one‐way ANOVA with Tukey's multiple comparison test (A, C–F). FC, fold change. Data (except B) are means ± SD. Source data are available online for this figure.

**Figure EV2:**
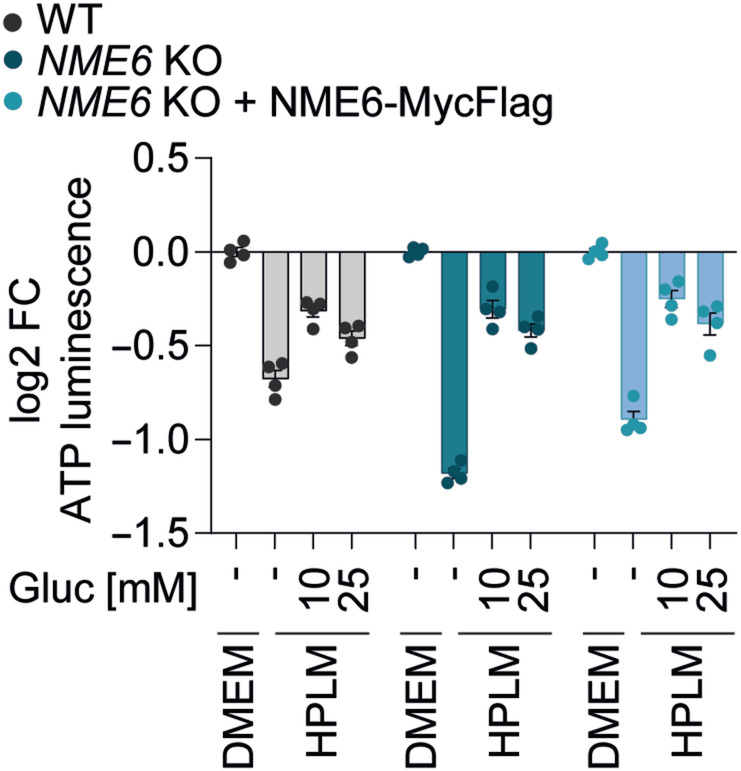
Glucose is limiting for growth in HPLM Cell viability in WT, *NME6* KO and *NME6* KO + NME6 MycFlag HeLa cells incubated in HPLM supplemented with different concentrations of glucose (standard HPLM contains 5 mM glucose). Cell viability was determined by ATP luminescence assay and analysed relative to DMEM (log 2; *n* = 4 independent cultures). FC, fold change. Data are means ± SD.

Although the loss of NME6 did not affect mtDNA levels in WT cells, we observed reduced growth of NME6‐deficient cells on glucose medium (Fig 2A). This is consistent with the fitness dependency of many cancer cell lines on *NME6* revealed by a DepMap analysis of CRISPR knockout screens (Fig 2B). The growth defect of *NME6* knockout cells was more severe in galactose medium, when cell growth increasingly depends on glutaminolysis and OXPHOS (Reitzer *et al*, 1979; Rossignol *et al*, 2004) (Fig 2C). *NME6* was recently identified in a CRISPR‐SpCas9 screen as essential for survival in human plasma like medium (HPLM) (Rossiter *et al*, 2021) and, consistently, we observed that NME6 depleted HeLa cells could not survive in HPLM for extended periods (Fig 2D). This was likely due to glucose exhaustion in the HPLM of *NME6* knockout HeLa cells since glucose supplementation could restore the viability of NME6‐depleted cells in HPLM (Fig EV2). The increased dependency on NME6 in galactose or HPLM medium was independent of mtDNA levels, which remained normal in cells lacking NME6 regardless of the growth medium (Fig 2E and F). These growth assays thus demonstrate that proliferating cells require NME6, even when the mitochondrial import of dNTPs is normal and the maintenance of mtDNA does not depend on NME6.

### Mitochondrial respiration and OXPHOS subunit homeostasis depend on NME6

**Figure 3:**
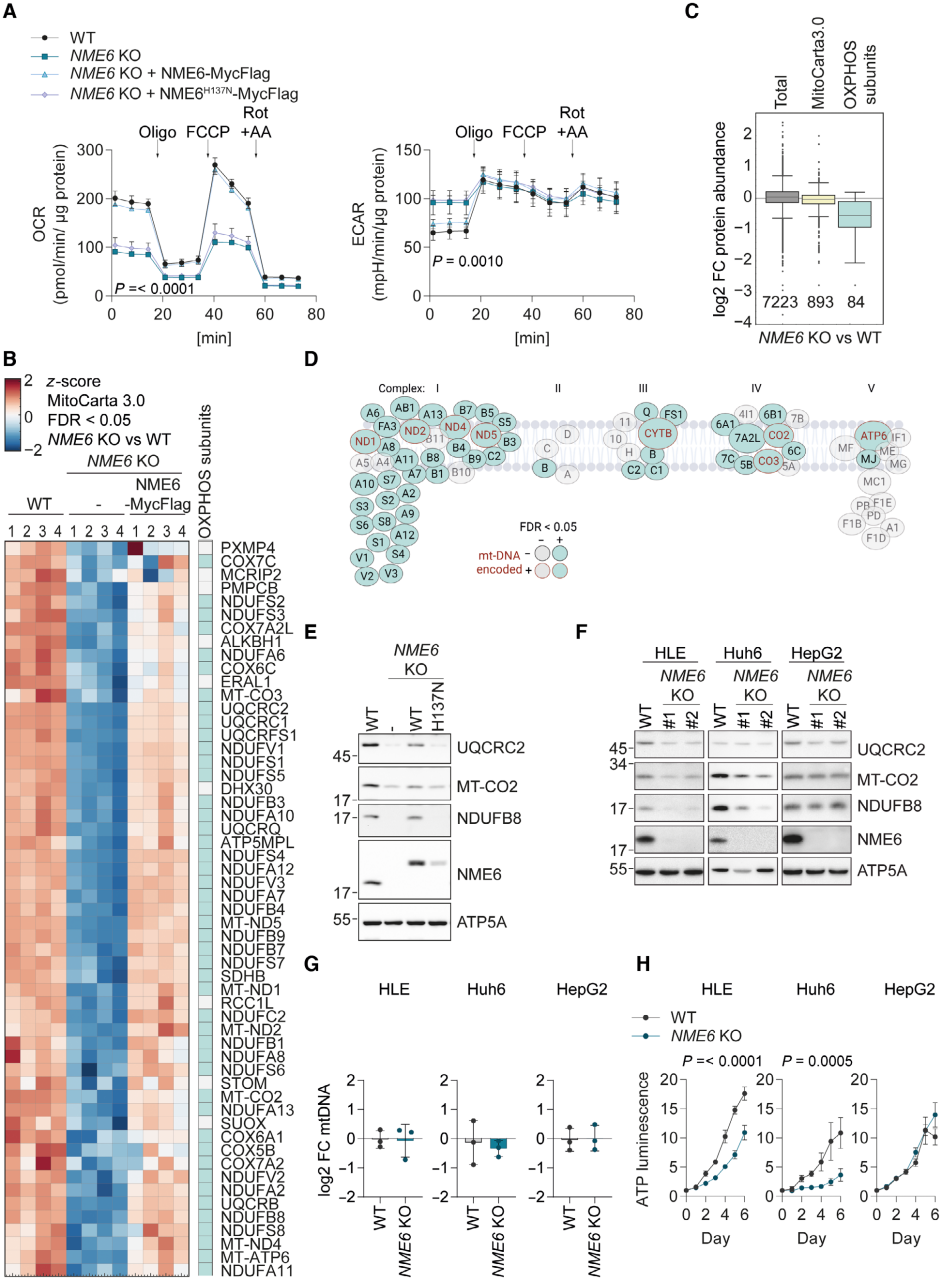
Mitochondrial respiration and OXPHOS depend on NME6 Oxygen consumption rates (OCR) and extracellular acidification rates (ECAR) of the indicated HeLa cell lines during a mitochondrial stress test with inhibitor treatments at the indicated timepoints. *P*‐values for OCR: two‐way ANOVA *P*‐value (time) = < 0.0001, *P*‐value (genotype) = < 0.0001, *P*‐value (interaction) = < 0.0001; *P*‐values for ECAR:two‐way ANOVA *P*‐value (time) = < 0.0001, *P*‐value (genotype) = < 0.001, *P*‐value (interaction) = 0.0517; *P*‐values for genotype are shown (Oligo, oligomycin; FCCP, carbonyl cyanide‐p‐trifluoromethoxyphenylhydrazone; Rot + AA, rotenone and antimycin A; *n* = 3 independent experiments).Heat map representation of *z*‐scores of log2‐transformed protein intensities determined by quantitative mass spectrometry and filtered for mitochondrial proteins according to MitoCarta 3.0 (Rath *et al*, 2021) (cluster four of Fig EV3A). The proteins shown clustered together due to their significant (permutation‐based FDR < 0.05, *s*_0_ = 0.1) depletion in *NME6* KO HeLa cells compared to WT and *NME6* KO + NME6‐MycFlag HeLa cells. OXPHOS subunits are indicated in the right column (*n* = 4 independent cultures).Box plot analysis of log2 fold change in protein intensities in *NME6* KO compared to WT HeLa cells. Distribution of the complete set of quantified protein groups: Total (7,223 proteins detected), MitoCarta 3.0 positive (893 proteins detected) and OXPHOS subunits (CI – CV of the MitoCarta 3.0 pathway annotations; 84 proteins detected). Box limits denote 25‐ and 75% quartile, line denotes the median, whiskers denote 1.5 × interquartile range deviation from the median.Graphical representation of all respiratory complex subunit proteins detected in our proteomic assay to highlight the respiratory complexes most affected by loss of NME6 as also shown in B. OXPHOS subunits depleted in *NME6* KO cells compared to WT and *NME6* KO + NME6‐MycFlag HeLa cells are in teal. MtDNA‐encoded OXPHOS subunits are labelled in red. Subunits that were not significantly altered between genotypes are in grey.Immunoblot analysis of WT HeLa cells, *NME6* KO cells and *NME6* KO cells expressing NME6‐MycFlag (WT) or NME6^H137N^‐MycFlag (H137N).Immunoblot analysis of WT cells and two *NME6* KO clones (#1, #2) generated in three different liver cancer cell lines: HLE, Huh6 and HepG2.MtDNA level monitored by qPCR (*CYTB/ACTB*) in *NME6* KO relative WT cells in the indicated liver cancer cell lines (*n* = 3 independent cultures).The relative growth of WT and *NME6* KO liver cancer cell lines monitored on each day (d) using an ATP luminescence assay. *P*‐values for HLE cells: two‐way ANOVA *P*‐value (time) = < 0.0001, *P*‐value (genotype) = < 0.0001, *P*‐value (interaction) = < 0.0001; *P*‐values for Huh6 cells: two‐way ANOVA *P*‐value (time) = < 0.0001, *P*‐value (genotype) = 0.0005, *P*‐value (interaction) = < 0.0001; *P*‐values for genotype are shown (*n* = 4 independent cultures). Data information: FC, fold change. Data (except C) are means ± SD. Source data are available online for this figure.

Consistent with the impaired growth in galactose medium (Fig 2C), the loss of NME6 strongly reduced cellular oxygen consumption rates (OCR) and resulted in a concomitant increase in the extracellular acidification rate (ECAR) (Fig 3A). Enhanced ECAR is indicative of upregulated glycolysis, which likely explains the greater glucose dependency in cells lacking NME6 (Fig EV2). Normal OCR and ECAR were restored in *NME6* knockout cells upon the expression of WT NME6‐MycFlag but not NME6^H137N^‐MycFlag, demonstrating that mitochondrial function requires NME6 kinase activity (Fig 3A).

**Figure EV3:**
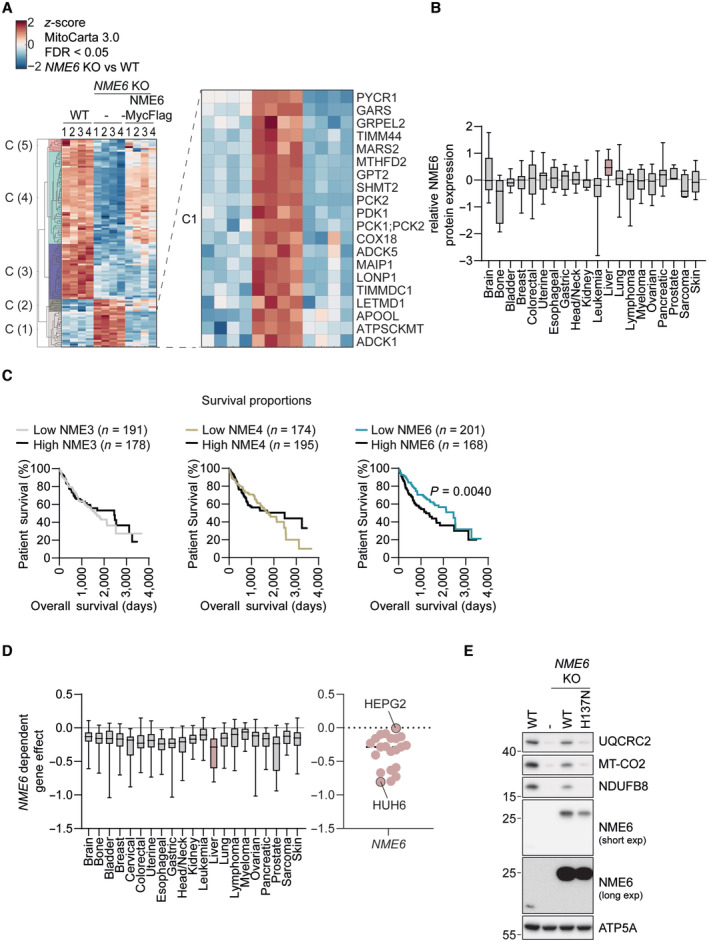
Alterations to the proteome upon NME6 depletion and further characterisation of NME6 in liver cancer Extended unsupervised hierarchical row clustering (Euclidean distance, complete method) representation of significantly different proteins (FDR < 0.05) (*NME6* KO vs. WT) *z*‐scores of log2‐transformed protein intensities determined by quantitative mass spectrometry and filtered for mitochondrial proteins according to MitoCarta 3.0 (Rath *et al*, 2021). The cluster presented in Fig 3B is visible at the top (C4). The bottom cluster (C1) is expanded here to reveal proteins that are significantly upregulated in *NME6* KO cells compared to WT and *NME6* KO + NME6‐MycFlag HeLa cells (*n* = 4 independent cultures).Relative NME6 protein expression in cell lines across the indicated cancer types determined by quantitative proteomic profiling (Nusinow *et al*, 2020). Data obtained from depmap.org/portal (solid line = median, box limits = 25^th^ and 75^th^ percentile and whiskers = maxima and minima).Kaplan–Meier plots showing patient survival in the indicated liver hepatocellular carcinoma cohorts (LIHC) from The Cancer Genome Atlas Program (TCGA) analysed using http://www.tcga‐survival.com. *P*‐values were determined using a log rank test (Smith & Sheltzer, 2022).Stratification of the gene effect of NME6 depletion from Fig 2A into cancer type (left, solid line = median, box limits = 25^th^ and 75^th^ percentile and whiskers = maxima and minima). Individual cell line *NME6* gene effects are also shown with HepG2 and Huh6 cell lines highlighted (right, note that HLE cells are not included in DepMap 22Q2 Public+Score, Chronos).Immunoblot analysis of WT HLE cells, *NME6* KO cells and *NME6* KO cells expressing NME6‐MycFlag (WT) or NME6^H137N^‐MycFlag (H137N).

To explore why mitochondrial respiration depends on NME6, we examined mitochondrial protein homeostasis by quantitative proteomics and analysed mitochondrial proteins (MitoCarta 3.0) (Rath *et al*, 2021) that were significantly altered in an NME6‐dependent manner by unsupervised hierarchical clustering (Fig EV3A). While 20 mitochondrial proteins accumulated in *NME6* knockout cells (Fig EV3A), the largest cluster consisted of 55 mitochondrial proteins that were significantly depleted in cells lacking NME6 compared to WT and NME6‐MycFlag complemented cells (Fig 3B; Dataset EV2). Remarkably, 47 of the 55 mitochondrial proteins were OXPHOS subunits, resulting in the collective depletion of OXPHOS proteins relative to other mitochondrial proteins, which was also revealed by unbiased 1D‐enrichment driven pathway analysis (Fig 3C and D). These data demonstrate that OXPHOS subunit homeostasis depends on NME6, while overall mitochondrial mass is unaffected by NME6 loss (Fig 3C). Immunoblotting of selected OXPHOS subunits revealed that the abundance of OXPHOS subunits in *NME6* knockout cells was restored upon expression of NME6 but not in the presence of the kinase dead variant NME6^H137N^ (Fig 3E). Thus, respiration depends on enzymatically active NME6 (Fig 3A).

We next expanded our analysis to liver cancer cell lines, since the expression of NME6 is increased in these cell lines relative to other cancer types (Fig EV3B). The expression of NME6 correlates with an unfavourable prognosis in liver cancer patients (Fig EV3C). OXPHOS subunits were diminished in two out of three *NME6* knockout human liver cancer cell lines (Fig 3F), while the abundance of mtDNA was not affected (Fig 3G). The absence of NME6 in HLE and Huh6 cells, but not HepG2 cells, also resulted in a significant growth defect (Fig 3H), which was consistent with publicly available CRISPR screening data (Fig EV3D; depmap.org/portal) and correlated with the levels of OXPHOS subunits in these cell lines (Fig 3F). Finally, complementation of *NME6* knockout HLE cells with WT NME6‐MycFlag but not NME6^H137N^‐MycFlag restored OXPHOS homeostasis (Fig EV3E), thus confirming the requirement for NME6 kinase activity in these cells. Collectively, we conclude that NME6 is required for respiration and the maintenance of OXPHOS subunits independent of its role in mtDNA synthesis.

### Mitochondrial gene expression depends on NME6

**Figure 4:**
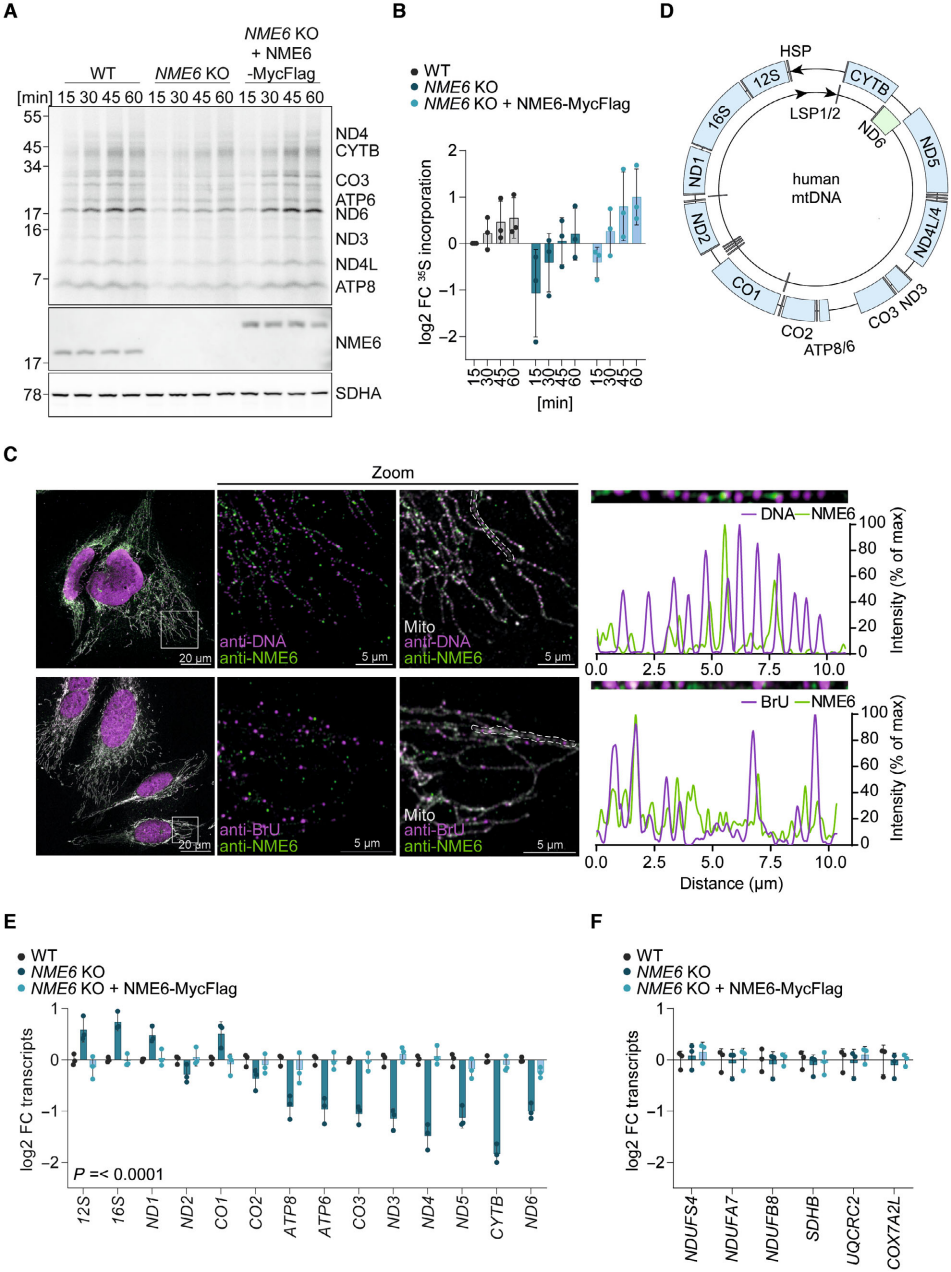
NME6 regulates mitochondrial gene expression Representative mitochondrial translation assay monitored by the incorporation of ^35^S methionine and cysteine into the indicated mtDNA‐encoded proteins followed by autoradiography (top panel). MtDNA encoded proteins are labelled according to expected size and NME6 and SDHA immunoblots are shown below.Quantification of ^35^S methionine and cysteine incorporation into all mtDNA‐encoded proteins labelled in (B) relative to WT HeLa cells at 15 min (log2; mitochondrial preparations from *n* = 3 independent cultures).Immunofluorescence of NME6 with mtDNA (top) or bromouridine (BrU) labelled nascent mtRNA (bottom) in HeLa cells imaged by confocal microscopy. Relative fluorescence intensities were calculated from linescans generated within the mitochondrial regions indicated by dotted lines.Scheme of human mtDNA organisation with the heavy strand promoter (HSP) represented by an arrow on the outside and the light strand promoters (LSP) by two arrows on the inside. Heavy strand ‐transcripts are labelled in blue, light strand‐transcript is labelled in green, tRNAs are labelled in grey.MtDNA‐encoded transcript levels analysed by qRT‐PCR in *NME6* KO and *NME6* KO + NME6‐MycFlag cells relative to WT HeLa cells. *P*‐values for mtDNA‐encoded transcripts: two‐way ANOVA *P*‐value (transcript) = < 0.0001, *P*‐value (genotype) = < 0.0001, *P*‐value (interaction) = < 0.0001 (log2; *n* = 3 independent cultures).Nuclear DNA‐encoded transcript levels analysed by qRT‐PCR in *NME6* KO and *NME6* KO + NME6‐MycFlag HeLa cells relative to WT (log2; *n* = 3 independent cultures). Data information: FC, fold change. Data are means ± SD. Source data are available online for this figure.

**Figure EV4:**
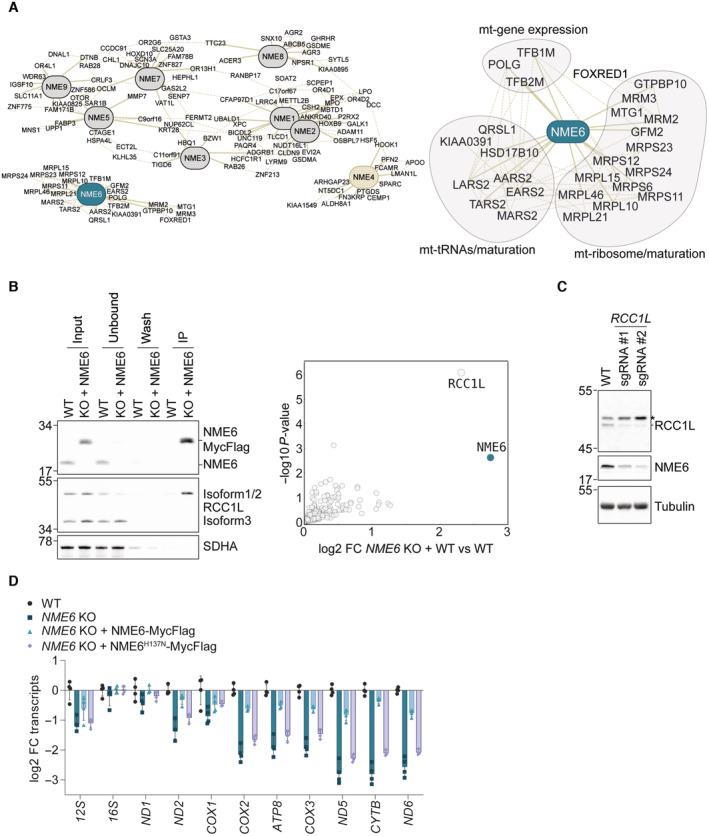
Proteomic analysis of immunoprecipitates shows interaction between NME6 and RCC1L Pan‐cancer gene coessentiality network visualisation of the top 15 positively correlated genes (solid lines) and five secondary node positive interactions (dotted lines) with all NME family members (*NME1‐9*) using FIREWORKS (Amici *et al*, 2021) (left) and *NME6* correlated genes were grouped further into manually annotated mitochondrial functions (right).Representative immunoblot (left) and proteomic analysis (right) following immunoprecipitation of NME6‐MycFlag from HeLa cell mitochondrial lysates using a Flag antibody (*n* = 4 independent experiments). FC, fold change.Immunoblot analysis of HeLa WT cells and two polyclonal *RCC1L* KO populations.MtDNA‐encoded transcript levels analysed by qRT‐PCR in *NME6* KO, *NME6* KO + NME6‐MycFlag and *NME6* KO H137N‐MycFlag cells relative to WT HLE cells (log2; *n* = 4 independent cultures).

The depletion of mtDNA‐encoded proteins within complex I, III, IV and V (Fig 3D) in *NME6* knockout cells prompted us to monitor mitochondrial protein synthesis *in vitro*. We observed reduced synthesis of mtDNA‐encoded subunits in mitochondria lacking NME6 (Fig 4A and B), indicating defective mitochondrial transcription or translation in the absence of NME6. Consistently, gene coessentiality network analysis across hundreds of heterogenous cancer cell lines using the FIREWORKS (Fitness Interaction Ranked nEtWORKS) web tool (Amici *et al*, 2021) revealed that the top coessential genetic interactors with *NME6* are regulators of mtDNA replication, transcription, mitochondrial tRNA maturation and mitochondrial ribosome (mitoribosome) biogenesis, which is unique within the *NME* gene family (Fig EV4A).

Immunoprecipitation of NME6‐MycFlag coupled with mass spectrometry identified the putative mitoribosome assembly factor, RCC1L, to be the only high confidence interaction partner of NME6 (Fig EV4B; Dataset EV3). This is in line with previous proximity labelling and immunoprecipitation assays (Floyd *et al*, 2016; Antonicka *et al*, 2020; Proust *et al*, 2021). The depletion of RCC1L by CRISPR‐Cas9 resulted in a concomitant depletion of NME6 (Fig EV4C). RCC1L‐FLAG colocalises with mtRNA granules (Antonicka *et al*, 2017) and our confocal fluorescence imaging revealed that endogenous NME6 forms puncta that overlap partially with mtDNA nucleoids and mtRNA granules (Fig 4C). However, unlike cells depleted of RCC1L (Reyes *et al*, 2020), we did not observe any disruption of mitoribosome proteins in cells lacking NME6 (Figs 3B and EV3A), which argues against NME6 being required for mitoribosome assembly.

Mitochondrial transcription is initiated from a single promoter on the heavy‐strand and two promoters on the light‐strand of mtDNA to yield polycistronic transcripts that are further processed to individual mitochondrial messenger (m)RNAs, transfer (t)RNAs and ribosomal (r)RNAs (Miranda *et al*, 2022; Tan *et al*, 2022) (Fig 4D). We measured the levels of mitochondrial messenger RNA (mRNA) and ribosomal RNA (rRNA) by qPCR and observed a striking pattern of mitochondrial mRNA depletion in cells lacking NME6 that correlated with the distance from the heavy strand promoter (Fig 4D and E). Heavy‐strand mRNAs from *ATP8* onwards, as well as *ND6* on the light‐strand, were significantly lower in the absence of NME6 compared to WT and NME6‐MycFlag complemented cells (Fig 4E). Mitochondrial transcripts were also depleted in *NME6* knockout HLE cells and the fold change reduction of heavy‐strand transcripts was greatest for those furthest from the promoter (Fig EV4D). The transcript levels of nuclear DNA encoded OXPHOS subunits were unaffected by the loss of NME6 (Fig 4F) despite their reduced protein levels (Fig 3D). Collectively, these data highlight a crucial role for NME6 in the maintenance of mitochondrial transcripts, which could explain the OXPHOS deficiency in cells lacking NME6.

### NME6 supplies rNTPs for mitochondrial transcription

**Figure 5:**
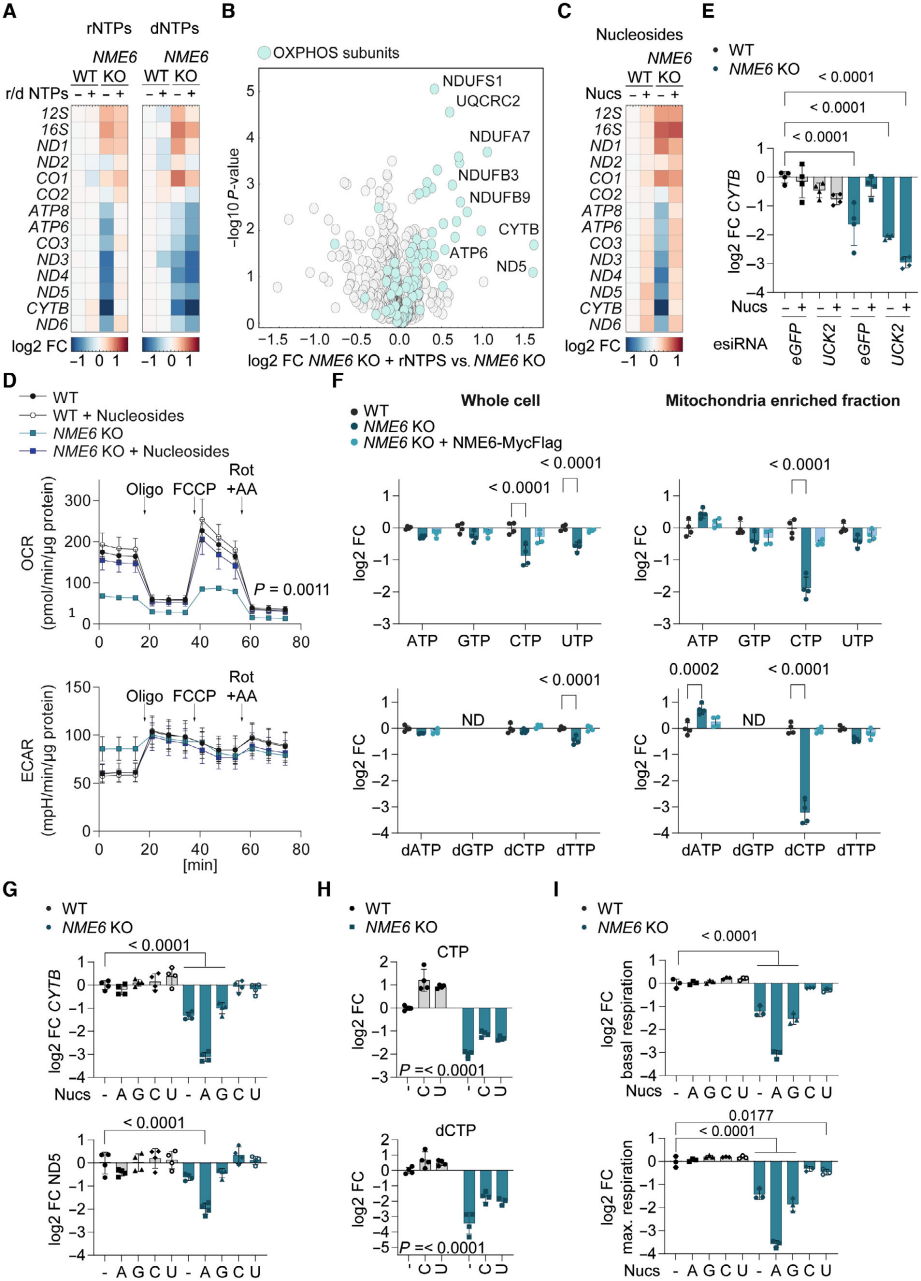
NME6 maintains pyrimidine ribonucleotide triphosphates for mitochondrial transcription Heat map of log2 transformed mean mitochondrial transcript levels of WT and *NME6* KO HeLa cells incubated with 100 μM rNTPs or dNTPs for 48 h relative to untreated WT cells analysed by qRT‐PCR (*n* = 3 independent cultures).Volcano plot representation of log2 fold change in proteins between *NME6* KO cells treated with 100 μM rNTPs for 120 h and untreated *NME6* KO HeLa cells determined by quantitative mass spectrometry. OXPHOS subunits are highlighted in teal (*n* = 3 independent cultures).Heat map of log2 transformed mean mitochondrial transcript levels of WT and *NME6* KO HeLa cells incubated with 100 μM nucleosides for 48 h relative to untreated WT cells analysed by qRT‐PCR (*n* = 3 independent cultures).Oxygen consumption rates (OCR) and extracellular acidification rates (ECAR) of WT and *NME6* KO HeLa cells incubated with or without nucleosides (100 μM) for a minimum of 120 h. Mitochondrial stress test was performed as in Fig 3A (*n* = 3 independent experiments). *P*‐values for OCR: two‐way ANOVA *P*‐value (time) = < 0.0001, *P*‐value (genotype) = 0.0011, *P*‐value (interaction) = < 0.0001; *P*‐values for ECAR: two‐way ANOVA *P*‐value (time) = < 0.0001, *P*‐value (genotype) = 0.8994, *P*‐value (interaction) = < 0.0001.
*CYTB* transcript levels analysed by qRT‐PCR in WT and *NME6* KO HeLa cells transfected with the indicated esiRNA and incubated with or without nucleosides (100 μM) for 72 h (log2; *n* = 4 independent cultures). *P*‐values were calculated using a one‐way ANOVA.Nucleotide levels in whole cell (left) and mitochondria enriched fractions (right) from *NME6* KO and *NME6* KO + NME6‐MycFlag (WT) cells compared to WT HeLa cells as determined by quantitative mass spectrometry. The NTPs (top) and dNTPs (bottom) are shown except for dGTP which was not detected in our analysis. *P*‐values for whole cell rNTPs: two‐way ANOVA *P*‐value (rNTP) = 0.0018, *P*‐value (genotype) = < 0.0001, *P*‐value (interaction) = 0.0027; *P*‐values for mito enriched fraction rNTPs: two‐way ANOVA *P*‐value (rNTP) = < 0.0001, *P*‐value (genotype) = < 0.0001, *P*‐value (interaction) = < 0.0001; *P*‐values for whole cell dNTPs: two‐way ANOVA *P*‐value (dNTP) = 0.0096, *P*‐value (genotype) = < 0.0001, *P*‐value (interaction) = 0.0027; *P*‐values for mito enriched fraction dNTPs: two‐way ANOVA *P*‐value (dNTP) = < 0.0001, *P*‐value (genotype) = < 0.0001, *P*‐value (interaction) = < 0.0001; Significant multiple comparison *P*‐values are shown (log2; *n* = 4 independent cultures).
*CYTB* (top) and *ND5* (bottom) transcript levels analysed by qRT‐PCR in WT and *NME6* KO HeLa cells incubated with the indicated nucleoside species for 48 h. *P*‐values were calculated using a one‐way ANOVA (A, adenosine; G, guanosine; C, cytidine; U, uridine; log2; 100 μM; *n* = 4 independent cultures).CTP (top) and dCTP (bottom) levels in the mitochondria enriched fraction of WT and *NME6* KO HeLa cells incubated with the indicated nucleoside species for 120 h as determined by quantitative mass spectrometry. *P*‐values for CTP: two‐way ANOVA *P*‐value (supplementation) = < 0.0001, *P*‐value (genotype) = < 0.0001, *P*‐value (interaction) = 0.3179; *P*‐values for dCTP: two‐way ANOVA *P*‐value (supplementation) = < 0.0001, *P*‐value (genotype) = < 0.0001, *P*‐value (interaction) = 0.0569; *P*‐values for supplementation are shown (C, cytidine; U, uridine; log2; 100 μM; *n* = 4 independent cultures).Basal (top) and maximal (bottom) oxygen consumption rates of WT and *NME6* KO HeLa cells incubated with the indicated nucleoside species for a minimum of 120 h relative to untreated WT cells (A, adenosine; G, guanosine; C, cytidine; U, uridine; log2; 100 μM; *n* = 4 independent experiments). Basal and maximal rates were calculated from Fig EV5G. *P*‐values were calculated using a one‐way ANOVA. Data information: FC, fold change. Data are means ± SD. Source data are available online for this figure.

The synthesis of the almost genome‐length polycistronic transcripts by POLRMT requires an adequate supply of rNTPs. To test whether NME6 supplies rNTPs for mitochondrial transcription, we supplemented *NME6* knockout cells with rNTPs or dNTPs and measured mitochondrial transcript levels by qPCR. Strikingly, mitochondrial mRNAs were restored to WT levels in *NME6* knockout cells treated with rNTPs, while supplementation with dNTPs had no effect (Fig 5A). Proteomic analysis confirmed that supplementation with rNTPs, was sufficient to increase the levels of OXPHOS subunits in *NME6* knockout cells (Fig 5B; Dataset EV4). Exogenous rNTPs are likely hydrolysed by ectonucleotidases prior to cell uptake as ribonucleosides (Zimmermann, 1999; Pastor‐Anglada & Perez‐Torras, 2018). We therefore treated cells with a mix of nucleosides that included the four ribonucleosides cytidine, uridine, guanosine and adenosine and the deoxyribonucleoside thymidine. Similar to rNTP treatment, nucleoside supplementation resulted in the complete rescue of mitochondrial transcript levels in cells lacking NME6 (Fig 5C). The rescue of mitochondrial transcripts by nucleoside treatment correlated with a complete restoration of normal OCR and ECAR in *NME6* knockout cells (Fig 5D), demonstrating that exogenous nucleoside supply is sufficient to maintain OXPHOS in the absence of NME6.

**Figure EV5:**
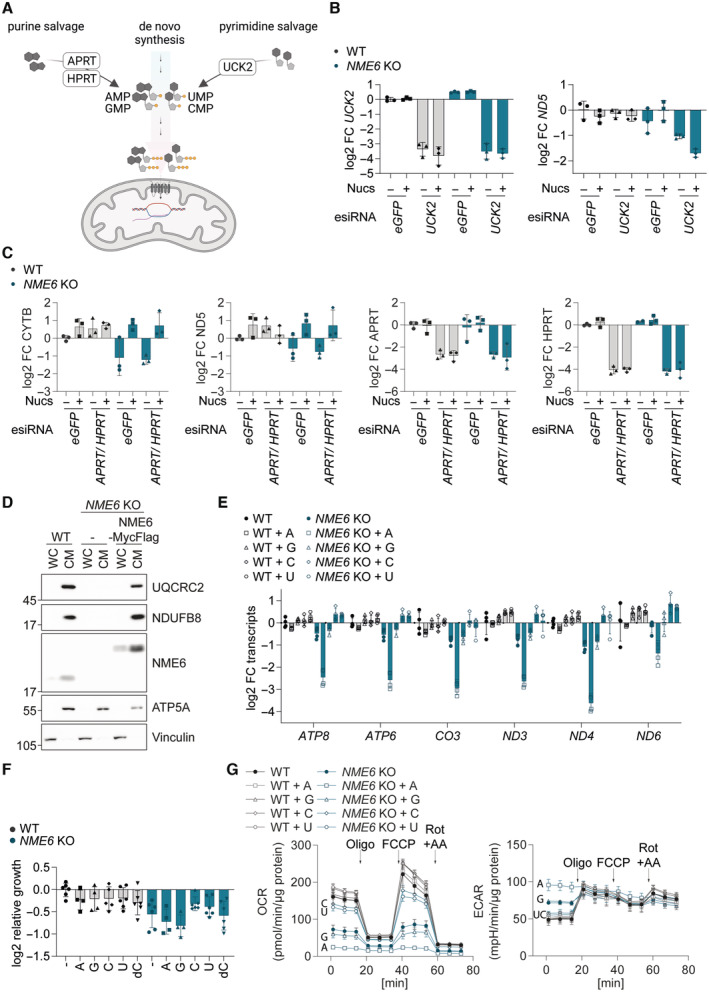
NME6 supplies pyrimidines for mitochondrial transcription and OXPHOS Scheme of the cytosolic ribonucleotide salvage pathway.
*UCK2* and *ND5* transcript levels analysed by qRT‐PCR in WT and *NME6* KO HeLa cells transfected with the indicated esiRNA and incubated with or without nucleosides (100 μM) for 72 h (log2; *n* = 3 independent cultures from Fig 5E).
*CYTB*, *ND5*, *APRT* and *HPRT* transcript levels analysed by qRT‐PCR in WT and *NME6* KO HeLa cells transfected with the indicated esiRNA and incubated with or without nucleosides (100 μM) for 72 h (log2; *n* = 3 independent cultures).Representative immunoblot showing the enrichment of mitochondrial proteins in crude mitochondrial fractions taken from the indicated HeLa cell lines during the isolation of mitochondria for LC–MS based metabolomics in Fig 5F. WC, whole cell; CM, crude mitochondria.Mitochondrial transcript levels analysed by qRT‐PCR in WT and *NME6* KO HeLa cells incubated with the indicated nucleoside species for 48 h as in Fig 5G (A, adenosine; G, guanosine; C, cytidine; U, uridine; log2 transformed; 100 μM; *n* = 4 independent cultures).The growth rates of HeLa WT and *NME6* KO cells supplemented with individual nucleosides (A, adenosine; G, guanosine; C, cytidine; U, uridine; log2 transformed; 100 μM; *n* = 6 independent cultures).Oxygen consumption rates (OCR) and extracellular acidification rates (ECAR) of WT and *NME6* KO HeLa cells incubated with or without individual nucleosides for a minimum of 120 h. Mitochondrial stress test was performed as in Fig 3A (*n* = 3 independent experiments). The basal and maximal respiration rates shown in Fig 5H were determined from OCR measurements before injection of oligomycin and after injection of FCCP respectively (A, adenosine; G, guanosine; C, cytidine; U, uridine; 100 μM; *n* = 3 independent cultures).

We reasoned that exogenous nucleosides must be phosphorylated via the cytosolic salvage pathway prior to entry into the mitochondria as rNTPs in order to bypass the requirement for NME6 (Fig EV5A). Indeed, nucleoside treatment no longer restored mitochondrial transcript levels in *NME6* knockout cells that were depleted of the cytosolic uridine‐cytidine kinase 2 (UCK2), which is essential for pyrimidine nucleoside salvage by phosphorylating uridine and cytidine (Figs 5E and EV5A and B). In contrast, combined depletion of the purine nucleoside salvage enzymes, hypoxanthine‐guanine phosphoribosyl‐transferase (HPRT) and adenine phosphoribosyltransferase (APRT), did not prevent the restoration of mitochondrial transcripts in *NME6* knockout cells treated with nucleosides (Fig EV5C). Together, these data demonstrate that NME6 is required to generate pyrimidine rNTPs for mitochondrial transcription and OXPHOS.

We next quantified the abundance of nucleotide species in whole‐cell extracts and mitochondrial fractions by liquid chromatography‐mass spectrometry (LC–MS; Figs 5F and EV5D). Mitochondria lacking NME6 had significantly reduced levels of CTP (down by 70%) and dCTP (down by 90%), which were restored in cells re‐expressing NME6. Conversely, UTP and GTP levels were barely affected, while ATP and dATP were even moderately increased in mitochondria lacking NME6 (Fig 5F). This result suggested that NME6 is predominantly required for the maintenance of mitochondrial cytidine triphosphates. Consistently, treatment of cells with cytidine or uridine restored mitochondrial transcript levels in the absence of NME6, whereas purine nucleosides did not (Figs 5G and EV5E). Uridine treatment can increase both cellular UTP and CTP levels (Pooler *et al*, 2005), since UTP is readily converted to CTP by the cytosolic enzyme CTP synthetase (CTPS). We quantified mitochondrial nucleotides in cells supplemented with cytidine or uridine by LC–MS and confirmed that both treatments elevated mitochondrial CTP and dCTP levels in *NME6* knockout cells (Fig 5H; Appendix Fig S1A–D). Neither nucleoside could fully restore mitochondrial CTP/dCTP in *NME6* knockout cells however, which may explain the partial improvement in cell growth upon treatment with cytidine or uridine (Fig EV5F).

**Figure 6:**
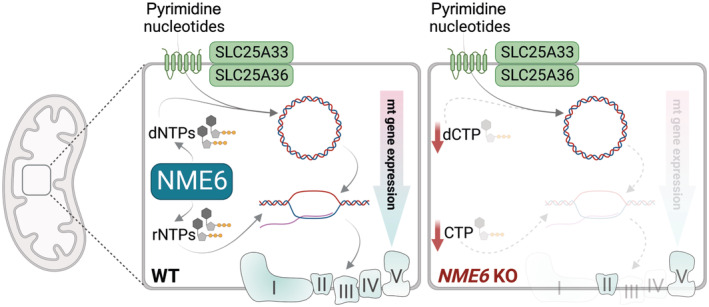
Mitochondrial pyrimidine nucleotide synthesis is required for mitochondrial gene expression Schematic depiction of the central role played by NME6 in mitochondrial nucleotide metabolism.

Finally, we tested the impact of individual nucleoside supplementation on mitochondrial respiration and observed that cytidine or uridine treatment resulted in near complete restoration of OCR in *NME6* knockout HeLa cells (Figs 5I and EV5G). The ECAR in *NME6* knockout cells was also normalised upon treatment with the pyrimidine nucleosides, likely reflecting a deceleration of glycolysis (Figs 5I and EV5G). Conversely, treatment of these cells with guanosine did not restore mitochondrial respiration or ECAR, while adenosine treatment resulted in a more severe inhibition of mitochondrial respiration and a further increase in ECAR in cells lacking NME6. The bioenergetic impact of each nucleoside correlated remarkably with their individual effects on mitochondrial transcript levels in *NME6* knockout cells (Figs 5G and EV5E) and nucleoside treatment had no impact on the bioenergetics (Figs 5I and EV5G) or mitochondrial transcript levels in WT HeLa cells (Figs 5G and EV5E). These results highlight a specific dependency on NME6 for the maintenance of mitochondrial pyrimidine nucleotides required to drive mitochondrial gene expression and OXPHOS in proliferating cells (Fig 6).

## Discussion

We reveal the dual function of the nucleoside diphosphate kinase NME6 in mitochondrial nucleotide metabolism. NME6 supplies pyrimidine rNTPs for mitochondrial transcription and is required for the maintenance of mtDNA when pyrimidine dNTP import from the cytosol is limited. The loss of NME6 or of its kinase activity impairs mitochondrial gene expression and OXPHOS function, despite normal levels of mtDNA. Therefore, while previous research has focused on the supply of deoxyribonucleotides for mtDNA synthesis, our study highlights the importance of mitochondrial ribonucleotide metabolism for healthy mitochondrial function.

The inhibition of NME6 kinase activity results in mitochondrial cytidine nucleotide depletion and loss of OXPHOS subunits, which likely reflects the degradation of non‐assembled OXPHOS subunits upon a primary loss of mtDNA‐encoded subunits (Deshwal *et al*, 2020; Szczepanowska & Trifunovic, 2021). The specific depletion of CTP and dCTP in *NME6* knockout HeLa cells is surprising given the reported lack of base moiety specificity of NDPK enzymes (Lascu & Gonin, 2000). Interestingly, *in vitro* kinase assays that describe NME6 as an active NDPK used CDP as the γ‐phosphate acceptor with recombinant NME6 (Tsuiki *et al*, 1999), whereas no NDPK activity was detected for recombinant NME6 when dTDP was used as the γ‐phosphate acceptor (Proust *et al*, 2021). It remains to be seen whether the contribution of NME6 to the steady‐state levels of mitochondrial ribonucleotides differs between cell and tissue types, as indicated by the different effects of NME6 in various liver cancer cell lines. The depletion of mitochondrial CTP may lead to the stalling of the mitochondrial RNA polymerase, analogous to DNA polymerase stalling upon the depletion of dNTPs, and ultimately perturb transcription efficiency (Edenberg *et al*, 2014). The strongest effect of NME6 loss on mitochondrial transcripts furthest from the heavy‐strand promoter likely reflects progressive CTP depletion with ongoing transcription and argues against a general role for NME6 in mRNA stabilisation. For comparison, all heavy‐strand mRNA transcripts are depleted in mitochondria lacking the mRNA stabilising factor, leucine‐rich pentatricopeptide repeat containing (LRPPRC) protein (Gohil *et al*, 2010; Ruzzenente *et al*, 2012). Nevertheless, mRNA stability will contribute to the steady state levels of transcripts after NME6 depletion and may explain the subtle differences we observed in relative mRNA changes upon NME6 loss between HeLa and HLE cells. Interestingly, our proteomic analysis revealed that the levels of four mitochondrial proteins associated with mitochondrial RNA granules (MRGs) are reduced in the absence of NME6: ALKBH1, ERAL1, DHX30 and RCC1L (Antonicka & Shoubridge, 2015; Antonicka *et al*, 2017; Zaganelli *et al*, 2017; Wagner *et al*, 2019). MRGs are hubs for the processing and maturation of nascent RNA and are associated with the assembly of mitoribosomes (Xavier & Martinou, 2021). In light of the critical role of NME6 for transcription, it is intriguing to consider that its interaction with RCC1L may allow spatial coordination of mitochondrial transcription with translation.

NME6 is not required for the maintenance of mtDNA in the cell lines we tested despite a significant reduction in mitochondrial dCTP levels detected in *NME6* knockout HeLa cells. Mitochondria contain asymmetric pools of dNTPs and HeLa cells have been reported to have low levels of dCTP in comparison to other dNTP species (Song *et al*, 2003). Depletion of dCTP is also linked to mtDNA depletion in mitochondrial neurogastrointestinal encephalomyopathy (MNGIE) caused by mutations in thymidine phosphorylase (TYMP) (González‐Vioque *et al*, 2011). We speculate that proliferating cells lacking NME6 are still able to maintain sufficient dCTP supply for mtDNA replication via the import of cytosolic dCTP. NME6 nevertheless becomes essential for the maintenance of mtDNA in cells lacking the mitochondrial pyrimidine transporters, SLC25A33 and SLC25A36. It remains to be seen whether NME6 is required for mtDNA synthesis in quiescent tissues that suppress *de novo* synthesis of deoxyribonucleotides and depend on mitochondrial nucleotide salvage for the provision of dNTPs for mtDNA synthesis (Ferraro *et al*, 2005; Mathews & Song, 2007; Pontarin *et al*, 2007). Reduced mitochondrial dCTP may also cause multiple mutations and deletions in the mtDNA of proliferating cells (Song *et al*, 2003; González‐Vioque *et al*, 2011), which should be explored in future studies alongside the absolute concentrations of mitochondrial dNTPs in the presence and absence of NME6.

Synthesis of mitochondrial pyrimidines by NME6 has broad implications for the control of OXPHOS and mitochondrial signalling. For instance, NME6 is upregulated along with the mitochondrial transcription machinery in certain cases of liver cancer and has been linked to negative prognosis (Jiang *et al*, 2019; Fei *et al*, 2020; Wan *et al*, 2021). It will also be important to consider how NME6 may influence mitochondrial signalling in innate immunity. Altered mitochondrial pyrimidine metabolism can trigger inflammation (Sprenger *et al*, 2021) and NME6 was recently identified as a positive regulator of the inflammasome in a mouse macrophage cell line, along with NME4 and NME3 (Ernst *et al*, 2021). Expanding our understanding of mitochondrial nucleotide metabolism will be essential to understand how mitochondrial nucleotide supply impacts ageing and disease associated with dysregulation of the mitochondrial genome and OXPHOS function. We propose that mitochondrial ribonucleotide salvage and mtRNA synthesis should be considered alongside mtDNA synthesis pathways in the context of diseases associated with defective mitochondrial nucleotide metabolism.

## Materials and Methods

### Reagents and Tools table

**Table:** 

Reagent/Resource	Source	Identifier/Catalog number
**Antibodies**		
Human SLC25A33	Origene	TA309042
Human SLC25A36	Gene Tex	GTX119934
Human MT‐CO2	Abcam	ab110258
Human SDHA	Abcam	ab14715
Human Oxphos‐Cocktail	Abcam	ab110411
Human NME6	Sigma	HPA017909
Human RCC1L	Proteintech	13796‐1‐AP
Human TFAM	Proteintech	22586‐1‐AP
DNA	Sigma	CBL186
BrdU	Abcam	ab6326
HRP‐conjugated secondary anti rabbit	BioRad	170‐6515
HRP‐conjugated secondary anti mouse	BioRad	170‐6516
Alexa 488‐conjugated secondary anti rabbit	Invitrogen	A11034
Alexa 568‐conjugated secondary anti mouse (IgM)	Invitrogen	A21043
Alexa 568‐conjugated secondary anti rat	Invitrogen	A11077
**qPCR oligonucleotides**		
MT‐ND1	Thermo Fisher Scientific	Hs02596873_s1
MT‐ND2	Thermo Fisher Scientific	Hs02596874_g1
MT‐ND3	Thermo Fisher Scientific	Hs02596875_s1
MT‐ND4	Thermo Fisher Scientific	Hs02596876_g1
MT‐ND5	Thermo Fisher Scientific	Hs02596878_g1
MT‐ND6	Thermo Fisher Scientific	Hs02596879_g1
MT‐CYTB	Thermo Fisher Scientific	Hs02596867_s1
MT‐CO1	Thermo Fisher Scientific	Hs02596864_g1
MT‐CO2	Thermo Fisher Scientific	Hs02596865_g1
MT‐CO3	Thermo Fisher Scientific	Hs02596866_g1
MT‐ATP6	Thermo Fisher Scientific	Hs02586862_g1
MT‐RNR1	Thermo Fisher Scientific	Hs02596859_g1
MT‐RNR2	Thermo Fisher Scientific	Hs02596860_s1
B2M	Thermo Fisher Scientific	Hs00187842_m1
Genomic GAPDH	Thermo Fisher Scientific	Hs02786624_g1
Genomic ACTB (actin β)	Thermo Fisher Scientific	Hs03023880_g1
NDUFA7	Thermo Fisher Scientific	Hs01561430_m1
NDUFS4	Thermo Fisher Scientific	Hs00159589_m1
NDUFB8	Thermo Fisher Scientific	Hs00922353_g1
UQCRB	Thermo Fisher Scientific	Hs00559884_m1
COX7A2L	Thermo Fisher Scientific	Hs01059547_g1
MT‐7S (Dloop)	Thermo Fisher Scientific	Hs02596861_s1
UCK2	Thermo Fisher Scientific	Hs00989900_m1
APRT	Thermo Fisher Scientific	Hs00975727_g1
HPRT	Thermo Fisher Scientific	Hs02800695_m1
**esiRNA oligonucleotides**		
Human UCK2 esiRNA	Sigma	EHU153241
Human SLC25A33	Sigma	EHU160961
Human SLC25A36	Sigma	EHU036611
Human APRT	Sigma	EHU1236681
Human HPRT	Sigma	EHU078931
EGFP	Sigma	EHUEGFP
**Plasmid oligonucleotides**		
SLC25A33 gRNA px459v2	Genscript	U7363GD130_1
SLC25A36 gRNA px459v2	Genscript	U721DGA050_1
NME6 gRNA px459v2	Genscript	U197WGG080_2
NME4 gRNA	This study	gacccgggagcggaccctgg
RCC1L gRNA #1	This study	caccgcggatgttacgaaagtctgg
RCC1L gRNA #2	This study	caccgaggggctacgagtatgtgt
hNME6‐MYC‐FLAG pCMV6‐Entry	Origene	RC20051
pLVX‐puro	Takara	632164
hNME6‐MYC‐FLAG pLVX‐puro	This study	
hNME6 H137N‐MYC‐FLAG pLVX‐puro	This study	
**Cell lines**		
HeLa	ATCC	CCL2
HLE	Japanese Collection of Research Biosources	JCRB0404
Huh6	Japanese Collection of Research Biosources	JCRB0401
HepG2	ATCC	HB‐8065
LentiX HEK293T	Takara	632180
**Cell culture reagents**		
Dulbecco's Modified Eagle's Medium	Gibco	61965
Dulbecco's Modified Eagle's Medium (glucose free)	Gibco	11966
Human Plasma Like Medium	Gibco	A48991‐01
Fetal Bovine Serum	Gibco	10270
Dialyzed FBS	Gibco	26400‐044
Galactose	Serva	22020.02
Glucose	Sigma	G7021
Glutamine	Gibco	25030‐081
Sodium Pyruvate	Gibco	11360
rNTPs	New England BioLabs	N0466S
dNTPs	New England BioLabs	N0446S
EmbryoMax 100× Nucleosides	Millipore	ES‐008‐D
Adenosine	Sigma	A4036
Guanosine	Sigma	G6264
Cytidine	Sigma	C4654
Uridine	Sigma	U3003
Lipofectamine RNAi MAX	Thermo Fisher Scientific	13778150
Lipofectamine CRISPR MAX	Thermo Fisher Scientific	CMAX00008
SYTOX green	Invitrogen	S7020
^35^S Methionine and Cysteine	Hartmann Analytics	ARS‐0110A
HCS CellMask Deep Red Stain	Thermo Fisher Scientific	H32721
DAPI	Sigma	D9542
MitoTracker Deep Red	Thermo Fisher Scientific	M22426
**Other**		
TaqMan PCR master mix	Thermo Fisher Scientific	4324020
Cell Titer Glo	Promega	G9241
Mitochondrial Stress Test	Agilent	103015‐100
NucleoSpin RNA	Machery‐Nagel	740955.250
DNeasy Blood and Tissue Kit	Qiagen	69506
GoScript Reverse Transcription	Promega	A2791
Bio‐Rad Protein Assay Kit	Bio‐Rad	5000001
Pierce Protein Assay Reagent	Thermo Fisher Scientific	22660
FluorSave Reagent	Millipore	345789

### Methods and Protocols

#### Reagents

Antibodies, qPCR oligonucleotides, esiRNA oligonucleotides, plasmids, cell lines, cell culture reagents and commercial assays used in this study are listed in the Reagents and Tools table.

#### Cell culture

HeLa, HLE, HepG2 and Huh6 cells were grown in Dulbecco's Modified Eagle's Medium (DMEM) containing 10% foetal bovine serum (FBS) and maintained at 37°C and 5% CO_2_, if not stated otherwise. Alternatively, cells were cultured in either glucose free DMEM supplemented with 10% FBS, 10 mM galactose and uridine (200 μg/ml), or in human plasma‐like medium (HPLM) supplemented with 10% dialysed FBS. All cultured cell lines were routinely tested for *Mycoplasma* contamination and authenticated by STR profiling.

For supplementation experiments, nucleosides (100 μM), rNTPs (100 μM) or dNTPs (100 μM) were added to the medium for at least 48 h. RNA interference experiments were performed by reverse transfection of 2 × 10^5^ cells with 5 μg esiRNA using Lipofectamine RNAiMax.

#### Generation of cell lines

Knockout (KO) cells were generated using CRISPR‐SpCas9 mediated gene editing. SpCas9 and guide RNA (gRNA) were expressed using transient transfection of px459 v2 expression vector (Genscript). Polyclonal cultures were obtained by puromycin selection prior to monoclone selection by serial dilution. Polyclonal cultures and individual clones were validated by immunoblotting and genomic sequencing. HeLa and HLE NME6 KO cells expressing NME6‐MycFlag or NME6 H137N‐Myc‐Flag were generated by lentiviral transduction. Lenti‐X HEK293T cells were transfected with either pLVX‐NME6‐Myc‐Flag or pLVX‐NME6^H137N^ Myc‐Flag using Lenti‐X Packaging Single Shots (Takara). The viral supernatant was collected after 48 h, cleared from cell debris by centrifugation and added to HeLa NME6 KO cells together with polybrene (4 μg/ml). Virus‐containing medium was removed after 24 h and puromycin selection (1 μg/ml) was started after an additional 24–48 h.

#### Cell proliferation assays

HeLa cell proliferation was monitored by live cell imaging using the Incucyte S3 instrument (Sartorius). Image analysis was performed using Incucyte Software 2019 RevB. 5 × 10^3^ cells per well were seeded onto a 96 well plate and confluency was assessed every 6 h by phase contrast imaging until 100% confluency was reached. Proliferation rates were determined from the slope of the exponential growth phase (24–72 h). Cell death was visualised by SYTOX green. SYTOX green was added to the assay medium (1:30,000) and relative cell death was calculated by area of SYTOX green puncta divided by phase contrast cell area. Relative growth of HLE, Huh6 and HepG2 cells was determined on each day after 5 × 10^3^ cells were seeded per well of a 96‐well plate. Total ATP luminescence was measured with the Cell Titer Glo viability assay (Promega) using a Glomax luminometer (Promega).

#### DNA extraction, RNA extraction and cDNA synthesis

For mitochondrial DNA (mtDNA) measurements, genomic DNA was isolated for cell pellets using the Blood and Tissue DNA extraction kit (Qiagen). RNA was isolated from cell pellets using the RNA extraction kit (Macherey‐Nagel) and 1–2 μg of RNA was reverse transcribed into cDNA using GoScript (Promega).

#### Cell lysis and SDS‐PAGE

Cells were collected in ice‐cold phosphate‐buffered saline (PBS). Cell pellets were lysed in RIPA buffer (50 mM Tris–HCl pH 7.4, 150 mM NaCl, 1% Triton ×100, 0.5% DOC, 0.1% SDS and 1 mM EDTA) for 30 min at 4°C. Lysates were cleared by centrifugation at 20,000 × *g* for 10 min at 4°C. Protein concentration was determined by Bradford assay (Bio‐Rad). Protein lysates were mixed with 4× Laemmli buffer and analysed by 10% SDS‐PAGE and immunoblot.

#### Quantitative PCR

For mtDNA measurements, 10 ng of genomic DNA were amplified using TaqMan PCR master mix (Thermo Fisher Scientific). MtDNA levels were assessed by the delta delta ct method using *MT‐7S*, *CYTB* and *MT‐ND6* as mitochondrial probes and *GAPDH* and *ACTB* as nuclear DNA controls. For the measurements of nuclear and mitochondrial transcripts, 10 ng of cDNA were amplified using TaqMan PCR master mix. Expression levels were calculated by the delta delta ct method, for which *B2M* was used as control.

#### Design of arrayed single guide RNA (sgRNA) library

The custom Mito Transporter and Salvage Pathway sgRNA library was purchased from Synthego and consisted of three sgRNA sequences designed to generate deletions in early exons of each target gene. The 116 target genes included all genes encoding proteins designated as “Small Molecule Transporters” in MitoCarta 3.0 (Rath *et al*, 2021) as well as putative mitochondrial metabolite carriers identified in a recent proteomic evaluation of mitochondria (Morgenstern *et al*, 2021) and solute carriers with proposed mitochondrial localisation (Meixner *et al*, 2020). Mitochondrial pyrimidine salvage pathway genes were selected in addition to *TFAM* and controls, including non‐targeting sgRNA (*NTC1*) and Polo‐like kinase 1 (*PLK1*). All target genes and sgRNA sequences are listed in Dataset EV1.

#### Arrayed CRISPR‐SpCas9 screen

The sgRNA library was reconstituted to 5 μM in Tris‐EDTA (pH 8.0) and distributed to 96‐well daughter plates. Note that the volumes in the following procedure correspond to individual transfections per well and were scaled up for the entire library in triplicate. Cas9 solution (0.5 μl SpCas9 2NLS nuclease, 1 μl Lipofectamine Cas9 plus reagent, 10.5 μl OptiMEM) was added to each well containing 1 μl sgRNA using the XRD‐384 automated reagent dispenser (Fluidx). The Cas9‐sgRNA mix was next stamped onto Cell Carrier Ultra 96‐well plates (PerkinElmer) in triplicate prior to addition of the transfection reagent (0.35 μl Lipofectamine CRISPRMax, 10.15 μl OptiMEM) using the XRD‐384. Each plate was placed on an orbital shaker at 300 rpm for 10 min at room temperature. In parallel, a cell suspension of *SLC25A33*/*SLC25A36* DKO HeLa cells (clone #2) was prepared in DMEM + 10% FBS (4 × 10^4^ cells/ml) and added (100 μl/well) to the sgRNA:Cas9:LipofectamineCRISPRMax transfection mixture. Cells were distributed and incubated at 37°C and 5% CO_2_. After 6 h, the media and transfection mix was replaced with fresh media containing 150 μM uridine using a plate washer (BioTekELx405) to aspirate and XRD‐384 to dispense. At 96 h post‐transfection, the media was replaced with 80 μl of 4% formaldehyde in DMEM for 10 min. The cells were then washed twice in 150 μl PBS and permeabilised with 0.1% Triton‐TX100 in 80 μl PBS for 20 min prior to two further PBS washes. Primary antibody staining was performed sequentially with anti‐TFAM (ProteinTech; 1:800) and anti‐DNA (Sigma; 1:800) antibodies in 40 μl PBS for 30 min at room temperature. Secondary antibody staining was also performed sequentially with anti‐rabbit IgG‐Alexa 488 nm (Thermo Fisher Scientific; 1:1,000) and anti‐mouse IgM‐Alexa 568 nm (Thermo Fisher Scientific; 1:1,000) antibodies in 40 μl PBS for 30 min at room temperature. Each antibody staining was followed by three PBS washes. Finally, DAPI (Merk; 0.5 μg/ml) and HCS CellMask Deep Red (Thermo Fisher Scientific; 1:20,000) were combined in 200 μl PBS per well for 30 min prior to three final PBS washes. Plates were stored in the dark at 4°C with 200 μl PBS in each well prior to imaging.

Plates were imaged with an OperaPhenix High Content Analysis System (PerkinElmer) using a 20× objective (25 fields per well; single plane) and 63× water objective (24 fields per well; 5 Z‐planes with 1 μm separation). Imaging was performed using 405 nm (DAPI), 488 nm (TFAM), 561 nm (DNA) and 640 nm (CellMask) excitation. Maximum intensity projections were generated in each channel using all planes and analysis was performed using Harmony 4.9 High‐Content Imaging and Analysis Software (PerkinElmer). Nuclei masks were defined as DAPI positive structures above 30 μm^2^ in area and were counted to determine cell number. The 20× objective was used to calculate cell number per well and all other analysis was performed with the 63× objective. MtDNA was measured within HCS CellMask stained cytoplasmic regions upon exclusion of the nuclei. MtDNA intensity was analysed using standard mean intensity and mtDNA puncta were identified and measured using the “Find Spots” algorithm (relative spot intensity: > 0.045, splitting sensitivity: 1).

Across the three replicates, cell number was depleted by over 75% in cells transfected with the lethal control sgRNA (PLK1) compared to non‐targeting control sgRNA, which confirmed efficient transfection with Cas9:sgRNA ribonucleoprotein complexes in our screen.

#### Immunofluorescence and confocal microscopy

Cells were seeded on glass coverslips and grown to a confluency of 50%. MitoTracker Deep Red (Thermo Fisher Scientific; 100 nM) and bromouridine BrUX were added 20 min prior to fixation with 4% paraformaldehyde at 37°C. Cells were permeabilised and blocked in PBS containing 5% normal goat serum and 0.15% Triton‐X. Cells were stained with primary antibodies for 90 min (NME6 1:250; BrdU 1:200; DNA 1:200) followed by Alexa‐488 or Alexa‐568 conjugated secondary antibodies for 45 min (1:1,000). Coverslips were mounted onto glass slides using FluorSave Reagent (Millipore) and imaged using a Zeiss LSM 880 Airyscan Confocal microscope with Zen 2.3 SP1 acquisition software. The objective used was Plan‐Apochromat 40×/1.3 Oil DIC M27 and all images were acquired with the following three lasers: 633 nm (BP 570–620 + LP 645), 561 nm (BP 420–480 + BP 495–620) and 488 nm (BP 420–480 + BP 495–550). Plot profiles of foci were measured with ImageJ Version 1.53t.

#### Oxygen consumption and extracellular acidification measurements

Mitochondrial ATP‐linked respiration and extra cellular acidification rate was measured by the Seahorse XFe96 analyser using the Mito Stress Test kit (Agilent). 4 × 10^4^ cells were seeded and grown for 24 h in DMEM containing 10% FBS. For supplementation experiments, cells were cultured for at least 48 h in nucleoside containing medium prior to the experiment. Growth medium was exchanged to assay medium containing, glutamine, pyruvate and glucose. Oligomycin (2 μM), FCCP (0.5 μM) and rotenone and antimycin A (0.5 μM each) injections were used to calculate basal respiration, ATP‐linked ATP production and maximal respiration, respectively. Results were normalised to the amount of protein per well.

#### Mitochondrial isolation

Cells were collected in ice‐cold PBS. Cell pellets were resuspended in 1 ml ice‐cold mitochondrial isolation buffer (containing 220 mM mannitol, 70 mM sucrose, 5 mM HEPES‐KOH pH 7.4 and 1 mM EGTA‐KOH + complete protease inhibitor). Cells were lysed detergent‐free, using 10 strokes of a 1‐ml syringe equipped with a 27‐g needle. Cell lysates were spun down at 600 × *g* for 5 min at 4°C and the supernatant was separated from the remaining cell pellet. The supernatant was spun down for 10 min at 8,000 × *g* at 4°C. Mitochondria‐enriched pellets were used for IP‐, proteomic‐ and metabolomics experiments. Purity of the mitochondria‐enriched fractions was verified by SDS‐PAGE and immunoblot.

#### Immunoprecipitation

Mitochondria‐enriched pellets of HeLa WT and NME6 KO + NME6‐MycFlag expressing cells (500 μg) were resuspended in 500 μl IP buffer (60 mM Tris–HCl and 300 mM KAc‐KOH pH 7.4) Mitochondria were solubilised with digitonin (5 g/g protein) for 30 min at 4°C while shaking on a ThermoMixer (shaking: 550 rpm). Mitochondrial lysates were spun down at 20,000 × *g* for 15 min at 4°C. The supernatant was mixed with Flag‐agarose beads (Sigma) and incubated for 2 h at 4°C. After 2 h, the supernatant was removed by centrifugation at 500 × *g* for 30 s and the remaining beads were washed three times with wash buffer (IP buffer containing 0.1% digitonin). Bound proteins were eluted from the beads using 60 μl of 1× Laemmli buffer, samples were incubated for 10 min at 40°C. The eluate was separated from the beads by centrifugation at 1,000 × *g* for 3 min. Eluates were used for SDS‐PAGE, immunoblot analysis and LC–MS‐based proteomics.

#### Mitochondrial translation assay

Mitochondrial translation rates were monitored by incorporation rates of radioactive methionine and cysteine (^35^S) (Hartmann Analytic) into mitochondrial encoded proteins. Therefore, 3 × 10^5^ cells were cultured for 24 h. Growth medium was washed out and replaced with minimal medium depleted of methionine and cysteine. Cytosolic translation was blocked by emetine (100 μg/ml) for 30 min. ^35^S methionine and cysteine (50 μCi each) was added to the medium for 15–60 min. Cells were lysed and protein extracts were subjected to SDS‐PAGE. Incorporation rates were visualised by autoradiography using a Typhoon FLA9500 imager (GE healthcare). Mitochondrial proteins were labelled according to their respective molecular weight.

#### Extraction of polar metabolites

Cell pellets and mitochondrial pellets were resuspended in −20°C cold extraction buffer (HPLC‐grade ultrapure 40% MeOH, 40% acetonitrile, 20% water and 0.1 μg/ml ^13^C labelled ATP). Pellets were dissolved by sonication at 4°C followed by an incubation for 30 min at 4°C while shaking at 1,500 rpm. The metabolite containing supernatant was cleared by centrifugation at 20,000 × *g* for 10 min and subsequently transferred to a speedvac concentrator to fully evaporate the extraction buffer. The remaining protein pellet from the extraction was used to determine protein concentration of the sample.

#### Anion‐exchange chromatography mass spectrometry (AEX‐MS) for the analysis of nucleotides and deoxynucleotides

Extracted metabolites from crude‐ and mito‐preparations were resuspended in 100 μl of UPLC/MS grade water (Biosolve) and transferred to *polypropylene* autosampler vials (Chromatography Accessories Trott, Germany).

The samples were analysed using a Dionex ionchromatography system (Integrion Thermo Fisher Scientific) as described previously (Schwaiger *et al*, 2017). In brief, 5 μl of polar metabolite extract were injected in push partial mode, using an overfill factor of 1, onto a Dionex IonPac AS11‐HC column (2 mm × 250 mm, 4 μm particle size, Thermo Fisher Scientific) equipped with a Dionex IonPac AG11‐HC guard column (2 mm × 50 mm, 4 μm, Thermo Fisher Scientific). The column temperature was held at 30°C, while the auto sampler was set to 6°C. A potassium hydroxide gradient was generated using a potassium hydroxide cartridge (Eluent Generator, Thermo Scientific), which was supplied with deionised water (Millipore). The metabolite separation was carried at a flow rate of 380 μl/min, applying the following gradient conditions: 0–3 min, 10 mM KOH; 3–12 min, 10–50 mM KOH; 12–19 min, 50–100 mM KOH; 19–22 min, 100 mM KOH, 22–23 min, 100–10 mM KOH. The column was re‐equilibrated at 10 mM for 3 min.

For the analysis of metabolic pool sizes the eluting compounds were detected in negative ion mode using full scan measurements in the mass range *m/z* 77–770 on a Q‐Exactive HF high resolution MS (Thermo Fisher Scientific). The heated electrospray ionisation (HESI) source settings of the mass spectrometer were: Spray voltage 3.2 kV, capillary temperature was set to 300°C, sheath gas flow 50 AU, aux gas flow 20 AU at a temperature of 330°C and a sweep gas glow of 2 AU. The S‐lens was set to a value of 60.

The semi‐targeted LC–MS data analysis was performed using the TraceFinder software (Version 5.1, Thermo Fisher Scientific). The identity of each compound was validated by authentic reference compounds, which were measured at the beginning and the end of the sequence. For data analysis the area of the deprotonated [M‐H^+^]^−1^ or doubly deprotonated [M‐2H]^−2^ mono‐isotopologue mass peaks of every required compound were extracted and integrated using a mass accuracy < 3 ppm and a retention time tolerance of < 0.05 min as compared to the independently measured reference compounds. These areas were then normalised to the internal standard, which was added to the extraction buffer, followed by a normalisation to the protein content of the analysed sample. Values were log2 transformed and normalised to the WT mean.

#### Sample preparation for mass spectrometry‐based proteomics

For whole proteome analysis, 60 μl of 4% SDS in 100 mM HEPES‐KOH (pH = 8.5) was pre‐heated to 70°C and added to the cell pellet for further 10 min incubation at 70°C on a ThermoMixer (shaking: 550 rpm). The protein concentration was determined using the 660 nm Protein Assay (Thermo Fisher Scientific, #22660). 20 μg of protein was subjected to tryptic digestion. For immunoprecipitation analysis, the LDS buffer eluate was directly used. Proteins were reduced (10 mM TCEP) and alkylated (20 mM CAA) in the dark for 45 min at 45°C. Samples were subjected to an SP3‐based digestion (Hughes *et al*, 2014). Washed SP3 beads (Sera‐Mag (TM) Magnetic Carboxylate Modified Particles (Hydrophobic, GE44152105050250), Sera‐Mag™ Magnetic Carboxylate Modified Particles (Hydrophilic, GE24152105050250) from Sigma Aldrich) were mixed equally, and 3 μl of bead slurry were added to each sample. Acetonitrile was added to a final concentration of 50% and washed twice using 70% ethanol (*V* = 200 μl) on an in‐house made magnet. After an additional acetonitrile wash (*V* = 200 μl), 5 μl digestion solution (10 mM HEPES‐KOH pH = 8.5 containing trypsin (0.5 μg, Sigma) and LysC (0.5 μg, Wako)) was added to each sample and incubated overnight at 37°C. Peptides were desalted on a magnet using 2 × 200 μl acetonitrile. Peptides were eluted in 10 μl 5% DMSO in LC–MS water (Sigma Aldrich) in an ultrasonic bath for 10 min. Formic acid and acetonitrile were added to a final concentration of 2.5 and 2%, respectively. Samples were stored at −20°C before subjection to LC–MS/MS analysis.

#### Liquid chromatography and mass spectrometry

LC–MS/MS instrumentation consisted of an Easy‐LC 1200 (Thermo Fisher Scientific) coupled via a nano‐electrospray ionisation source to an Exploris 480 mass spectrometer (Thermo Fisher Scientific, Bremen, Germany). An in‐house packed column (inner diameter: 75 μm, length: 40 cm) was used for peptide separation. A binary buffer system (A: 0.1% formic acid and B: 0.1% formic acid in 80% acetonitrile) was applied as follows:

##### Whole proteome analysis

Linear increase of buffer B from 4 to 27% within 70 min, followed by a linear increase to 45% within 5 min. The buffer B content was further ramped to 65% within 5 min and then to 95% within 5 min. 95% buffer B was kept for a further 5 min to wash the column.

##### Immunoprecipitation analysis

Linear increase of buffer B from 4 to 27% within 40 min, followed by a linear increase to 45% within 5 min. The buffer B content was further ramped to 65% within 5 min and then to 95% within 5 min. 95% buffer B was kept for a further 5 min to wash the column.

Prior to each sample, the column was washed using 5 μl buffer A and the sample was loaded using 8 μl buffer A.

The RF Lens amplitude was set to 55%, the capillary temperature was 275°C and the polarity was set to positive. MS1 profile spectra were acquired using a resolution of 120,000 (at 200 *m/z*) at a mass range of 320–1,150 *m/z* and an AGC target of 1 × 10^6^.

For MS/MS independent spectra acquisition, 48 equally spaced windows were acquired at an isolation *m/z* range of 15 Th, and the isolation windows overlapped by 1 Th. The fixed first mass was 200 *m/z*. The isolation centre range covered a mass range of 357–1,060 *m/z*. Fragmentation spectra were acquired at a resolution of 15,000 at 200 *m/z* using a maximal injection time of 22 ms and stepped normalised collision energies (NCE) of 26, 28 and 30. The default charge state was set to 3. The AGC target was set to 3e6 (900% ‐ Exploris 480). MS2 spectra were acquired in centroid mode.

#### Proteomics data analysis

DIA‐NN (Data‐Independent Acquisition by Neural Networks) v 1.8 (Demichev *et al*, 2020) was used to analyse data‐independent raw files. The spectral library was created using the reviewed‐only Uniport reference protein (*Homo sapiens*, 20,350 entries, downloaded September 2019) with the “Deep learning‐based spectra and RTs prediction” turned on. Protease was set to trypsin and a maximum of 1 miss cleavage was allowed. N‐term M excision was set as a variable modification and carbamidomethylation at cysteine residues was set as a fixed modification. The peptide length was set to 7–30 amino acids and the precursor *m/z* range was defined from 340 to 1,200 *m/z*. The option “Quantitative matrices” was enabled. The FDR was set to 1% and the mass accuracy (MS2 and MS1) as well as the scan window was set to 0 (automatic inference via DIA‐NN). Match between runs (MBR) was enabled. The Neuronal network classifier worked in “double pass mode” and protein interference was set to “Isoform IDs”. The quantification strategy was set to “robust LC (high accuracy)” and cross‐run normalisation was defined as “RT‐dependent”.

The “pg” (protein group) output (MaxLFQ intensities; Cox *et al*, 2014) was further processed using Instant Clue (Nolte *et al*, 2018) including and pairwise comparison using an unpaired two‐sided *t*‐test or one‐way ANOVA followed by a permutation‐based FDR correction (5%).

MitoCarta 3.0 (Rath *et al*, 2021) and Uniprot‐based Gene Ontology annotations were used for filtering. Hierarchical clustering, heatmaps and volcano plots were generated using the InstantClue software (Nolte *et al*, 2018) v. 0.10.10.

## Author contributions

**Thomas MacVicar:** Conceptualization; resources; data curation; supervision; funding acquisition; validation; visualization; writing – original draft; project administration; writing – review and editing. **Thomas Langer:** Conceptualization; resources; supervision; funding acquisition; validation; writing – original draft; project administration; writing – review and editing. **Nils Grotehans:** Conceptualization; data curation; formal analysis; validation; investigation; visualization; methodology; writing – original draft; writing – review and editing. **Lynn McGarry:** Data curation; formal analysis; validation; visualization; methodology. **Hendrik Nolte:** Data curation; software; formal analysis; visualization; methodology; writing – review and editing. **Moritz Kroker:** Investigation; writing – review and editing. **Álvaro Jesús Narbona‐Pérez:** Conceptualization; methodology; writing – review and editing. **Soni Deshwal:** Conceptualization; methodology; writing – review and editing. **Patrick Giavalisco:** Conceptualization; data curation; formal analysis; validation; methodology; writing – review and editing. **Vanessa Xavier:** Data curation; visualization.

## Disclosure and competing interests statement

The authors declare that they have no conflict of interest.

## Supporting information



Appendix

Expanded View Figures PDF

Dataset EV1

Dataset EV2

Dataset EV3

Dataset EV4

PDF+

Source Data for Figure 1

Source Data for Figure 2

Source Data for Figure 3

Source Data for Figure 4

Source Data for Figure 5

## Data Availability

The mass spectrometry proteomics data have been deposited to the ProteomeXchange Consortium via the PRIDE partner repository with the dataset identifier PXD038391 (Perez‐Riverol *et al*, 2021).
