# Supplementary material for: Ribonucleotide synthesis by NME6 fuels mitochondrial gene expression
Source: EMBO J. 2023 Jul 13;42(18):e113256. doi: 10.15252/embj.2022113256 (PMC10505918; doi:10.15252/embj.2022113256)
Supplement: Supplementary file 1 — Appendix [file EMBJ-42-e113256-s010.pdf]

Table of contents

Appendix S1 …….2
Figure legend Appendix S1 …….3

Appendix S1

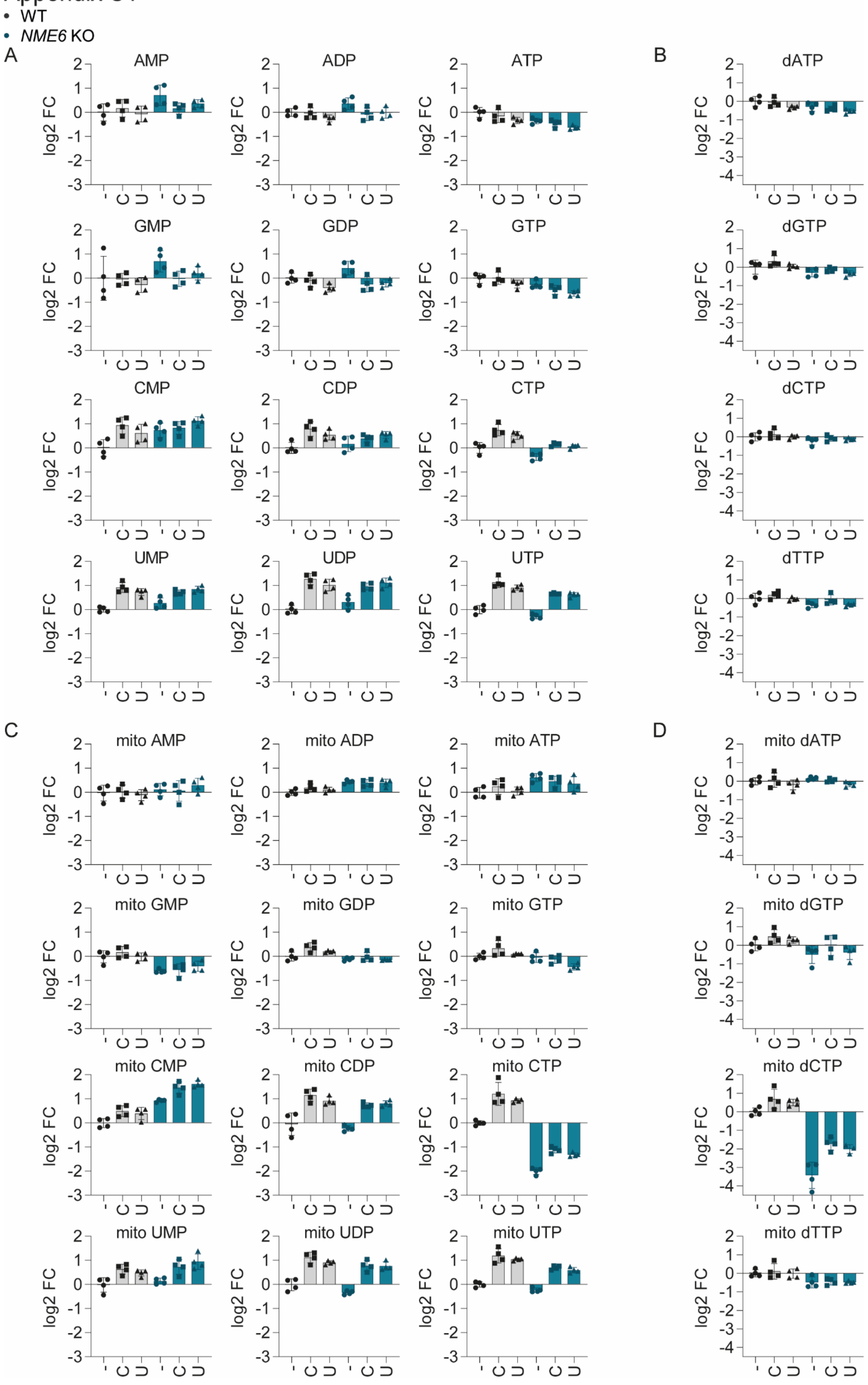

• WT
• *NME6* KO
A
B
C
D
AMP
ADP
ATP
dATP
GMP
GDP
GTP
dGTP
CMP
CDP
CTP
dCTP
UMP
UDP
UTP
dTTP
mito AMP
mito ADP
mito ATP
mito dATP
mito GMP
mito GDP
mito GTP
mito dGTP
mito CMP
mito CDP
mito CTP
mito dCTP
mito UMP
mito UDP
mito UTP
mito dTTP
log2 FC

Appendix S1 – Pyrimidine nucleoside supplementation improves nucleotide scarcity in *NME6* KO HeLa cells

(A) Ribonucleotide levels of whole cell extracts of WT and *NME6* KO HeLa cells incubated with the indicated nucleoside species for 120 h as determined by quantitative mass spectrometry.

(B) Deoxyribonucleotide triphosphate levels of whole cell extracts of WT and *NME6* KO HeLa cells incubated with the indicated nucleoside species for 120 h as determined by quantitative mass spectrometry.

(C) Ribonucleotide levels of mitochondria enriched fractions of WT and *NME6* KO HeLa cells incubated with the indicated nucleoside species for 120 h as determined by quantitative mass spectrometry.

(D) Deoxyribonucleotide triphosphate levels of mitochondria enriched fractions of WT and *NME6* KO HeLa cells incubated with the indicated nucleoside species for 120 h as determined by quantitative mass spectrometry.
(log2 fold change; n = 4 independent cultures)
