# Supplementary material for: Ribonucleotide synthesis by NME6 fuels mitochondrial gene expression
Source: EMBO J. 2023 Jul 13;42(18):e113256. doi: 10.15252/embj.2022113256 (PMC10505918; doi:10.15252/embj.2022113256)
Supplement: Supplementary file 2 — Expanded View Figures PDF [file EMBJ-42-e113256-s005.pdf]

Expanded View Figures

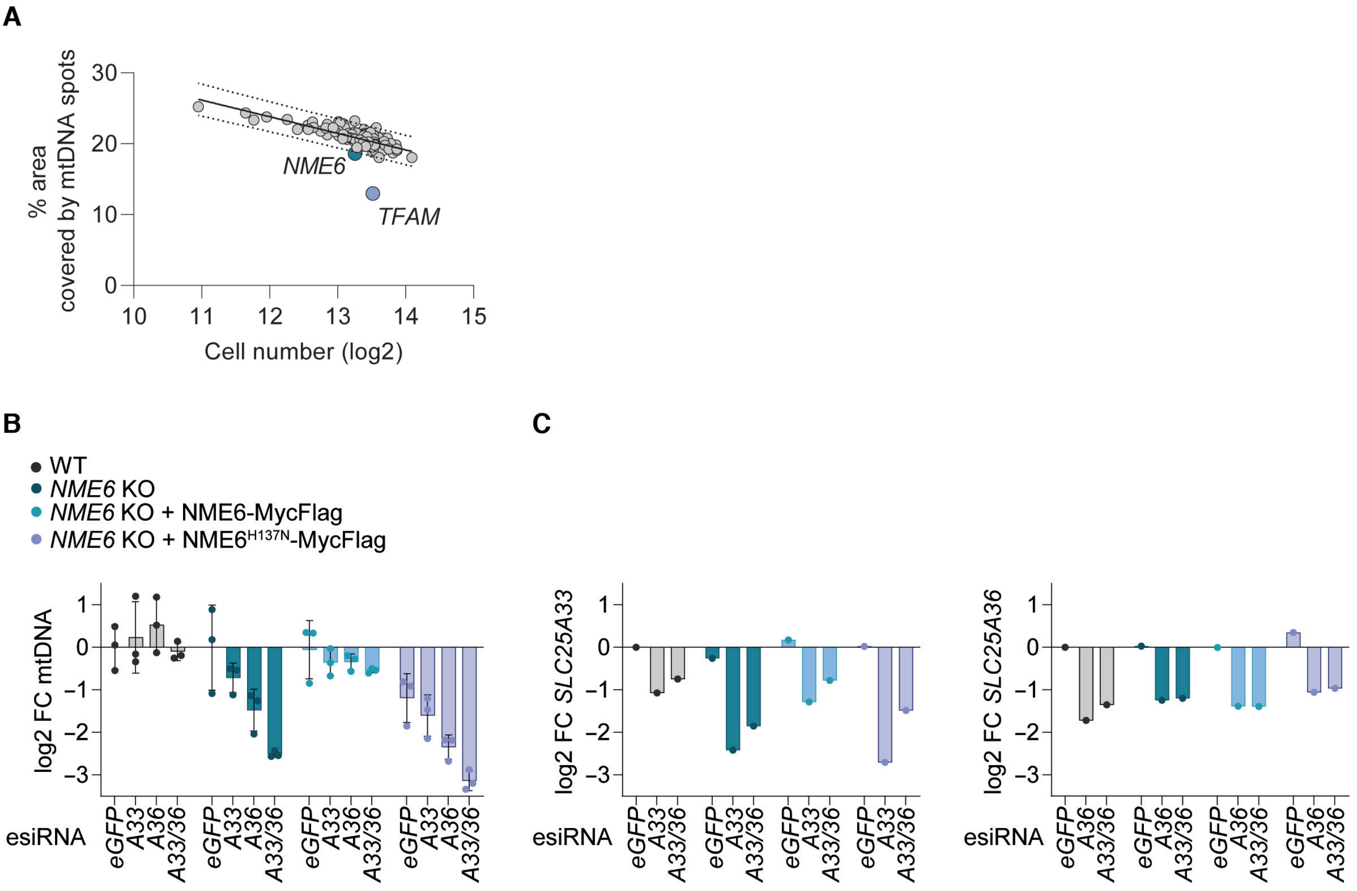

Figure EV1. CRISPR-SpCas9 screen analysis and validation by RNA interference.

A The result of the CRISPR-SpCas9 screen (Fig 1C) plotted as the percentage of cell cytoplasm area occupied by mtDNA spots against the mean log2 cell number from each sgRNA target. The values for cells transfected with *NME6* sgRNA and *TFAM* sgRNA lie below the 95% prediction bands (least-squares regression; R^2 = 0.52; n = 3 independent experiments).

B MtDNA level monitored by qPCR (*CYTB/ACTB*) in the indicated HeLa cell lines treated with esiRNA targeting *GFP* (control), *SLC25A33* (A33), *SLC25A36* (A36) or *SLC25A33* + *SLC25A36* (A33/A36) relative to WT cells (log2; n = 3 independent cultures).

C *SLC25A33* (left) and *SLC25A36* (right) transcript levels in samples from a representative experiment in (B) monitored by qRT-PCR (log2; n = 1).

Data information: FC, fold change. Data are means ± SD.

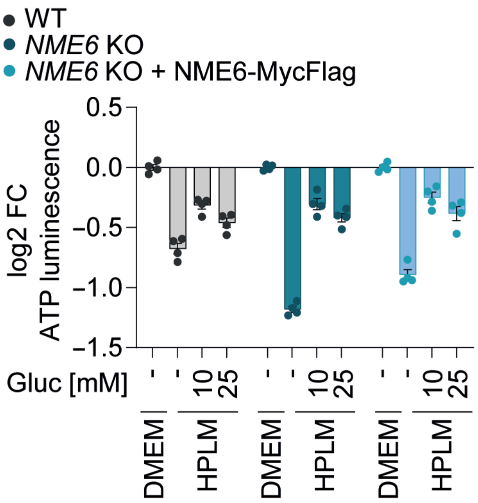

Figure EV2. Glucose is limiting for growth in HPLM.

Cell viability in WT, *NME6* KO and *NME6* KO + NME6 MycFlag HeLa cells incubated in HPLM supplemented with different concentrations of glucose (standard HPLM contains 5 mM glucose). Cell viability was determined by ATP luminescence assay and analysed relative to DMEM (log 2; n = 4 independent cultures). FC, fold change. Data are means ± SD.

Figure EV3. Alterations to the proteome upon NME6 depletion and further characterisation of NME6 in liver cancer.

A Extended unsupervised hierarchical row clustering (Euclidean distance, complete method) representation of significantly different proteins (FDR < 0.05) (*NME6* KO vs. WT) *z*-scores of log2-transformed protein intensities determined by quantitative mass spectrometry and filtered for mitochondrial proteins according to MitoCarta 3.0 (Rath *et al*, 2021). The cluster presented in Fig 3B is visible at the top (C4). The bottom cluster (C1) is expanded here to reveal proteins that are significantly upregulated in *NME6* KO cells compared to WT and *NME6* KO + NME6-MycFlag HeLa cells (*n* = 4 independent cultures).

B Relative NME6 protein expression in cell lines across the indicated cancer types determined by quantitative proteomic profiling (Nusinow *et al*, 2020). Data obtained from depmap.org/portal (solid line = median, box limits = 25th and 75th percentile and whiskers = maxima and minima).

C Kaplan–Meier plots showing patient survival in the indicated liver hepatocellular carcinoma cohorts (LIHC) from The Cancer Genome Atlas Program (TCGA) analysed using http://www.tcga-survival.com. *P*-values were determined using a log rank test (Smith & Sheltzer, 2022).

D Stratification of the gene effect of NME6 depletion from Fig 2A into cancer type (left, solid line = median, box limits = 25th and 75th percentile and whiskers = maxima and minima). Individual cell line *NME6* gene effects are also shown with HepG2 and Huh6 cell lines highlighted (right, note that HLE cells are not included in DepMap 22Q2 Public+Score, Chronos).

E Immunoblot analysis of WT HLE cells, *NME6* KO cells and *NME6* KO cells expressing NME6-MycFlag (WT) or NME6^{H137N}-MycFlag (H137N).

 © 2023 The Authors

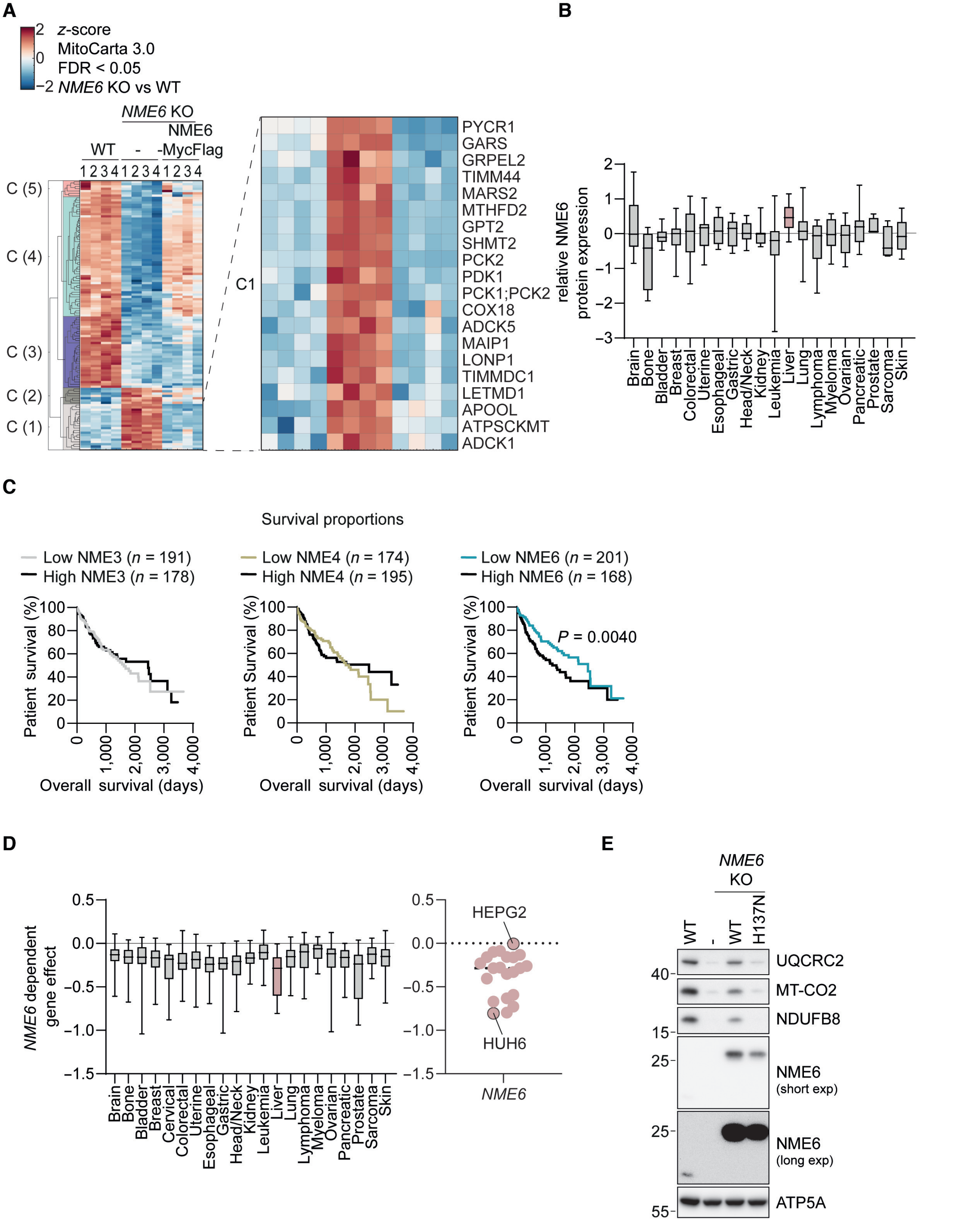
A
z-score
MitoCarta 3.0
FDR < 0.05
NME6 KO vs WT
NME6 KO
NME6
WT
-
-MycFlag
C (5)
C (4)
C (3)
C (2)
C (1)
C1
PYCR1
GARS
GRPEL2
TIMM44
MARS2
MTHFD2
GPT2
SHMT2
PCK2
PDK1
PCK1;PCK2
COX18
ADCK5
MAIP1
LONP1
TIMMDC1
LETMD1
APOOL
ATPSCKMT
ADCK1
B
relative NME6 protein expression
Brain
Bone
Bladder
Breast
Colorectal
Uterine
Esophageal
Gastric
Head/Neck
Kidney
Leukemia
Liver
Lung
Lymphoma
Myeloma
Ovarian
Pancreatic
Prostate
Sarcoma
Skin
C
Survival proportions
Low NME3 (n = 191)
High NME3 (n = 178)
Low NME4 (n = 174)
High NME4 (n = 195)
Low NME6 (n = 201)
High NME6 (n = 168)
Patient survival (%)
Patient Survival (%)
P = 0.0040
Overall survival (days)
D
NME6 dependent gene effect
HEPG2
HUH6
NME6
Brain
Bone
Bladder
Breast
Cervical
Colorectal
Uterine
Esophageal
Gastric
Head/Neck
Kidney
Leukemia
Liver
Lung
Lymphoma
Myeloma
Ovarian
Pancreatic
Prostate
Sarcoma
Skin
E
NME6 KO
WT
-
WT
H137N
UQCRC2
MT-CO2
NDUFB8
NME6 (short exp)
NME6 (long exp)
ATP5A

Figure EV3.

Figure EV4. Proteomic analysis of immunoprecipitates shows interaction between NME6 and RCC1L.

A Pan-cancer gene coessentiality network visualisation of the top 15 positively correlated genes (solid lines) and five secondary node positive interactions (dotted lines) with all NME family members (*NME1-9*) using FIREWORKS (Amici *et al*, 2021) (left) and *NME6* correlated genes were grouped further into manually annotated mitochondrial functions (right).

B Representative immunoblot (left) and proteomic analysis (right) following immunoprecipitation of NME6-MycFlag from HeLa cell mitochondrial lysates using a Flag antibody ($n = 4$ independent experiments). FC, fold change.

C Immunoblot analysis of HeLa WT cells and two polyclonal *RCC1L* KO populations.

D MtDNA-encoded transcript levels analysed by qRT-PCR in *NME6* KO, *NME6* KO + NME6-MycFlag and *NME6* KO H137N-MycFlag cells relative to WT HLE cells (log2; $n = 4$ independent cultures).

 © 2023 The Authors

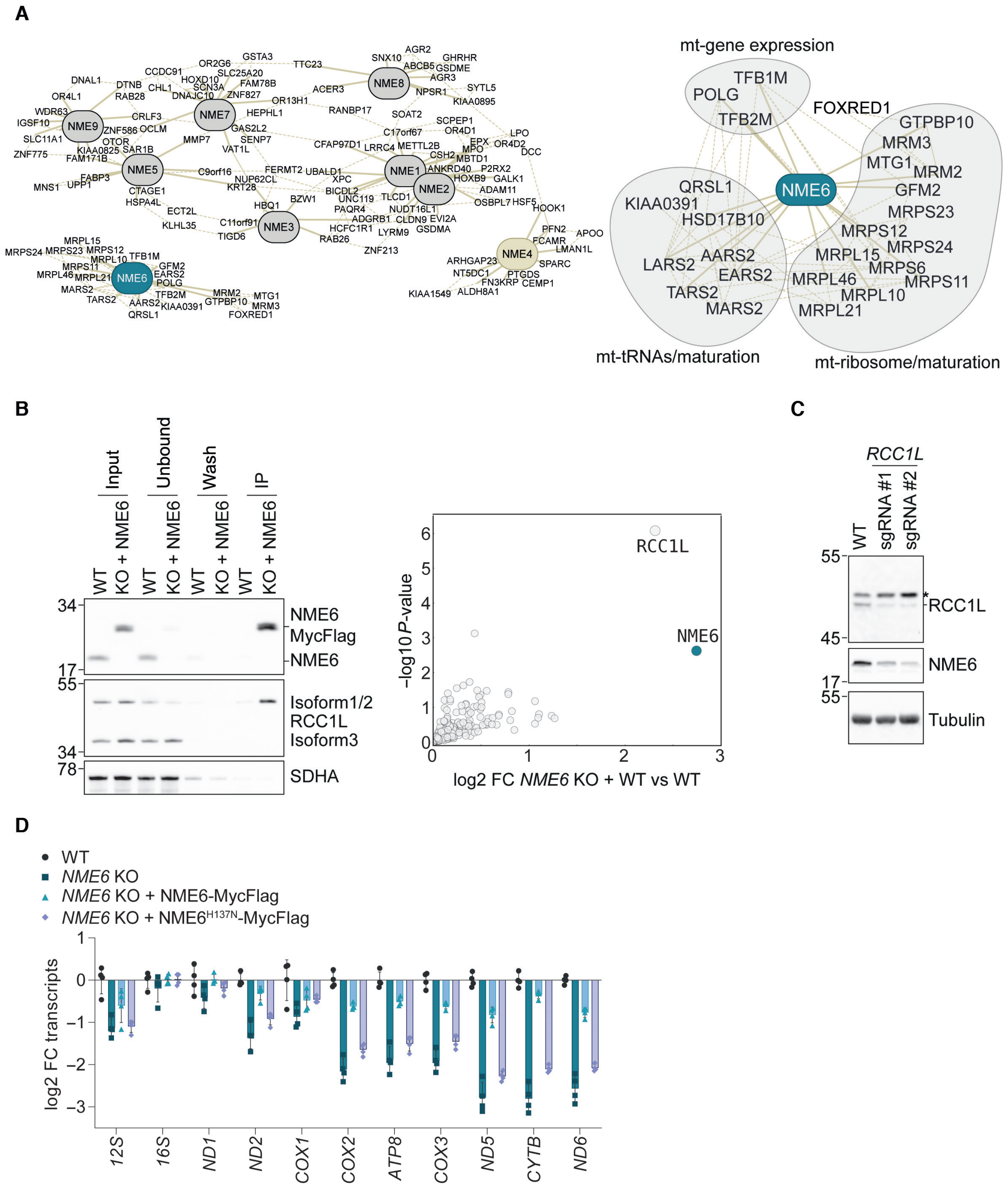
A
mt-gene expression
mt-tRNAs/maturation
mt-ribosome/maturation
B
Input
Unbound
Wash
IP
NME6
MycFlag
Isoform1/2
RCC1L
Isoform3
SDHA
RCC1L
NME6
log2 FC NME6 KO + WT vs WT
-log10 P-value
C
RCC1L
WT
sgRNA #1
sgRNA #2
Tubulin
D
WT
NME6 KO
NME6 KO + NME6-MycFlag
NME6 KO + NME6H137N-MycFlag
log2 FC transcripts
12S
16S
ND1
ND2
COX1
COX2
ATP8
COX3
ND5
CYTB
ND6

Figure EV4.

Figure EV5. NME6 supplies pyrimidines for mitochondrial transcription and OXPHOS.

A Scheme of the cytosolic ribonucleotide salvage pathway.

B *UCK2* and *ND5* transcript levels analysed by qRT-PCR in WT and *NME6* KO HeLa cells transfected with the indicated esiRNA and incubated with or without nucleosides (100 μM) for 72 h (log2; $n = 3$ independent cultures from Fig 5E).

C *CYTB*, *ND5*, *APRT* and *HPRT* transcript levels analysed by qRT-PCR in WT and *NME6* KO HeLa cells transfected with the indicated esiRNA and incubated with or without nucleosides (100 μM) for 72 h (log2; $n = 3$ independent cultures).

D Representative immunoblot showing the enrichment of mitochondrial proteins in crude mitochondrial fractions taken from the indicated HeLa cell lines during the isolation of mitochondria for LC–MS based metabolomics in Fig 5F. WC, whole cell; CM, crude mitochondria.

E Mitochondrial transcript levels analysed by qRT-PCR in WT and *NME6* KO HeLa cells incubated with the indicated nucleoside species for 48 h as in Fig 5G (A, adenosine; G, guanosine; C, cytidine; U, uridine; log2 transformed; 100 μM; $n = 4$ independent cultures).

F The growth rates of HeLa WT and *NME6* KO cells supplemented with individual nucleosides (A, adenosine; G, guanosine; C, cytidine; U, uridine; log2 transformed; 100 μM; $n = 6$ independent cultures).

G Oxygen consumption rates (OCR) and extracellular acidification rates (ECAR) of WT and *NME6* KO HeLa cells incubated with or without individual nucleosides for a minimum of 120 h. Mitochondrial stress test was performed as in Fig 3A ($n = 3$ independent experiments). The basal and maximal respiration rates shown in Fig 5H were determined from OCR measurements before injection of oligomycin and after injection of FCCP respectively (A, adenosine; G, guanosine; C, cytidine; U, uridine; 100 μM; $n = 3$ independent cultures).

© 2023 The Authors

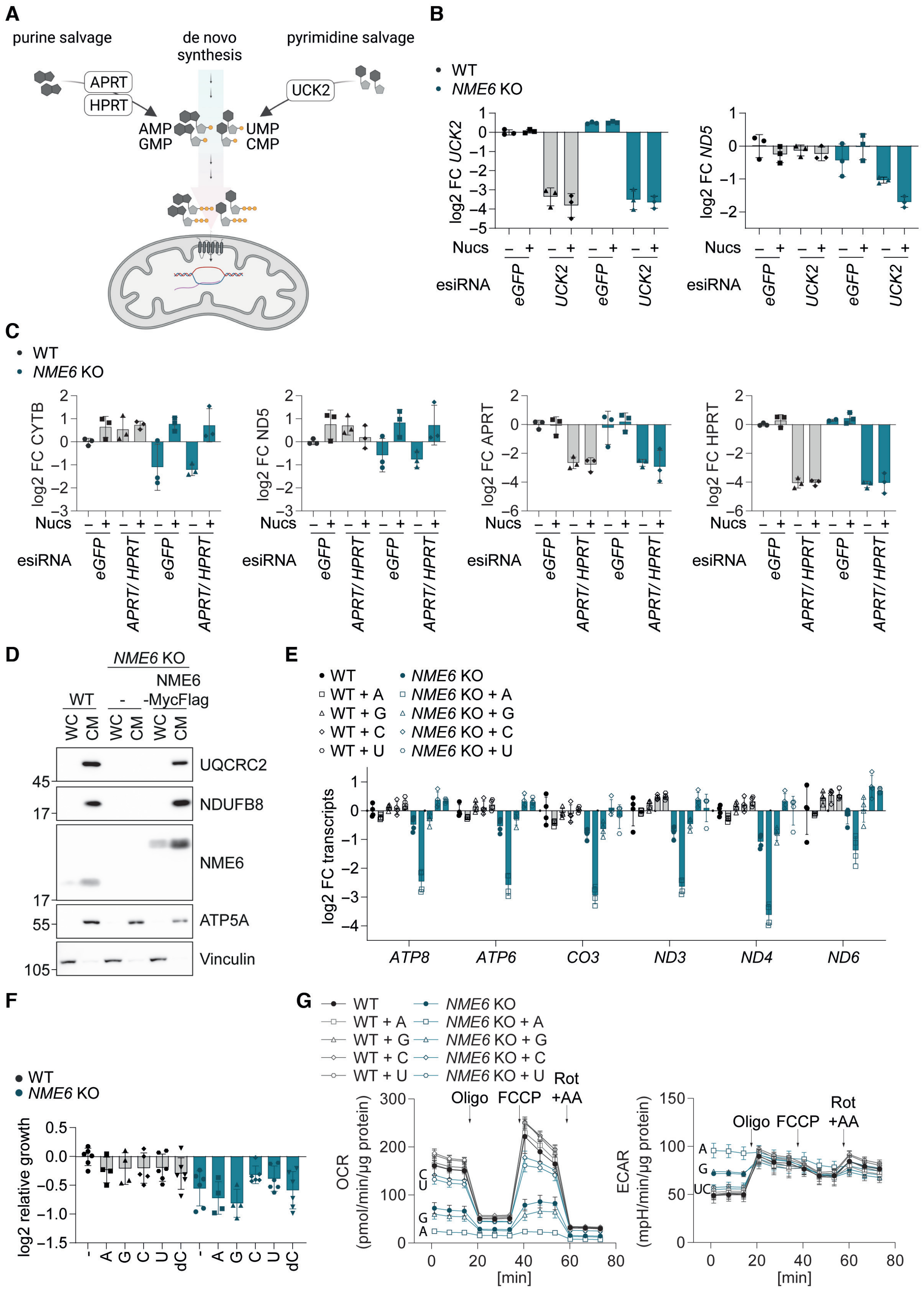
A
purine salvage
de novo synthesis
pyrimidine salvage
APRT
HPRT
UCK2
AMP
GMP
UMP
CMP
B
WT
NME6 KO
log2 FC UCK2
log2 FC ND5
Nucs
esiRNA
eGFP
UCK2
C
log2 FC CYTB
log2 FC ND5
log2 FC APRT
log2 FC HPRT
APRT/ HPRT
D
NME6 KO
WT
-
NME6 -MycFlag
WC
CM
UQCRC2
NDUFB8
NME6
ATP5A
Vinculin
45
17
17
55
105
E
WT + A
WT + G
WT + C
WT + U
NME6 KO + A
NME6 KO + G
NME6 KO + C
NME6 KO + U
log2 FC transcripts
ATP8
ATP6
CO3
ND3
ND4
ND6
F
log2 relative growth
dC
G
OCR (pmol/min/µg protein)
ECAR (mpH/min/µg protein)
Oligo
FCCP
Rot +AA
[min]

Figure EV5.
