# Supplementary figures and images for: Ribonucleotide synthesis by NME6 fuels mitochondrial gene expression
Source: EMBO J. 2023 Jul 13;42(18):e113256. doi: 10.15252/embj.2022113256 (PMC10505918; doi:10.15252/embj.2022113256)

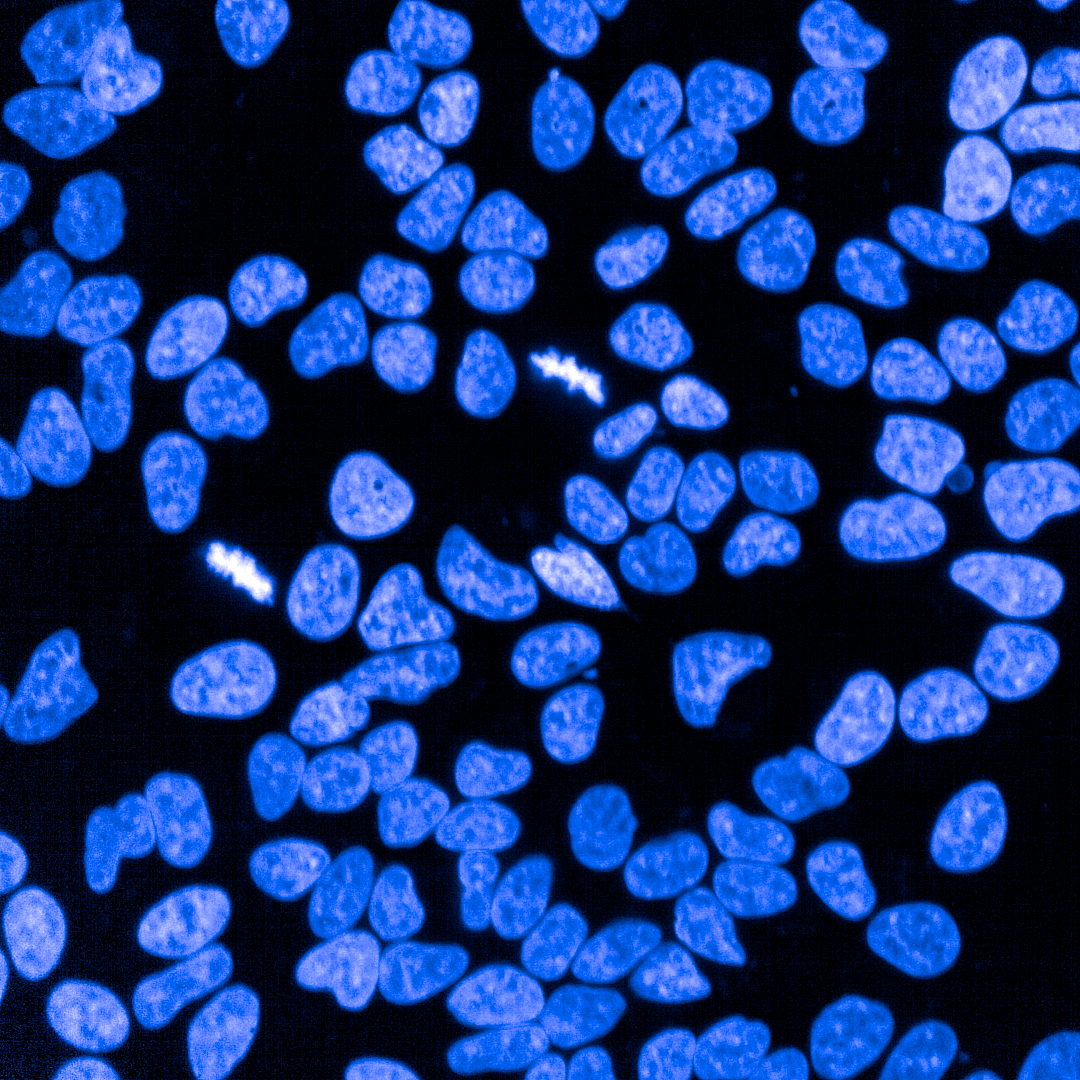

Supplement: Supplementary file 8 — Source Data for Figure 1 [file EMBJ-42-e113256-s002.zip › Fig. 1/Microscopy_1D NTC sgRNA/NTC_405-DAPI_Raw.png]

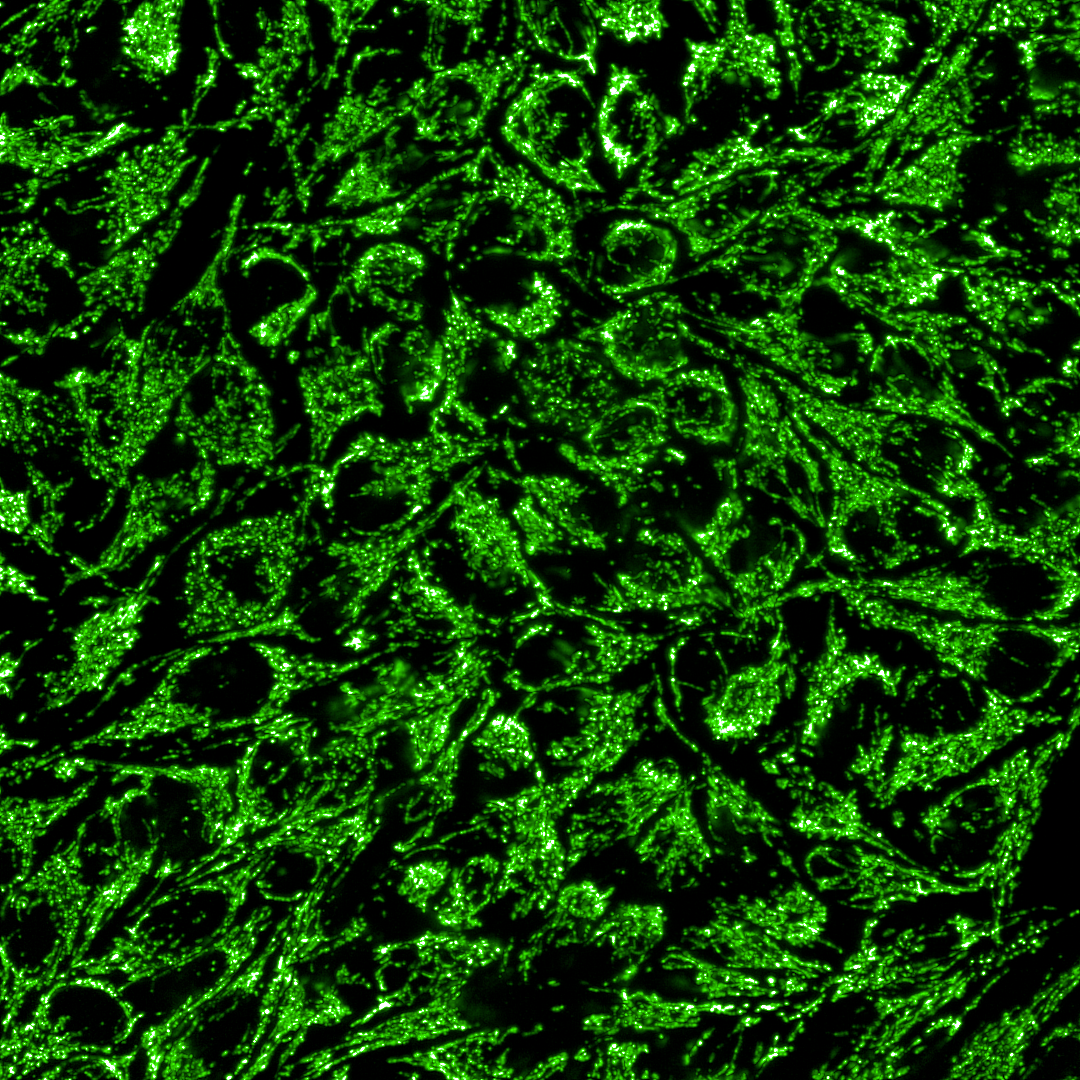

Supplement: Supplementary file 8 — Source Data for Figure 1 [file EMBJ-42-e113256-s002.zip › Fig. 1/Microscopy_1D NTC sgRNA/NTC_488-TFAM_Raw.png]

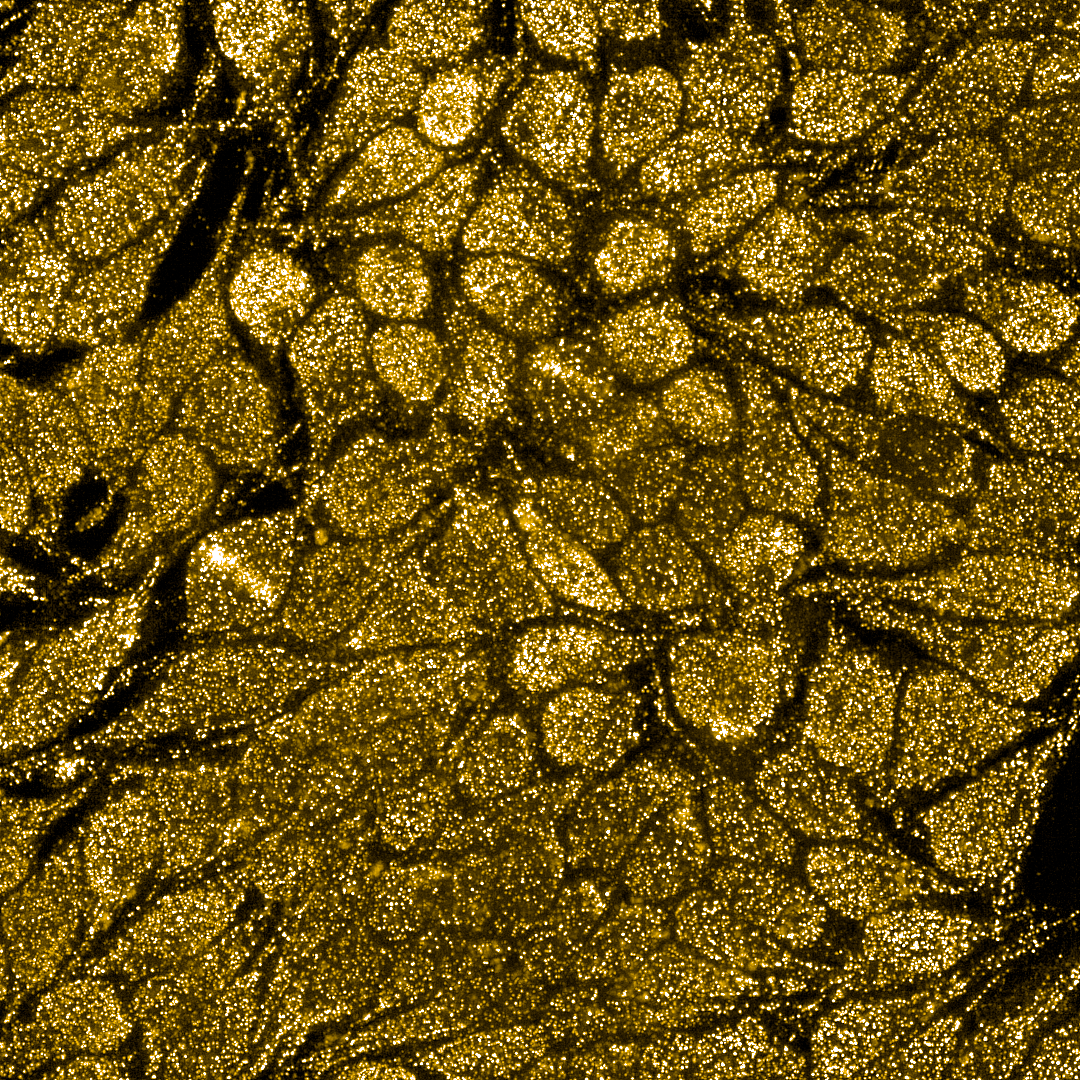

Supplement: Supplementary file 8 — Source Data for Figure 1 [file EMBJ-42-e113256-s002.zip › Fig. 1/Microscopy_1D NTC sgRNA/NTC_561-DNA_Raw.png]

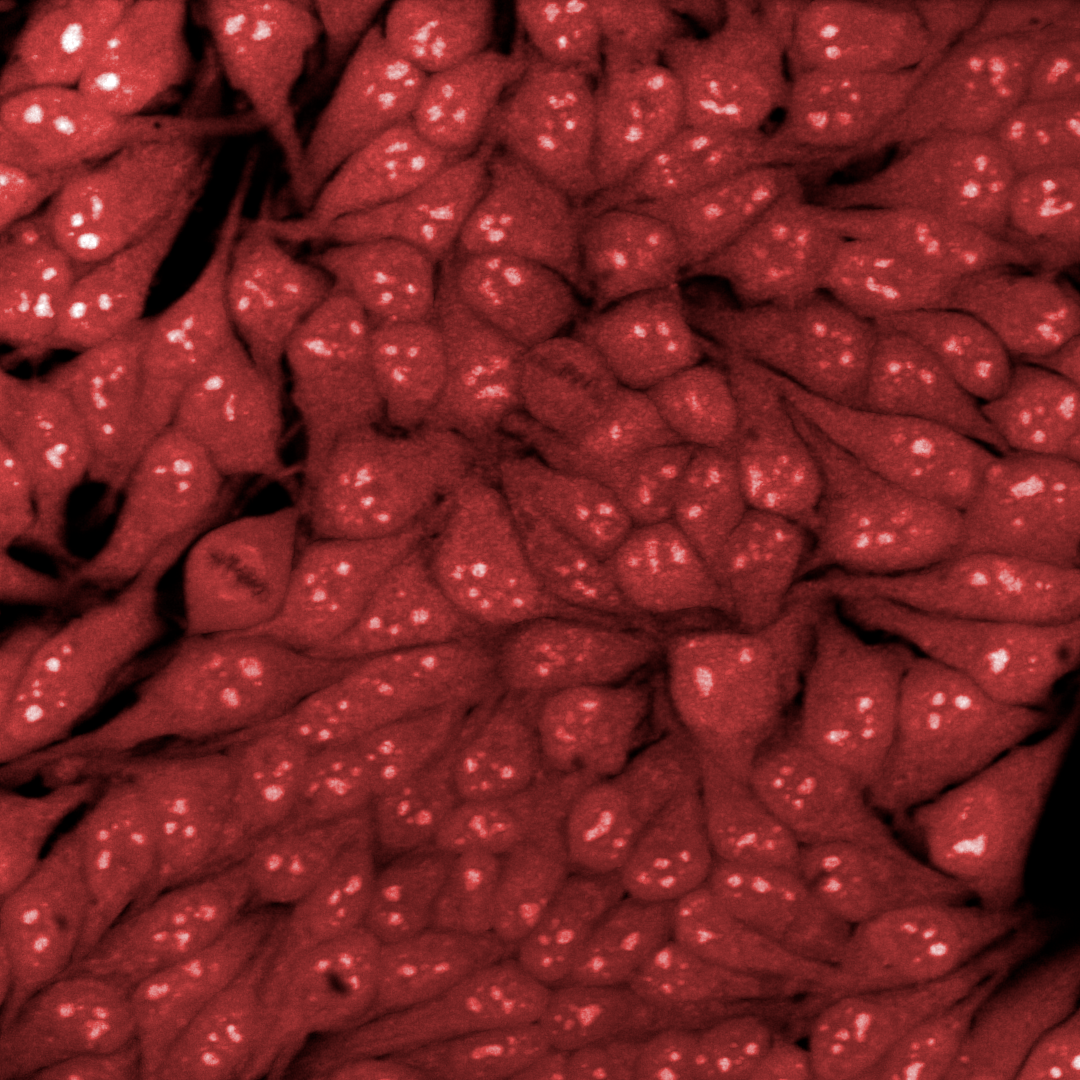

Supplement: Supplementary file 8 — Source Data for Figure 1 [file EMBJ-42-e113256-s002.zip › Fig. 1/Microscopy_1D NTC sgRNA/NTC_640-CellMask_Raw.png]

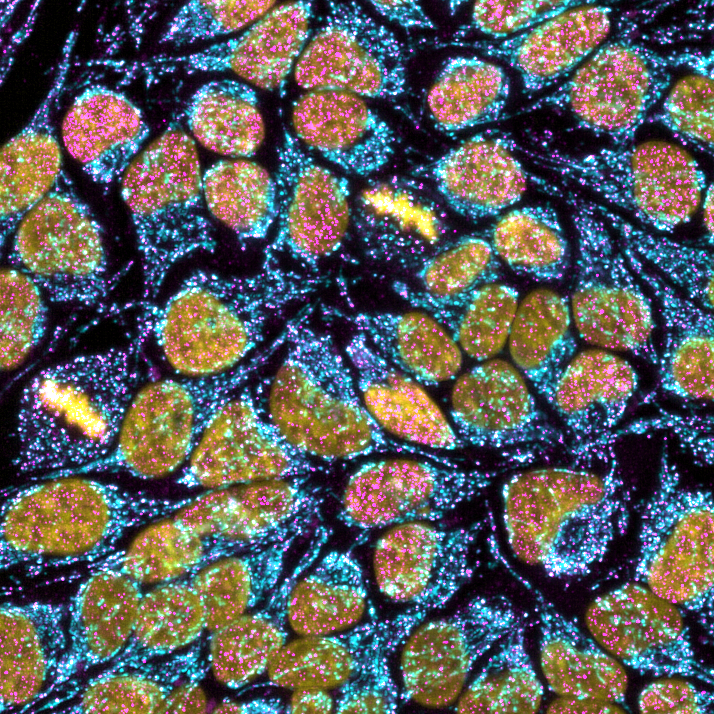

Supplement: Supplementary file 8 — Source Data for Figure 1 [file EMBJ-42-e113256-s002.zip › Fig. 1/Microscopy_1D NTC sgRNA/NTC_Cropped_Composite_Pseudocolour.png]

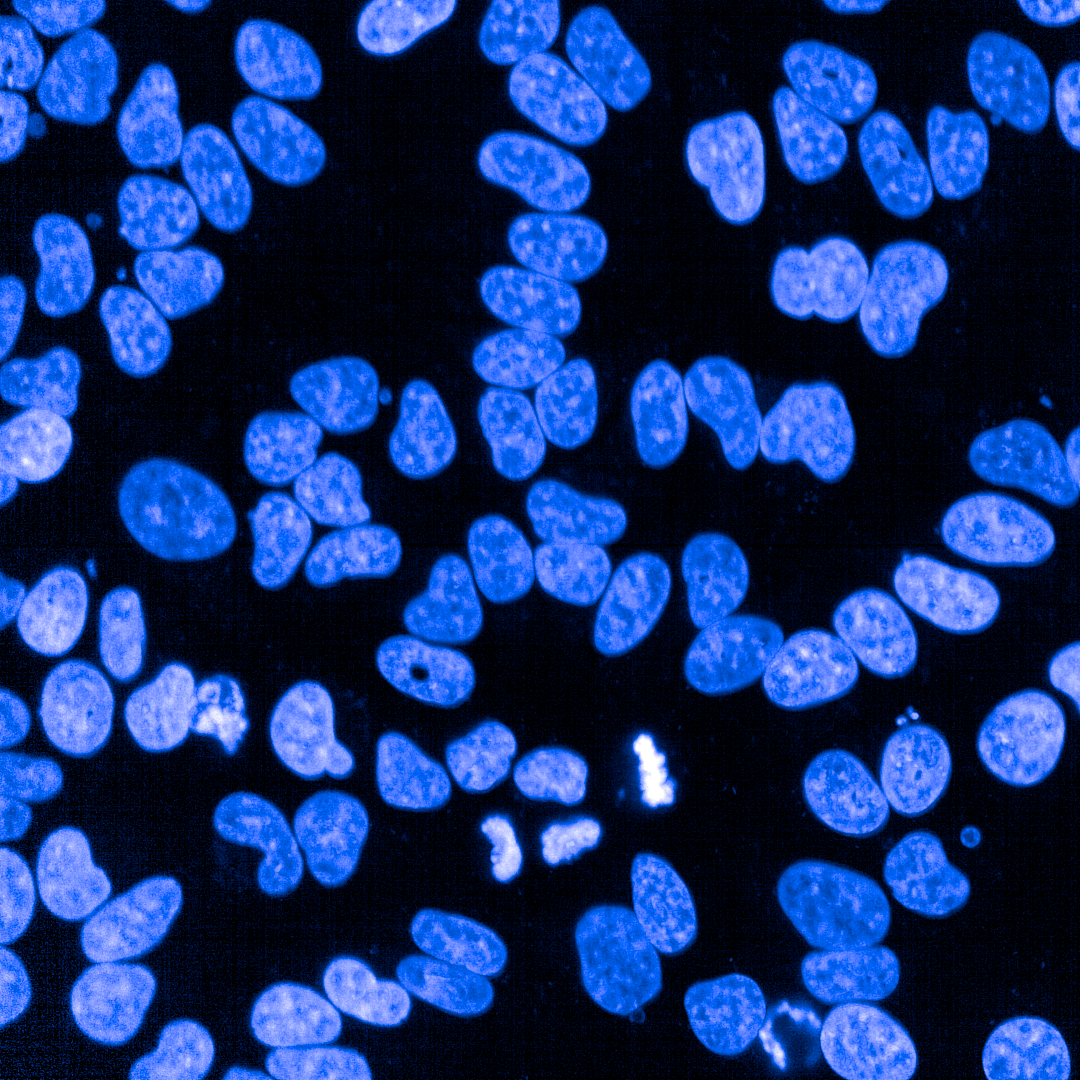

Supplement: Supplementary file 8 — Source Data for Figure 1 [file EMBJ-42-e113256-s002.zip › Fig. 1/Microscopy_1D TFAM sgRNA/TFAM_405-DAPI_Raw.png]

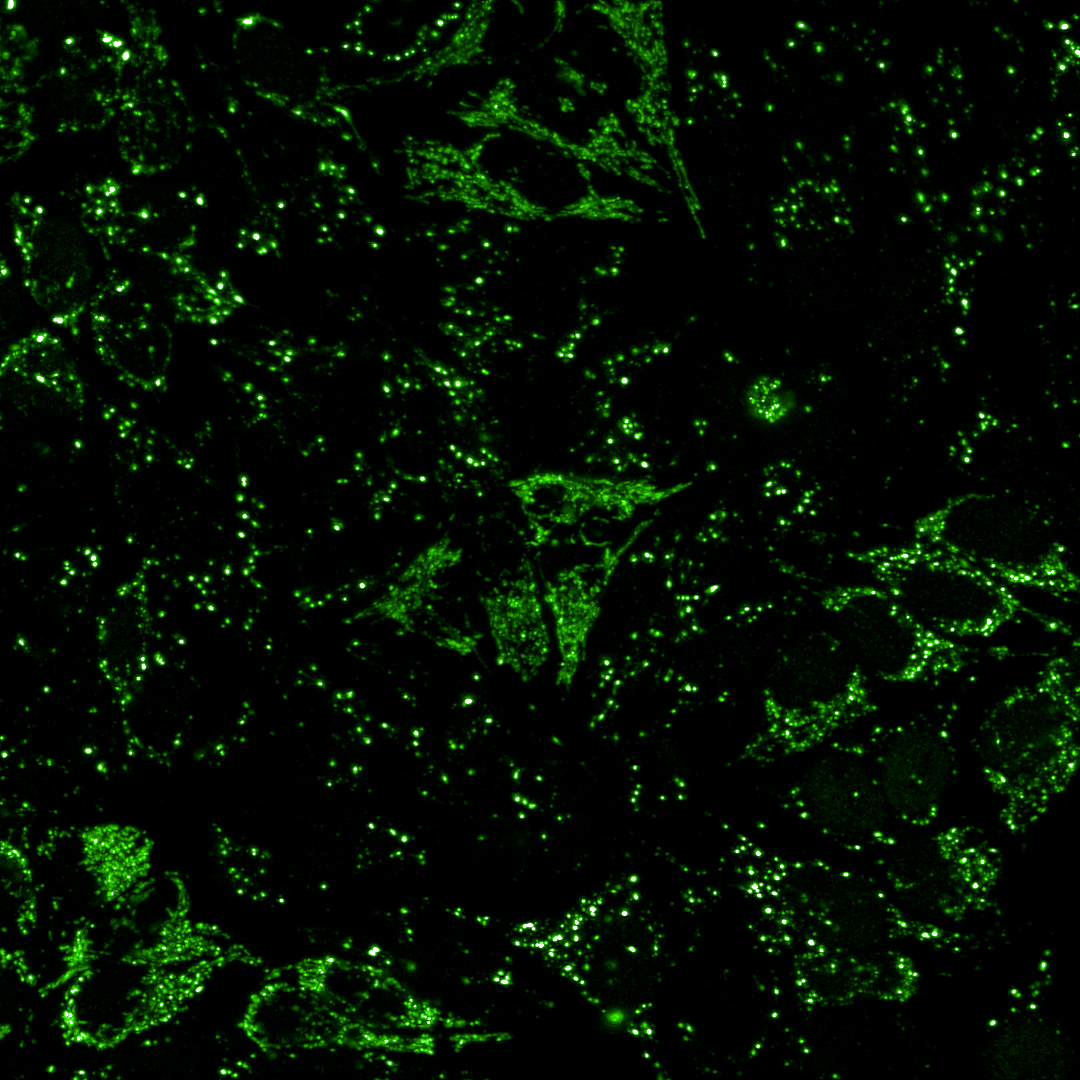

Supplement: Supplementary file 8 — Source Data for Figure 1 [file EMBJ-42-e113256-s002.zip › Fig. 1/Microscopy_1D TFAM sgRNA/TFAM_488-TFAM_Raw.png]

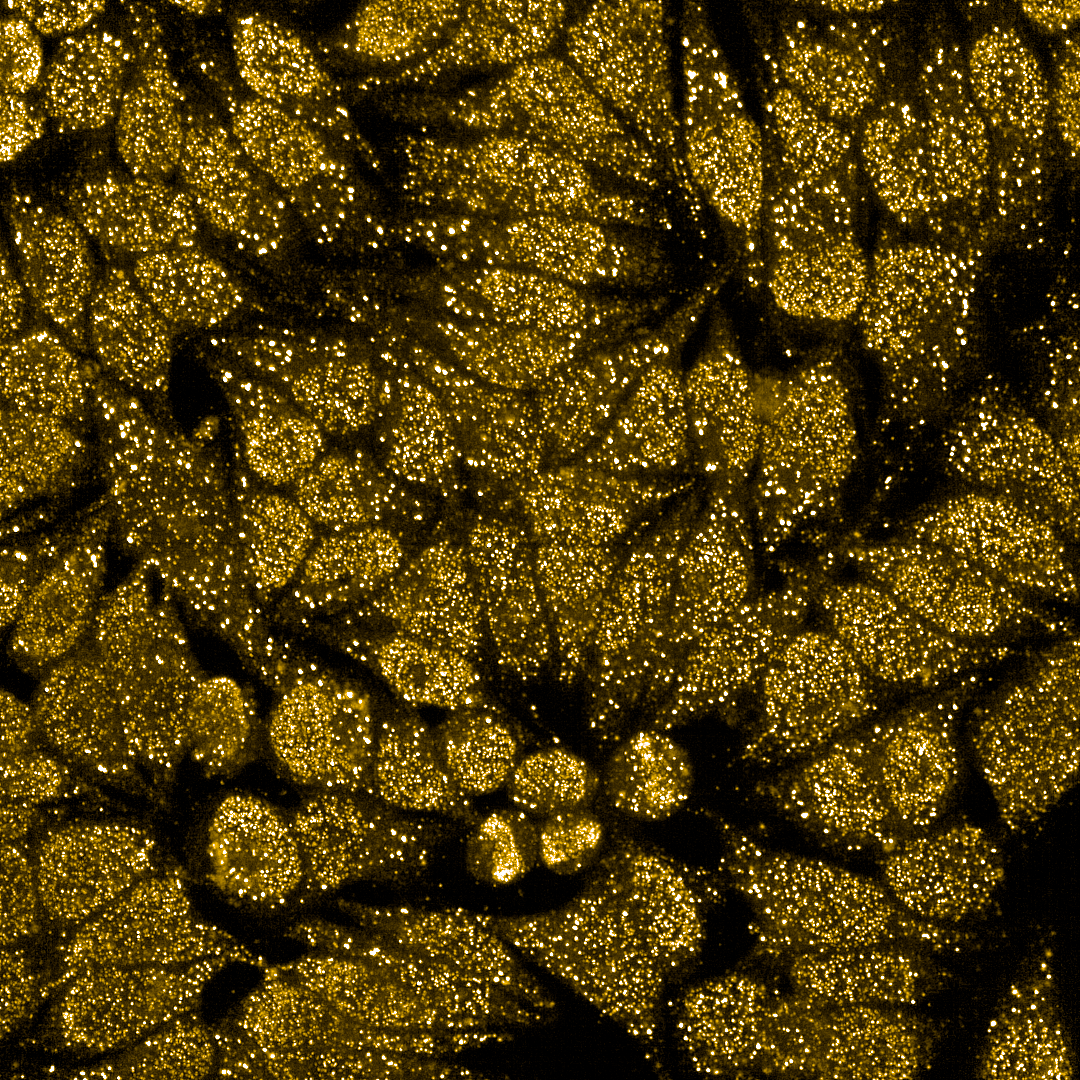

Supplement: Supplementary file 8 — Source Data for Figure 1 [file EMBJ-42-e113256-s002.zip › Fig. 1/Microscopy_1D TFAM sgRNA/TFAM_561-DNA_Raw.png]

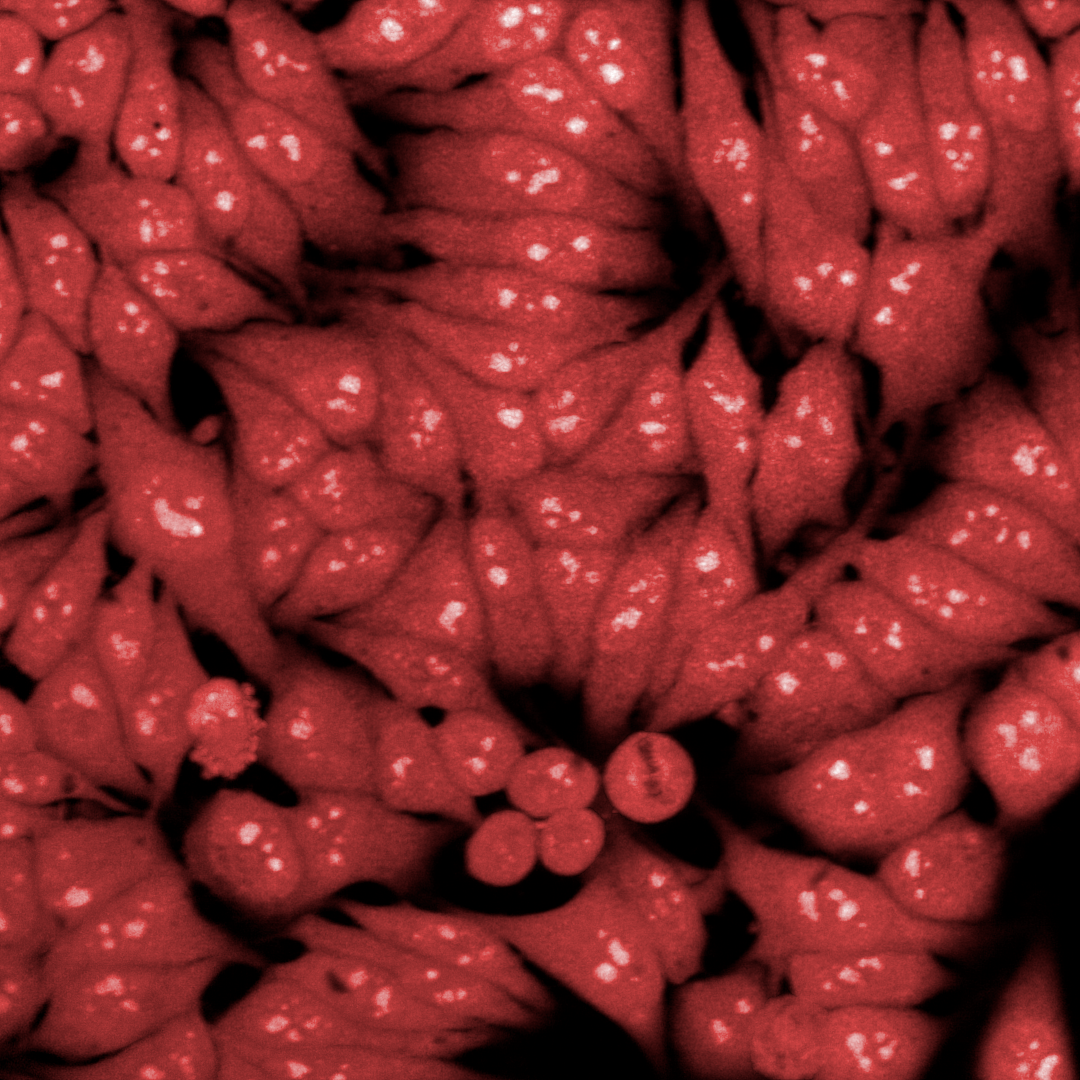

Supplement: Supplementary file 8 — Source Data for Figure 1 [file EMBJ-42-e113256-s002.zip › Fig. 1/Microscopy_1D TFAM sgRNA/TFAM_640-CellMask_Raw.png]

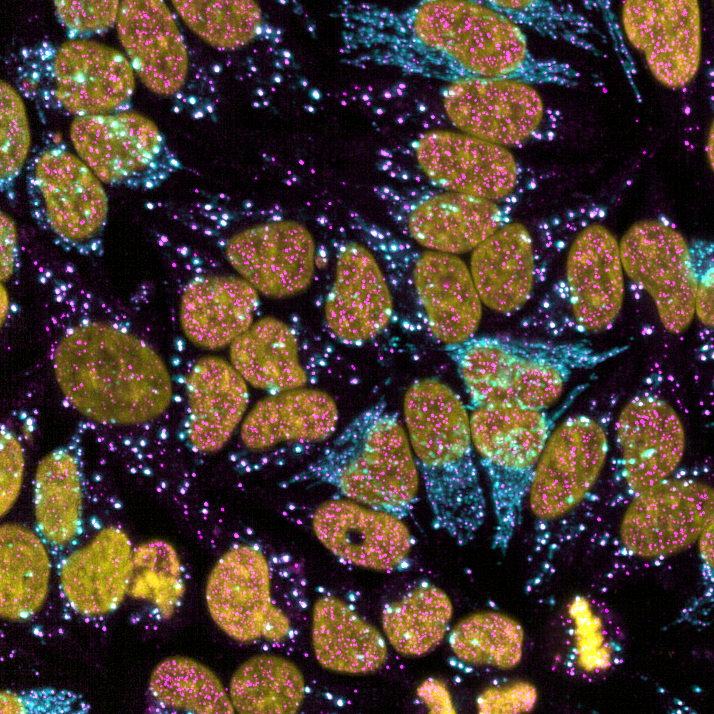

Supplement: Supplementary file 8 — Source Data for Figure 1 [file EMBJ-42-e113256-s002.zip › Fig. 1/Microscopy_1D TFAM sgRNA/TFAM_Cropped_Composite.png]

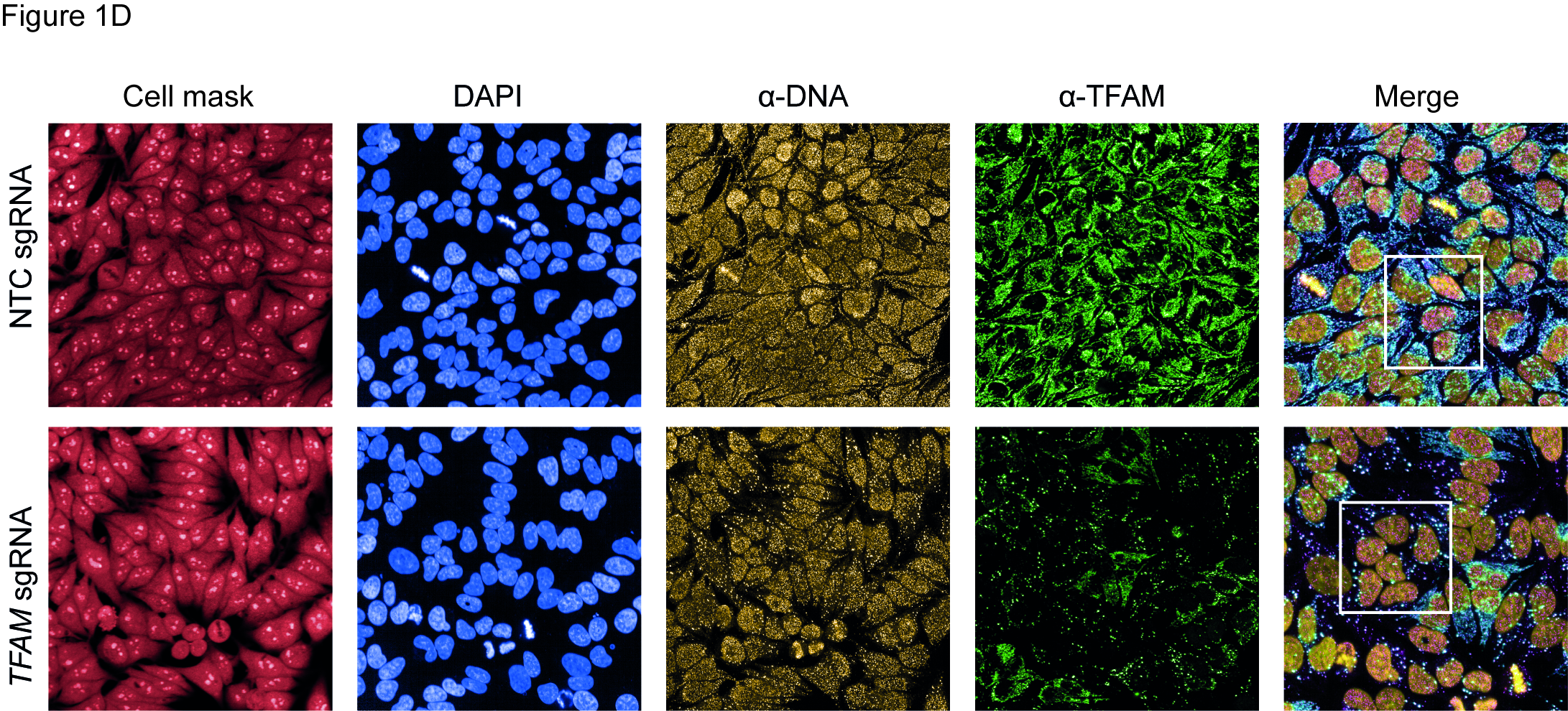

Supplement: Supplementary file 8 — Source Data for Figure 1 [file EMBJ-42-e113256-s002.zip › Fig. 1/Microscopy_1D.tif]

1B

WT  
A33 KO  
A33/36 DKO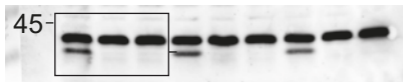

SLC25A33

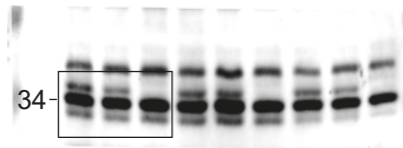

SLC25A36

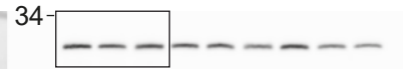

MT-CO2

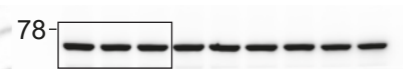

SDHA

Supplement: Supplementary file 8 — Source Data for Figure 1 [file EMBJ-42-e113256-s002.zip › Fig. 1/WB_Fig1B.pdf]

1H

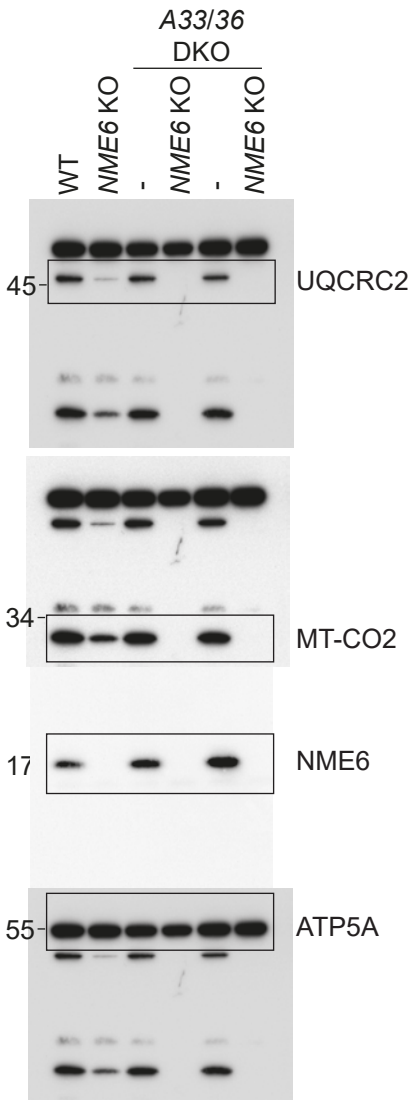

Supplement: Supplementary file 8 — Source Data for Figure 1 [file EMBJ-42-e113256-s002.zip › Fig. 1/WB_Fig1H.pdf]

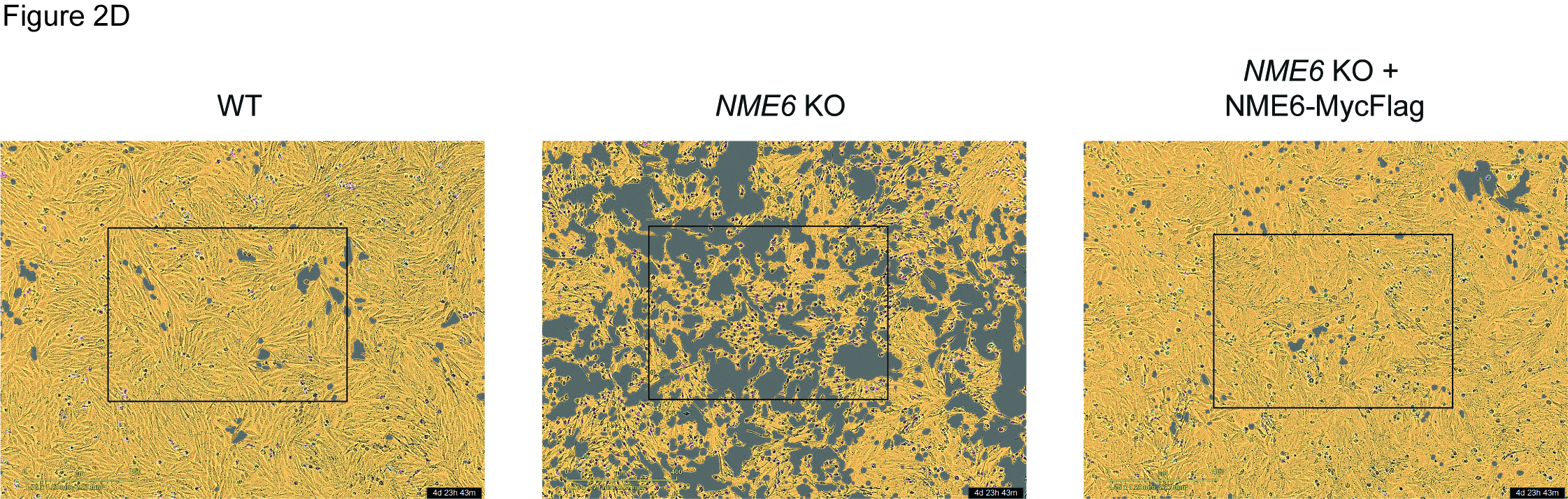

Supplement: Supplementary file 9 — Source Data for Figure 2 [file EMBJ-42-e113256-s001.zip › Fig. 2/Incucyte_2D.tif]

3E

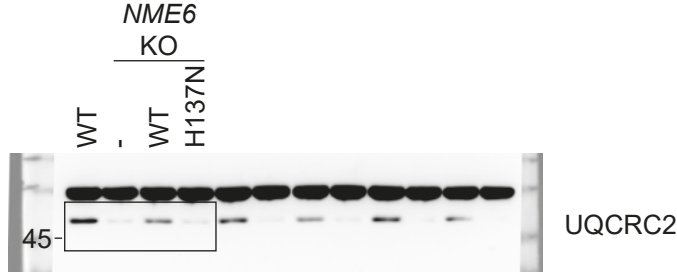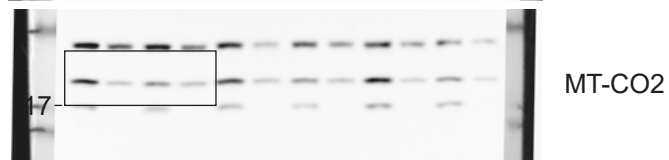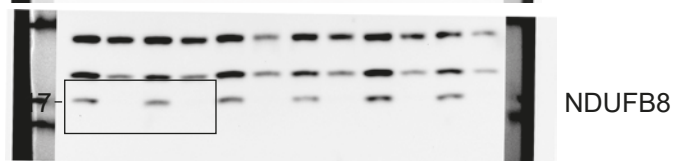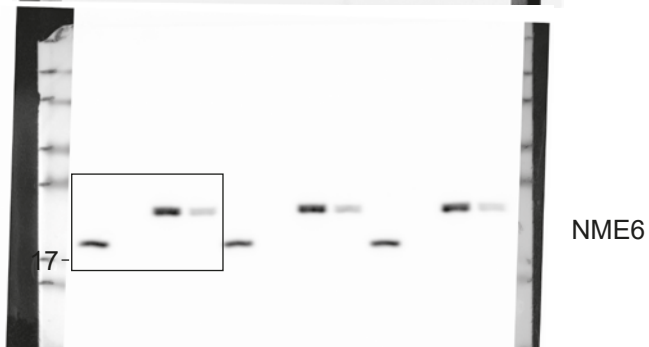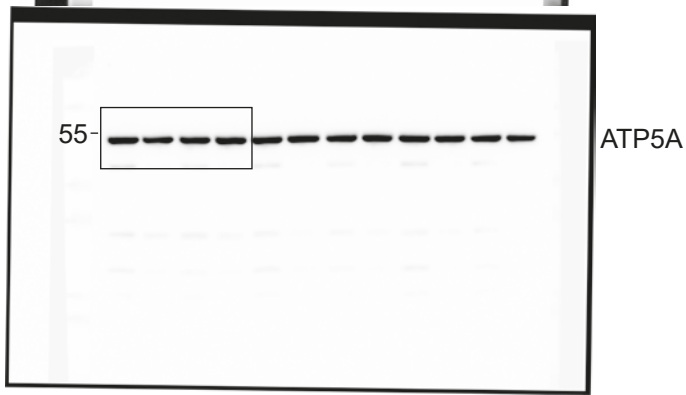

Supplement: Supplementary file 10 — Source Data for Figure 3 [file EMBJ-42-e113256-s009.zip › Fig. 3/WB_Fig3E.pdf]

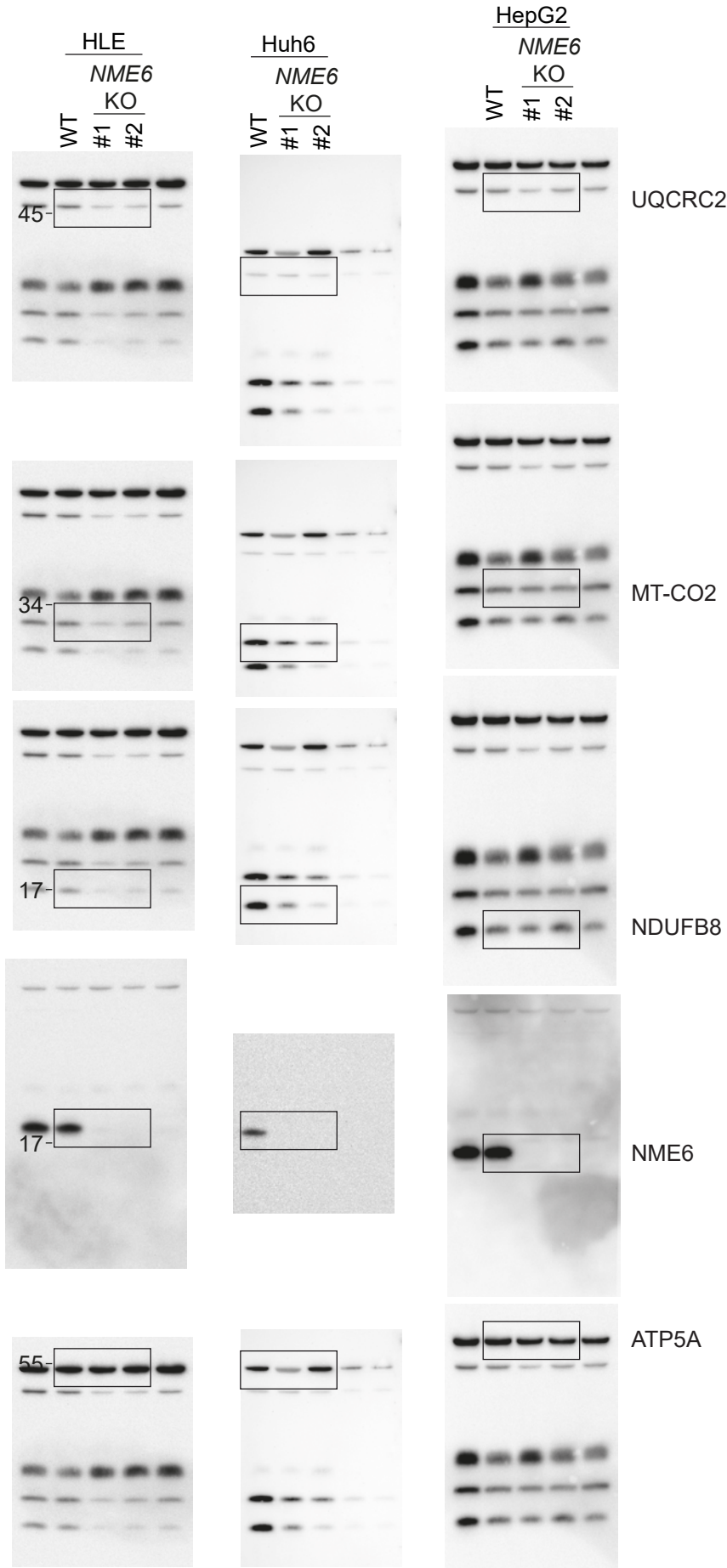

Supplement: Supplementary file 10 — Source Data for Figure 3 [file EMBJ-42-e113256-s009.zip › Fig. 3/WB_Fig3F.pdf]

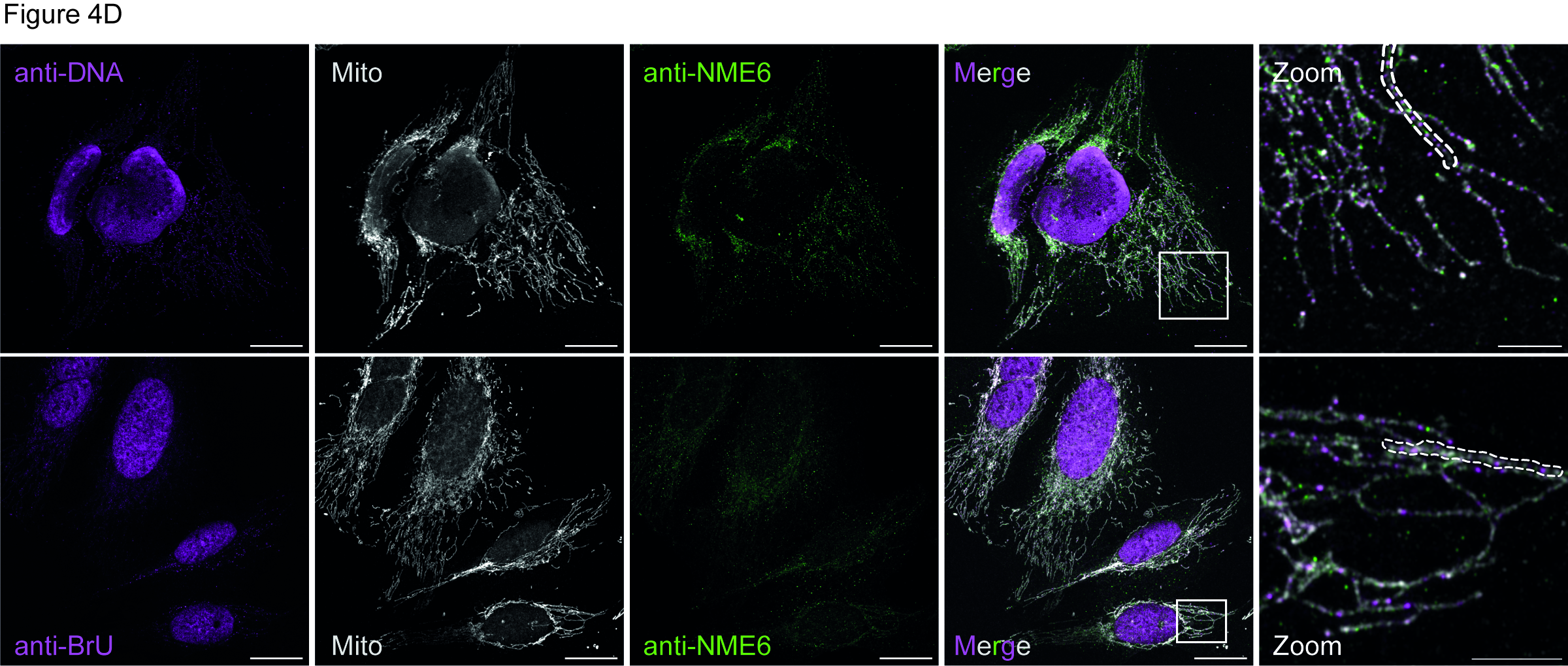

Supplement: Supplementary file 11 — Source Data for Figure 4 [file EMBJ-42-e113256-s012.zip › Fig. 4/Microscopy_4C.tif]

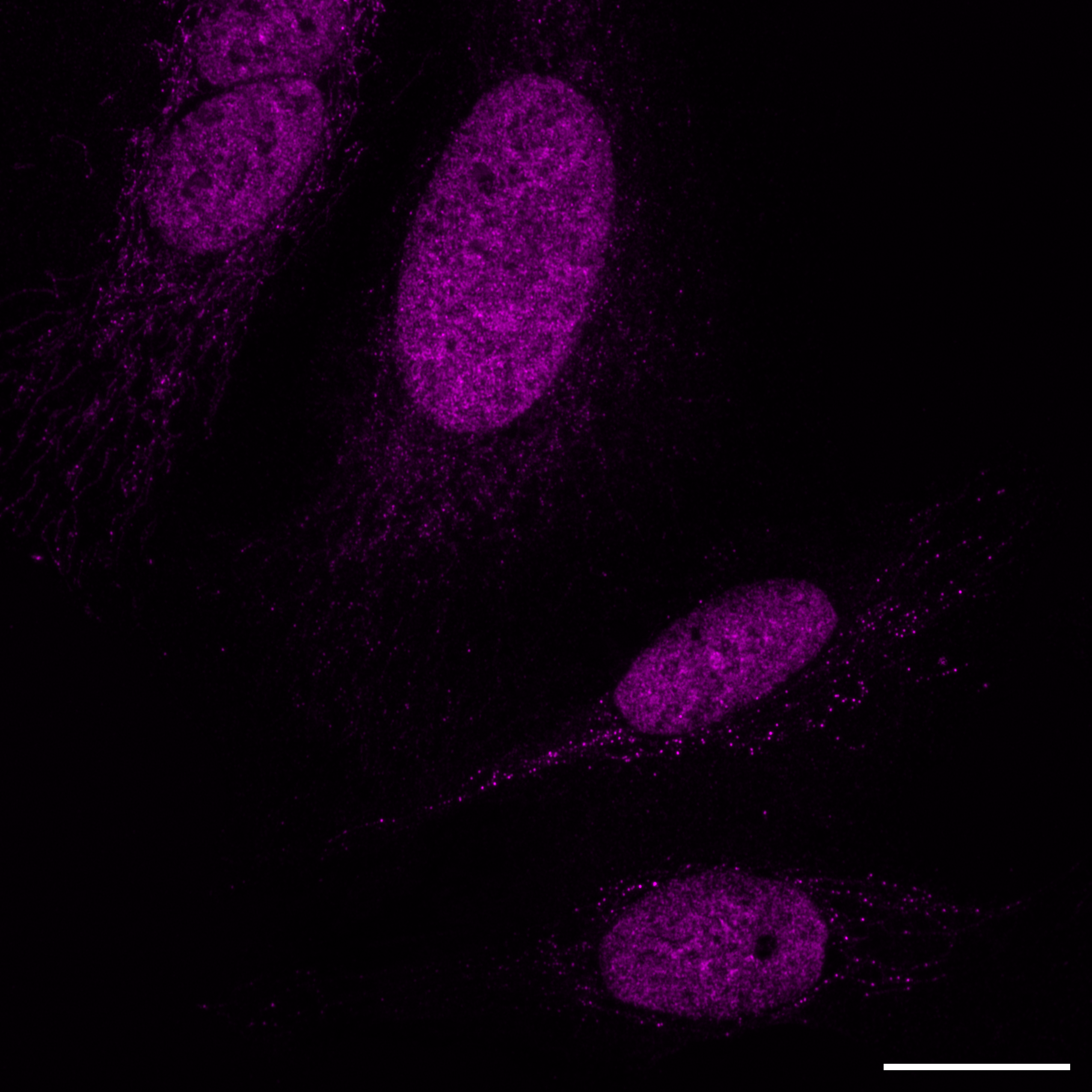

Supplement: Supplementary file 11 — Source Data for Figure 4 [file EMBJ-42-e113256-s012.zip › Fig. 4/Microscopy_4C_BrU/NME6_BrU_full cell_BrU_Channel 2.png]

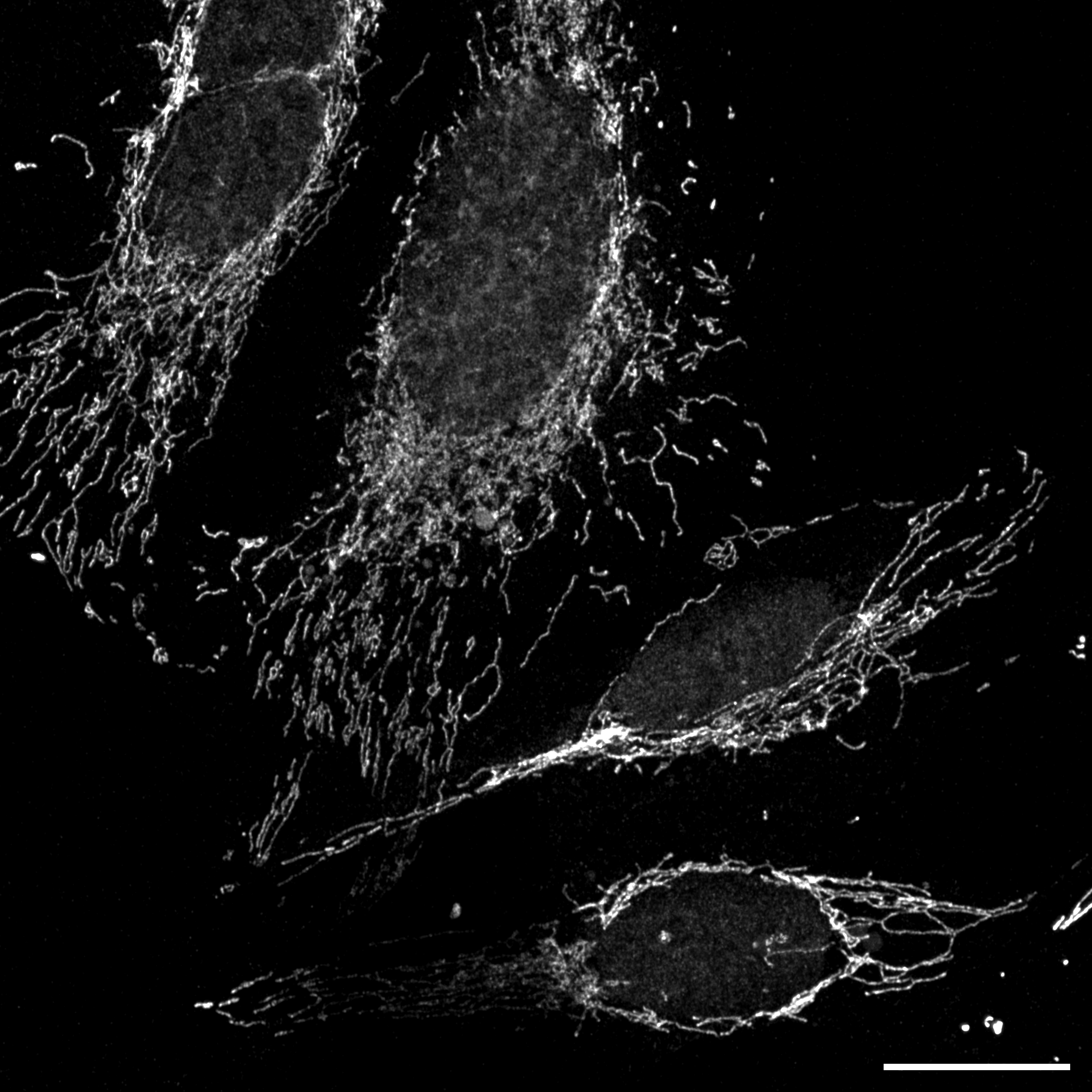

Supplement: Supplementary file 11 — Source Data for Figure 4 [file EMBJ-42-e113256-s012.zip › Fig. 4/Microscopy_4C_BrU/NME6_BrU_full cell_mito_Channel 1.png]

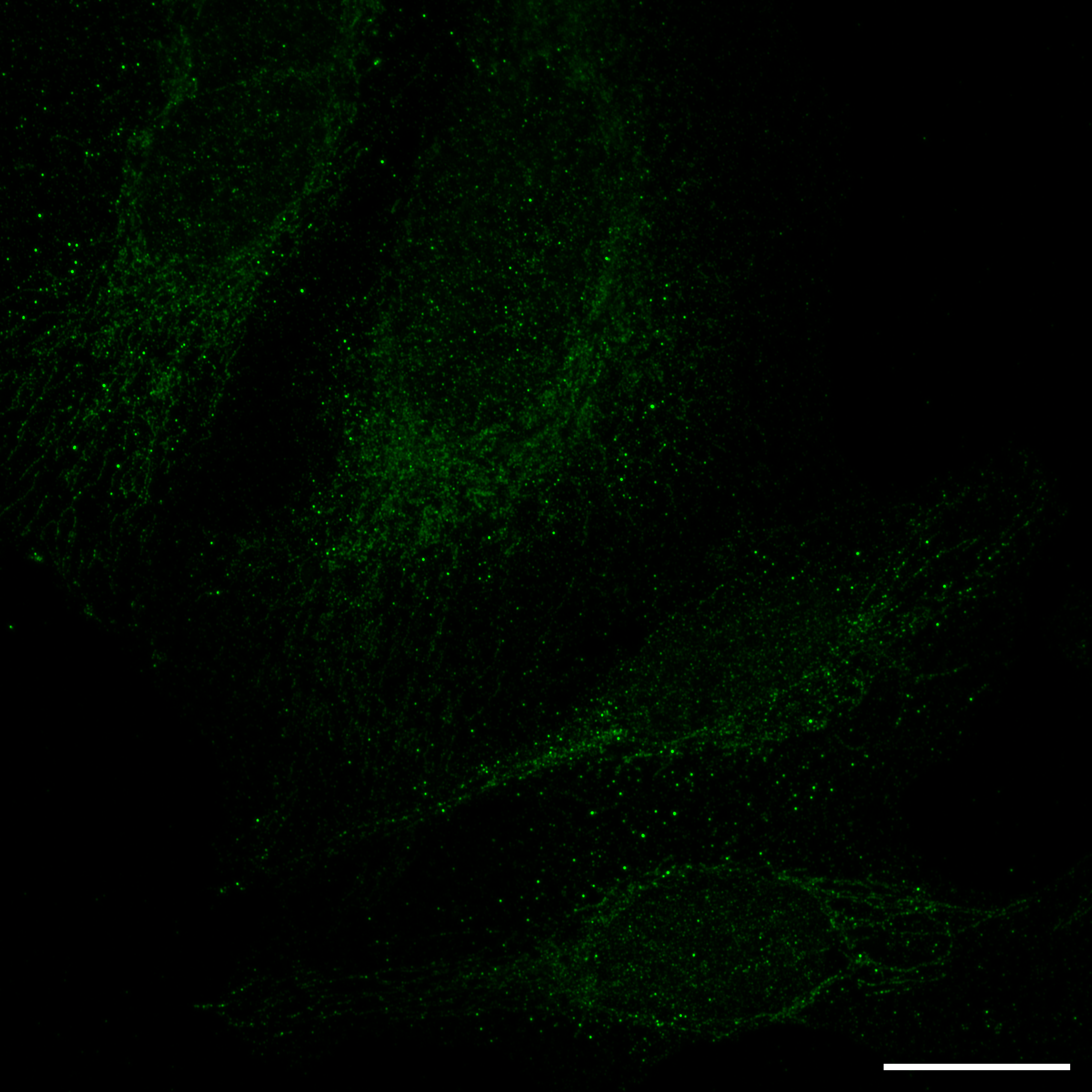

Supplement: Supplementary file 11 — Source Data for Figure 4 [file EMBJ-42-e113256-s012.zip › Fig. 4/Microscopy_4C_BrU/NME6_BrU_full cell_NME6_Channel 3.png]

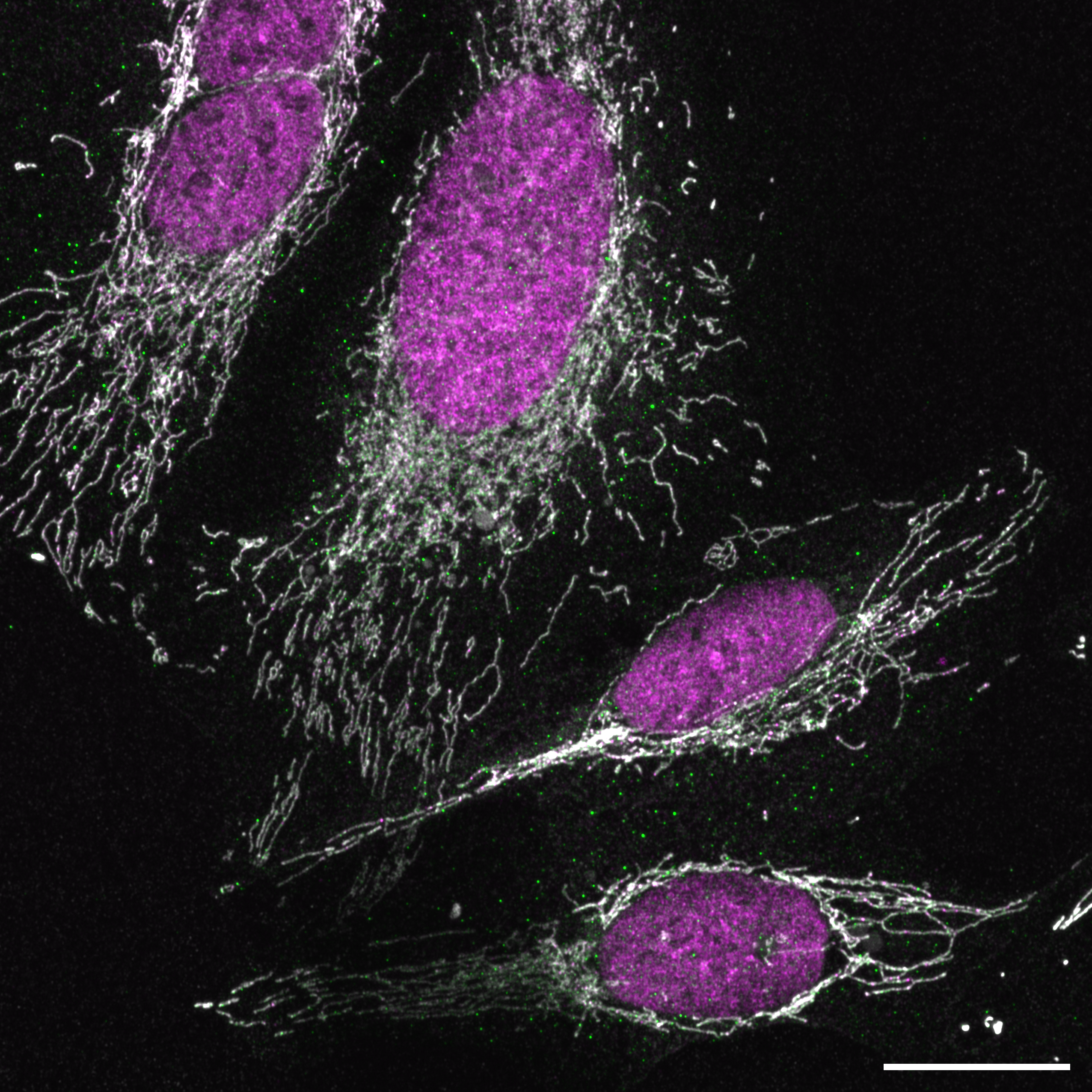

Supplement: Supplementary file 11 — Source Data for Figure 4 [file EMBJ-42-e113256-s012.zip › Fig. 4/Microscopy_4C_BrU/NME6_BrU_full cell_scale bar 20um_TM.png]

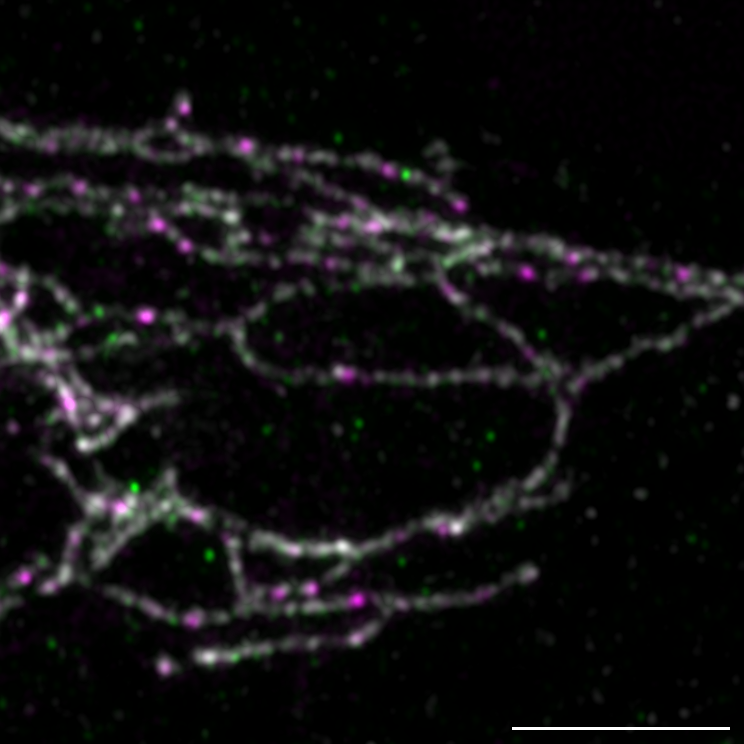

Supplement: Supplementary file 11 — Source Data for Figure 4 [file EMBJ-42-e113256-s012.zip › Fig. 4/Microscopy_4C_BrU/NME6_BrU_zoom_NME6 & BrU & mito.png]

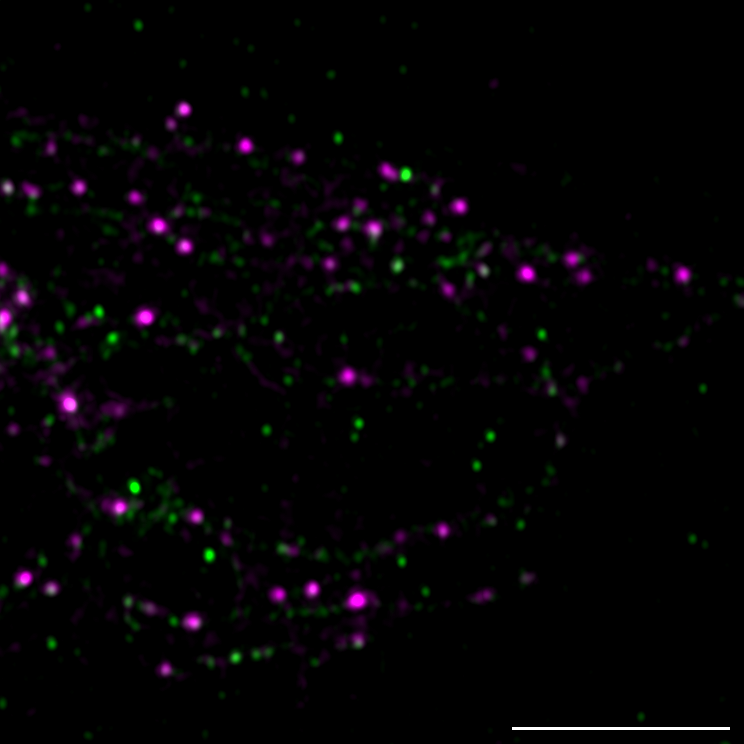

Supplement: Supplementary file 11 — Source Data for Figure 4 [file EMBJ-42-e113256-s012.zip › Fig. 4/Microscopy_4C_BrU/NME6_BrU_zoom_NME6 & BrU_TM.png]

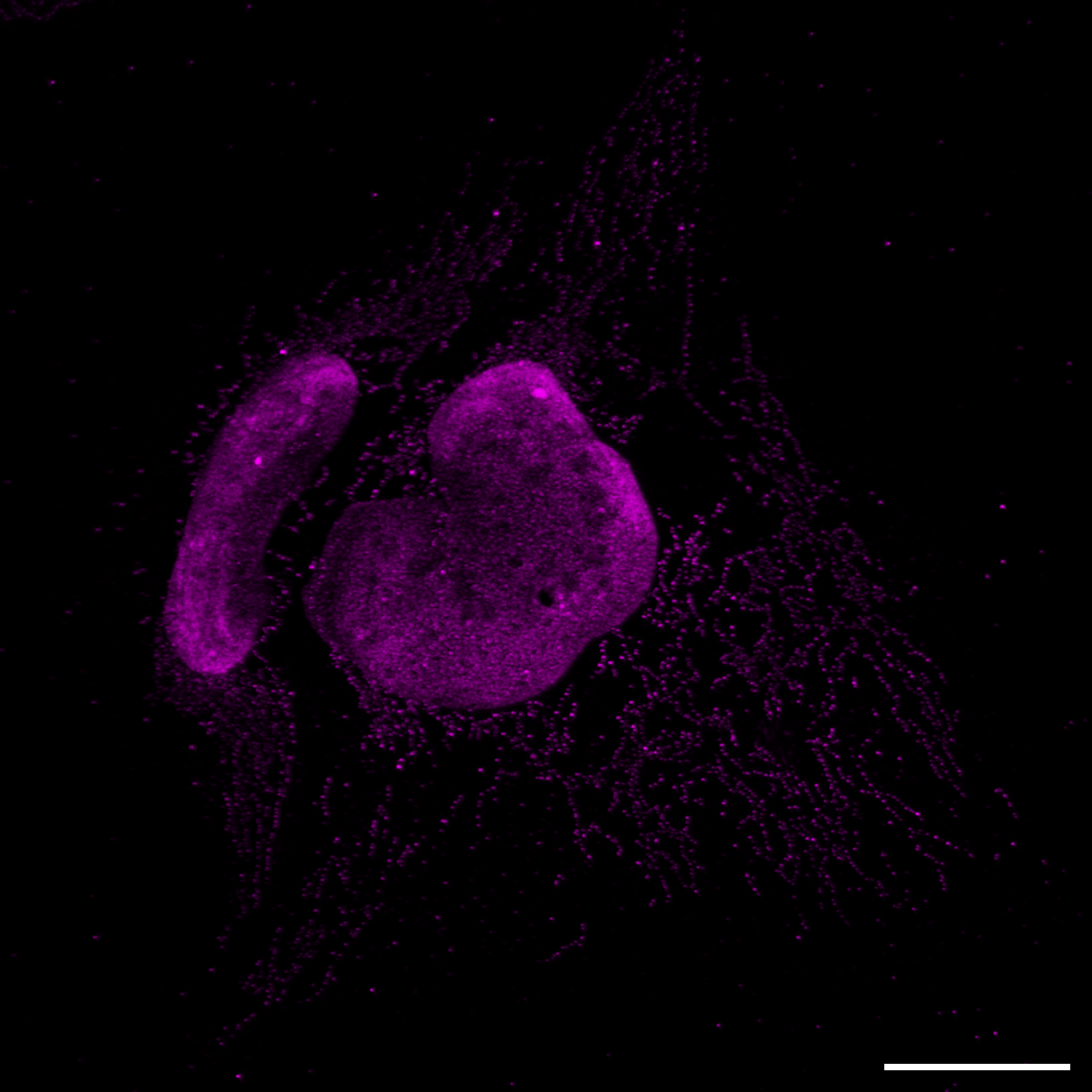

Supplement: Supplementary file 11 — Source Data for Figure 4 [file EMBJ-42-e113256-s012.zip › Fig. 4/Microscopy_4C_DNA/NME6_DNA_full cell_DNA_Channel 2.png]

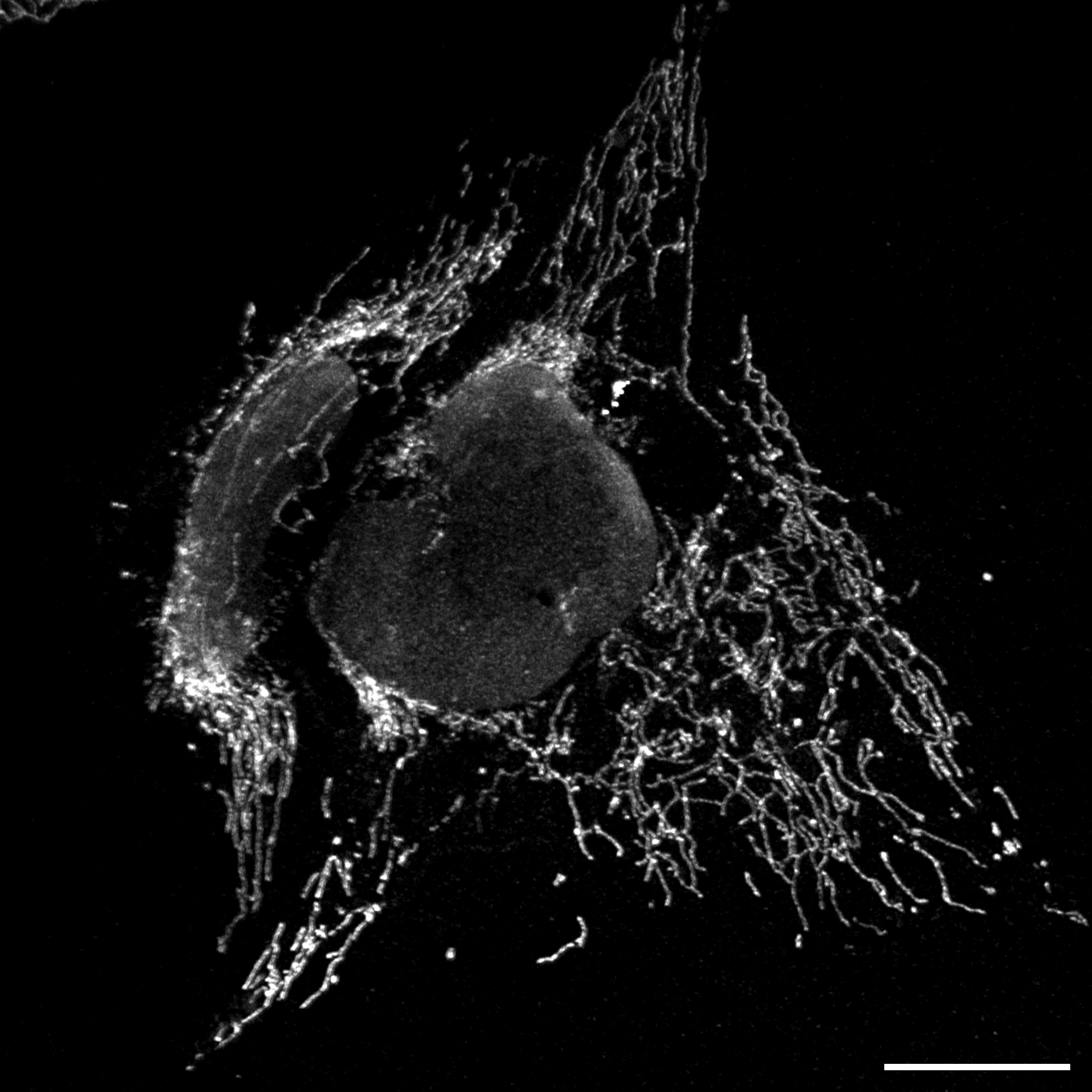

Supplement: Supplementary file 11 — Source Data for Figure 4 [file EMBJ-42-e113256-s012.zip › Fig. 4/Microscopy_4C_DNA/NME6_DNA_full cell_mito_Channel 1.png]

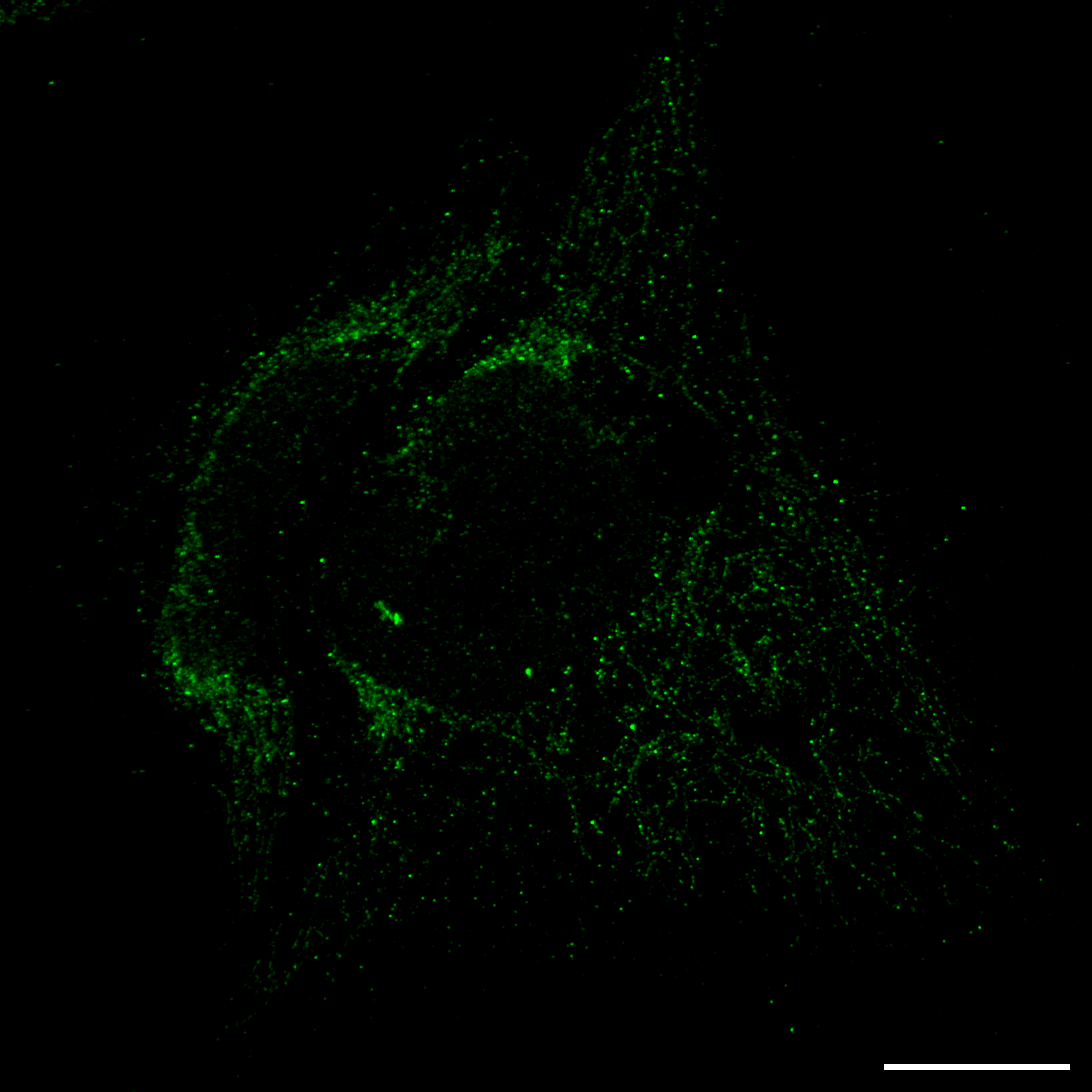

Supplement: Supplementary file 11 — Source Data for Figure 4 [file EMBJ-42-e113256-s012.zip › Fig. 4/Microscopy_4C_DNA/NME6_DNA_full cell_NME6_Channel 3.png]

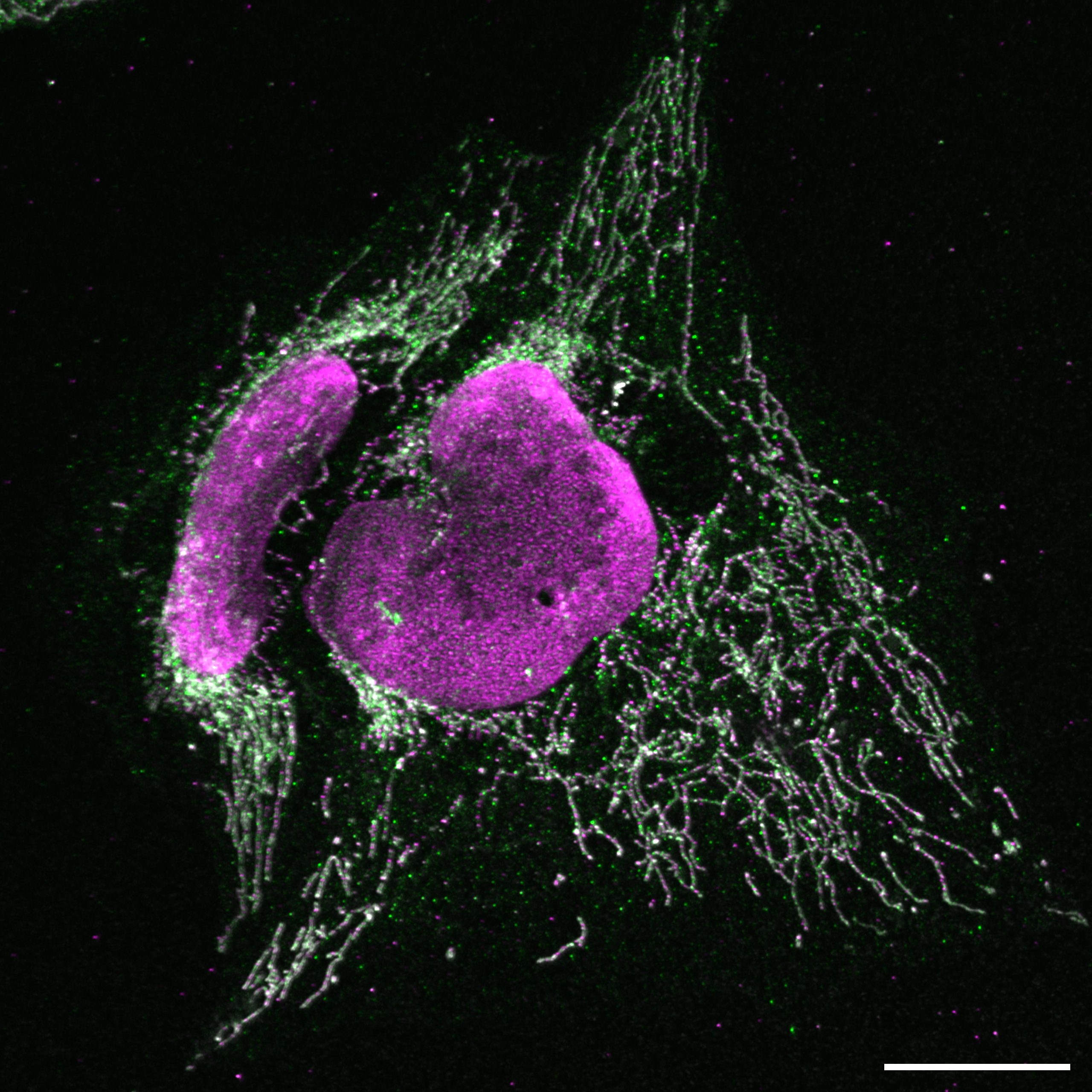

Supplement: Supplementary file 11 — Source Data for Figure 4 [file EMBJ-42-e113256-s012.zip › Fig. 4/Microscopy_4C_DNA/NME6_DNA_full cell_scale bar 20um_COmposite.png]

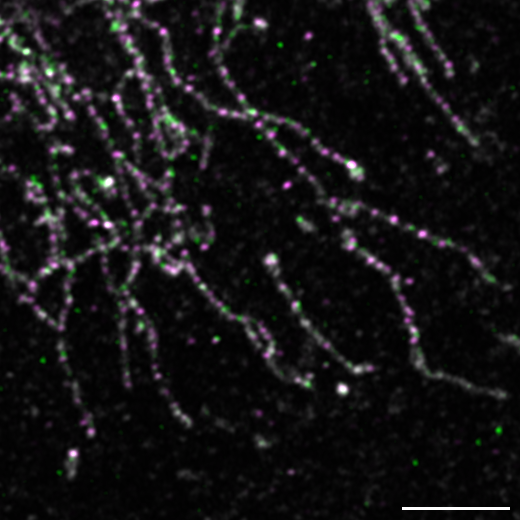

Supplement: Supplementary file 11 — Source Data for Figure 4 [file EMBJ-42-e113256-s012.zip › Fig. 4/Microscopy_4C_DNA/NME6_DNA_zoom_NME6 & DNA & mito.png]

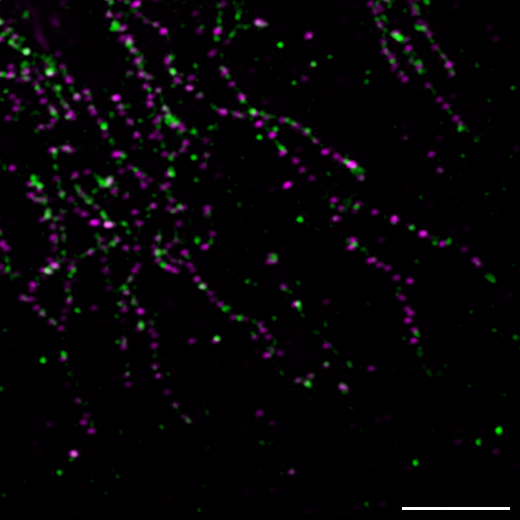

Supplement: Supplementary file 11 — Source Data for Figure 4 [file EMBJ-42-e113256-s012.zip › Fig. 4/Microscopy_4C_DNA/NME6_DNA_zoom_NME6 & DNA.png]

4A

*NME6* KO  
+ *NME6*  
-MycFlag

|       | WT |    |    |    | <i>NME6</i> KO |    |    |    | <i>NME6</i> KO + <i>NME6</i> -MycFlag |    |    |    |
|-------|----|----|----|----|----------------|----|----|----|---------------------------------------|----|----|----|
| [min] | 15 | 30 | 45 | 60 | 15             | 30 | 45 | 60 | 15                                    | 30 | 45 | 60 |

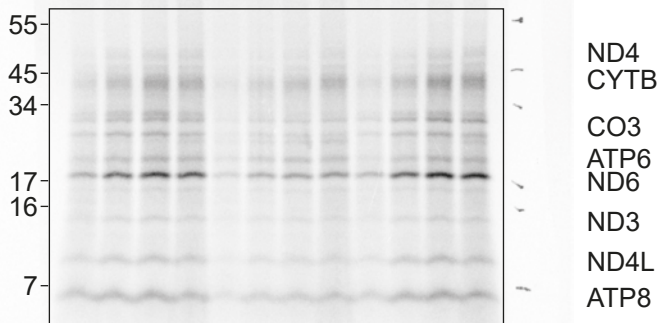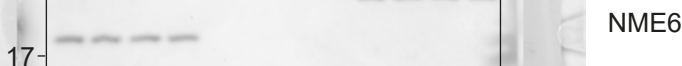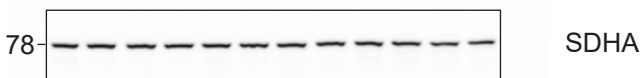

Supplement: Supplementary file 11 — Source Data for Figure 4 [file EMBJ-42-e113256-s012.zip › Fig. 4/WB_Fig4A.pdf]
