# Supplementary material for: Ribonucleotide synthesis by NME6 fuels mitochondrial gene expression
Source: EMBO J. 2023 Jul 13;42(18):e113256. doi: 10.15252/embj.2022113256 (PMC10505918; doi:10.15252/embj.2022113256)
Supplement: Supplementary file 13 [file EMBJ-42-e113256-s011.pdf]

THE EMBO JOURNAL

Ribonucleotide synthesis by NME6 fuels mitochondrial gene expression

Nils Grotehans, Lynn McGarry, Hendrik Nolte, Vanessa Xavier, Moritz Kroker, Álvaro Jesús Narbona-Pérez, Soni Deshwal, Patrick Giavalisco, Thomas Langer and Thomas MacVicar
DOI: 10.15252/embj.2022113256

Corresponding author(s): Thomas MacVicar (T.MacVicar@beatson.gla.ac.uk) , Thomas Langer (Langer@age.mpg.de)

Review Timeline:

Submission Date:	11th Dec 22
Editorial Decision:	31st Jan 23
Revision Received:	28th Apr 23
Editorial Decision:	26th May 23
Revision Received:	7th Jun 23
Accepted:	19th Jun 23

Editor: Stefanie Boehm / William Teale

Transaction Report:

(Note: With the exception of the correction of typographical or spelling errors that could be a source of ambiguity, letters and reports are not edited. Depending on transfer agreements, referee reports obtained elsewhere may or may not be included in this compilation. Referee reports are anonymous unless the Referee chooses to sign their reports.)

Dr. Thomas MacVicar
CRUK Beatson Institute
CRUK Beatson Institute, Switchback Road
Bearsden
Glasgow, Glasgow G61 1BD
United Kingdom

31st Jan 2023

Re: EMBOJ-2022-113256
Ribonucleotide synthesis by NME6 fuels mitochondrial gene expression

Dear Dr. MacVicar,

Thank you for submitting your manuscript reporting a role NME6 in mitochondrial gene expression to The EMBO Journal. We have now received feedback on your study from three experts in the field. I have discussed these reports with the editorial team, as well as again contacting the referees for cross-commenting on the reports. Taking everything together, we would like to invite you to prepare and submit a revised manuscript.

As you will see, the reviewers overall appreciate the findings and their interest to the field. However, they nonetheless raise a number of concerns, which must be resolved in the revised version of the manuscript. In particular, it will be important to clarify the issues raised regarding Figure 5(F). During cross-commenting the referees agreed that dCTP levels are lower than CTP levels (not "as low as" as noted in ref#1 comment 1), but nonetheless argued that it will be crucial to "explain normal mtDNA levels in spite of marked shortage of dCTP". In addition, regarding referee #3's major concern- we will not require xenograft or in vivo models to proceed with the manuscript. However, we strongly encourage you to consider experimentally addressing this point, in particular in light of the proposed potential as a therapeutic target. In our view this would strengthen the manuscript and expand the potential readership. Please carefully consider all referee comments and revise the manuscript and figures as needed, as well as providing a detailed response to each comment. Please also remember that the revised manuscript should fulfill all EMBO Journal formatting requirements when it is next submitted (please see below and: https://www.embopress.org/page/journal/14602075/authorguide#submissionofrevisions)

Please note that it is our policy to allow only a single round of major revision. Acceptance depends on a positive outcome of a second round of review and therefore on the completeness of your responses included in the next, final version of the manuscript. Thus it is important to clarify any questions and concerns as early as possible during the revision, and I invite you to contact me to discuss a revision plan as soon as possible to clarify any questions or potential uncertainties about the revision. In this regard, I would also like to let you know that I will be leaving EMBO Journal this February and I will hand over your manuscript to one of my current colleagues. This will not affect any decisions on your study. If we discuss a revision plan or any specific points beforehand, anything we agree upon will be noted and accessible to the new handling editor.

Thank you for the opportunity to consider your work for publication. Please contact me if you would like to discuss any points further or have any questions.

Kind regards,
Stefanie

Stefanie Boehm
Editor
The EMBO Journal

***IMPORTANT NOTE: we now perform an initial quality control of all revised manuscripts before re-review. Your manuscript will FAIL this control and the handling will be DELAYED if the following APPLIES:

1) A data availability section is missing. (The section must be named "Data Availability" and must have the required format, detailed below). Please provide either a referee token or public access to all data.
2) Your manuscript contains error bars based on n=2. Please use scatter blots showing the individual datapoints in these cases. The use of statistical tests needs to be justified.

When submitting your revised manuscript, please carefully review the instructions that follow below. Failure to include requested

items will delay the evaluation of your revision.***

-

When submitting your revised manuscript, please carefully review the instructions below and include the following items:

1) A .docx formatted version of the manuscript text (including legends for main figures, EV figures and tables). Please make sure that the changes are highlighted to be clearly visible.

2) Individual production quality figure files as .eps, .tif, .jpg (one file per figure). *Please remove figures from the main manuscript*

3) A .docx formatted letter INCLUDING the reviewers' reports and your detailed point-by-point response to their comments. As part of the EMBO Press transparent editorial process, the point-by-point response is part of the Review Process File (RPF), which will be published alongside your paper.

4) A complete author checklist, which you can download from our author guidelines (https://wol-prod-cdn.literatumonline.com/pb-assets/embo-site/Author%20Checklist%20-%20EMBO%20J-1561436015657.xlsx). Please insert information in the checklist that is also reflected in the manuscript. The completed author checklist will also be part of the RPF.

5) All corresponding authors are required to supply an ORCID ID for their name upon submission of a revised manuscript.

6) At EMBO Press we ask authors to provide source data for the main manuscript figures. Our source data coordinator will contact you to discuss which figure panels we would need source data for and will also provide you with helpful tips on how to upload and organize the files.

7) Before submitting your revision, primary datasets produced in this study need to be deposited in an appropriate public database. The required format for this section is:

[data type]: [name of the resource] [accession number/identifier/doi] ([URL or identifiers.org/DATABASE:ACCESSION])
*** Note - All links should resolve to a page where the data can be accessed. ***

Please also see https://www.embopress.org/page/journal/14602075/authorguide#datadeposition Please note that the Data Availability Section is restricted to new primary data that are part of this study.

8) When assembling figures, please refer to our figure preparation guideline in order to ensure proper formatting and readability in print as well as on screen:
https://bit.ly/EMBOPressFigurePreparationGuideline
See also guidelines for figure legends: https://www.embopress.org/page/journal/14602075/authorguide#figureformat

Please remember: Digital image enhancement is acceptable practice, as long as it accurately represents the original data and conforms to community standards. If a figure has been subjected to significant electronic manipulation, this must be noted in the figure legend or in the 'Materials and Methods' section. The editors reserve the right to request original versions of figures and the original images that were used to assemble the figure.

9) We replaced Supplementary Information with Expanded View (EV) Figures and Tables that are collapsible/expandable online (see examples in http://msb.embopress.org/content/11/6/812). A maximum of 5 EV Figures can be typeset. EV Figures should be cited as 'Figure EV1, Figure EV2" etc.. in the text and their respective legends should be included in the main text after the legends of regular figures.

- For the figures that you do NOT wish to display as Expanded View figures, they should be bundled together with their legends in a single PDF file called *Appendix*, which should start with a short Table of Content. Appendix figures should be referred to in the main text as: "Appendix Figure S1, Appendix Figure S2" etc. See detailed instructions regarding expanded view here: <
https://www.embopress.org/page/journal/14602075/authorguide#expandedview>.

- Additional Tables/Datasets should be labeled and referred to as Table EV1, Dataset EV1, etc. Legends have to be provided in a separate tab in case of .xls files. Alternatively, the legend can be supplied as a separate text file (README) and zipped together with the Table/Dataset file.
** We suggest uploading table S1, S2, S3 as datasets. **

10) Our journal encourages inclusion of *data citations in the reference list* to directly cite datasets that were re-used and obtained from public databases. Data citations in the article text are distinct from normal bibliographical citations and should directly link to the database records from which the data can be accessed. In the main text, data citations are formatted as follows: "Data ref: Smith et al, 2001" or "Data ref: NCBI Sequence Read Archive PRJNA342805, 2017". In the Reference list, data citations must be labeled with "[DATASET]". A data reference must provide the database name, accession number/identifiers and a resolvable link to the landing page from which the data can be accessed at the end of the reference. Further instructions are available at < https://www.embopress.org/page/journal/14602075/authorguide#referencesformat >.

Further information is available in our Guide For Authors:
https://www.embopress.org/page/journal/14602075/authorguide

****
In the interest of ensuring the conceptual advance provided by the work, we recommend submitting a revision within 3 months (1st May 2023). Please discuss the revision progress ahead of this time with the editor if you require more time to complete the revisions. Use the link below to submit your revision:

Link Not Available
****

Referee #1:

The study uncovers an important, unanticipated, function of a nucleotide salvage pathway enzyme in mitochondria, NME6. First the authors show that NME6 becomes essential for mtDNA maintenance when solute carriers for pyrimidines are deleted in line with other salvage pathway enzymes. More interestingly and unexpectedly the NME6 KO impairs OXPHOS, mitochondrial transcription and cell growth in conditions where the solute carriers are present and there is no substantial decrease in mtDNA copy number. The adverse effects of the KO are largely corrected by transgenic expressed NME6, but the main strength of the paper is that in Figure 5 the authors show that pyrimidines are low in the NME6 deficient cells/mitochondria and that C or U supplementation is sufficient to restore cytochrome b transcript levels and respiration. To my knowledge this is the second mitochondrial gene defect that can be rescued by ribonucleotide supplementation (after a study of YME1L by the same authors), and the first where it is shown to increase transcription (based on 1 mitochondrial mRNA).

There are some puzzling results that merit more discussion and ideally some further analysis.

• The dCTP level is as low as the CTP level in mitochondria (Figure 5F). How do the authors explain the normal mtDNA level in the face of this marked shortage of an essential DNA precursor? The model in 5I needs to reflect the fact that one of the dNTPs is lacking in the absence of NME6.
• Do U and C/dC supplementation rescue cell growth?
• Why, when UTP is no lower than GTP (5F), does uridine supplementation work as well as cytidine (Figure 5G)?
• Does C and/or U supplementation restore dCTP levels in mitochondria?
• More transcripts than CYTB should be analyzed (Figure 5G) when the restoration of transcription by nucleoside supplementation is one of the most important claims.

On page 20 assign numeric values to the "dramatic depletion of CTP and dCTP and a mild reduction of UTP".

The information about TFAM on page 7 adds little of value and distracts from the main subject of the manuscript.

The article has not been written to the usual standard of Dr Langer's group. The following points are not exhaustive.

The authors make heavy weather of the lead up to the straightforward idea of checking mitochondrial transcripts and translation products in the context of OXPHOS deficiency, something that is standard procedure. Therefore, be much more direct; e.g. state that the possibility that NME6 might affect OXPHOS via transcription or translation were supported by Gene coessentiality network analysis (and move the FIREWORKS data to Supplemental data).

The Discussion is weak. Much of it repeats results or meanders into areas that are not central to the main findings.

In the Discussion the authors state: "Our results thus reveal two independent roles of NME6 for the maintenance and expression

of the mitochondrial genome." This is essentially reiterating the results and I would suggest it misses the bigger point that the results speak to the overlaps between deoxynucleotide/nucleotide homeostasis. For this reviewer, the study highlights the need to consider both RNA and DNA synthesis when investigating nucleotide metabolising enzymes and mitochondrial dysfunction - do the authors agree this is one of the main conclusions?

The part beginning: "While the specific enzymatic activity of NME6 remains to be dissected..." is verbose. Consider going directly to the point that NME6 is redundant in HEPG2 cells implying "alternative pathways can support the maintenance of purine [sic] rNTPs in the absence of NME6". Higher cytosolic NTP synthesis is a possibility.

"Alternatively, the loss of mtDNA may result from severe transcription deficiency in mitochondria lacking pyrimidine transporters and NME6 since mtDNA replication requires the synthesis of RNA primers by POLRMT." Delete this part, which is weak. The authors show that several kilobases of the polycistronic transcript are synthesized without problems, therefore, without data there is no reason to suppose that shorter primers will be affected.

It is not worth speculating why NME6 is not a MDS gene, it might prove to be in the future, and the authors note that it has not been determined whether mtDNA depletion occurs in quiescent tissues in the absence of NME6.

"Our CRISPR-SpCas9 screen revealed that NME6 is also required for the maintenance of mtDNA in cells lacking the mitochondrial pyrimidine transporters, SLC25A33 and SLC25A36. It is likely that mtDNA replication depends on dNTP production by NME6 when mitochondrial pyrimidine import is blocked." This is results and the remainder of the long paragraph about NME4 and inflammation is speculation more suitable to a review article.

In summary, a more succinct discussion focusing on the clear implications of the current study and the principal questions arising would serve readers much better.

The abstract ends with: "Our work proposes NME6 and mitochondrial nucleotide metabolism to be untapped therapeutic targets in diseases associated with aberrant mitochondrial gene expression including cancer and autoimmune disorders." As there are no experiments in models of cancer or autoimmune disorders this statement belongs in the Discussion if at all and the authors need to find a conclusion for the Abstract that stems directly from the study.

In the introduction delete 'pre-existing'; replace 'sweeping generalisation' and delete 'poorly characterised'.

In the introduction it takes most of 2 pages to arrive at the subject of the main finding of the article: "but we know very little regarding the regulation of rNTP supply for mitochondrial transcription". Next instead of being told what little is know, we are treated to a brief description of POLRMT and the paragraph ends with the information that upregulation of POLRMT and TEFM have been reported in several cancers and that mitochondrial transcription is an exciting potential therapeutic target in cancer. In the field of cancer innumerable factors have been reported to be up or down regulated and to be exciting potential therapeutic cancer targets on that basis. Thus, the authors need to explain why it is an exceptional prospect or remove these remarks.

From the end of the introduction: "Strikingly, NME6 is required for constitutive mtRNA synthesis and OXPHOS function in [some] proliferating cells [but not others]."

Consider rewording: incompletion of polycistronic transcripts.

Referee #2:

In this manuscript, Grotehans et al. investigate the role of mitochondrial nucleoside diphosphate kinase, NME6, in the maintenance of mitochondrial NTP pools necessary for the transcription of mitochondrial genes. The authors convincingly show that inactivation of NME6 leads to the depletion of mitochondrial transcripts, which can be suppressed upon supplementation with NTPs, or all four canonical ribonucleosides plus thymidine or pyrimidine ribonucleosides alone. This is a solid study expanding our understanding of mitochondrial nucleotide metabolism with the data largely supporting the conclusions. I have mostly minor comments concerning certain wordings or interpretations.

In general, words (deoxy)nucleoside and (deoxy)nucleotide (deoxynucleoside with one or more phosphate groups) are often used in place of each other, as detailed below.

Page 2, sentence "Perturbation of NME6 leads to the depletion of mitochondrial transcripts, destabilisation of the electron

transport chain and impaired oxidative phosphorylation; deficiencies which are suppressed upon supplementation with pyrimidine ribonucleotides". Technically speaking, the authors never tested if pyrimidine ribonucleotides could suppress these deficiencies. The experiments were done either with all four ribonucleotides or with pyrimidine ribonucleoSides, so "supplementation with pyrimidine ribonucleotides" should be changed to "supplementation with pyrimidine ribonucleosides".

Page 4, sentence "Enhanced uptake of mitochondrial pyrimidines can cause nuclear genomic instability due to a depletion of cytosolic dNTPs, Ref 14". Please note that this finding (depletion of cytosolic dNTPs due to an enhanced uptake of mitochondrial pyrimidines) has been disputed by PMID: 32778836, which must be mentioned.

Page 7, sentence "Two outliers corresponded to sgRNA targeting the mitochondrial nucleoside diphosphate kinases (NDPK), NME4 and NME6, which suggested that their loss renders cells unable to maintain mtDNA levels when pyrimidine import is blocked (Fig. 1E)" is confusing. What will NME4 and NME6 phosphorylate in mitochondria if no pyrimidines are imported? Perhaps the authors mean "pyrimidine nucleotide import is blocked"? Note that pyrimidines can mean nucleobases, nucleosides or nucleotides.

Page 8, sentence "Collectively, these data demonstrate that NME6 maintains the mitochondrial genome upon reduced pyrimidine nucleotide supply from the cytosol and indicate that NME6 catalyses the final enzymatic step in the salvage of nucleotides within mitochondria (Fig 1A)" is unclear. What brings the nucleotides for salvage into the mitochondria, if pyrimidine nucleotide supply from the cytosol is reduced? Or do the authors mean "the final enzymatic step in the salvage of nucleosides within mitochondria"?

Page 17, the authors state "Heavy-strand RNA transcripts up to ATP8 were barely affected and the levels of the promoter proximal 12S and 16S rRNA were higher in NME6-depleted cells (Fig. 4E)". However, there is first a drop in the level of ND2 transcript, followed by an increase in the CO1 transcript. Is there an explanation to this variation?

Page 20, sentence "We therefore treated cells with a mix of precursor nucleosides that included the four ribonucleosides cytidine, uridine, guanosine and adenosine and the deoxyribonucleoside thymidine". What was the logic behind inclusion of thymidine (especially since dTTP was not included in the NTP treatment)? Note that thymidine can interfere with DNA replication as used in the double thymidine block.

Page 20, sentence "Mitochondria lacking NME6 had a dramatic depletion of CTP and dCTP...". A dramatic reduction in dCTP levels should lead to a strong depletion of mtDNA. However, no such depletion was found (e.g., page 8, sentence "Conversely, while NME6 depletion alone did not reduce mtDNA levels ... (Fig. 1G) or page 11, sentence "The increased dependency on NME6 in galactose or HPLM medium was independent of mtDNA levels, which remained normal in cells lacking NME6 regardless of the growth medium (Fig. 2E, F) "). How can this discrepancy be explained?

Page 24, sentence "We demonstrate an unexpected regulatory role of the mitochondrial nucleotide diphosphate kinase NME6 for mitochondrial transcription". In what way is NME6's role regulatory? To this reviewer the role of NME6 appears to be primarily metabolic. Also, its role for for mitochondrial transcription is quite expected, as NME6's function is to make the final step in the synthesis of nucleoside triphosphates, the building blocks of RNA. Note that the word "nucleotide" should be changed to "nucleoside" in the cited sentence.

Page 24, sentence "Moreover, NME6 is required for the maintenance of mtDNA and provides dNTPs for mtDNA replication when pyrimidine import from the cytosol is limited". Technically, it has not been shown that NME6 provides (all four) dNTPs when pyrimidine nucleotide import is limited, as the dNTP levels have not been measured in the triple knockout cells. The conclusion is inferred from the results in the sentence on page 8 "Conversely, while NME6 depletion alone did not reduce mtDNA levels, the combined knockout of NME6, SLC25A33 and SLC25A36 caused a dramatic loss of mtDNA to 15-25 % of WT levels (Fig. 1G)" and thus should be rephrased. Also, "pyrimidine import" in the sentence on page 24 should be changed to "pyrimidine nucleotide import". In general, there are about 8 instances in the manuscript where the authors write "pyrimidine import" but presumably mean "pyrimidine nucleotide import" as pyrimidine - as mentioned above - can mean the nucleobase, the nucleoside or the nucleotide. This should be corrected.

Page 24, sentence "The majority of mitochondrial transcripts are depleted in cells lacking NME6, apart from the rRNAs and the four mRNAs most proximal to the HSP (ND1, ND2, CO1 and CO2)." As shown in Fig. 4E and mentioned in one of the previous comments, ND2 is also depleted. This should be commented on.

Page 24, sentence "The restoration of mitochondrial transcript levels in NME6 depleted cells by treatment with exogenous nucleosides or ribonucleotides is strong evidence that NME6 is a primary supplier of rNTPs for mitochondrial transcription". It would be more accurate to write "exogenous ribonucleosides or ribonucleotides".

Page 28, sentence "It remains to be seen whether NME6 is essential for mtDNA synthesis in quiescent tissues that suppress de novo synthesis of nucleotides and depend on mitochondrial nucleotide salvage for the provision of dNTPs for mtDNA synthesis" - it would be more accurate to write "It remains to be seen whether NME6 is essential for mtDNA synthesis in quiescent tissues that suppress de novo synthesis of DEOXYRIBOnucleotides and depend on mitochondrial NUCLEOSIDE salvage for the

provision of dNTPs for mtDNA synthesis".

Referee #3:

Evaluation summary

In their manuscript titled "Ribonucleotide synthesis by NME6 fuels mitochondrial gene expression", Grotehans et al discovered through a targeted CRISPR-SpCas9 screen on SLC25A33/SLC25A36 KO HeLa cells that mitochondrial nucleoside diphosphate kinase NME6 is required for the maintenance of mtDNA when mitochondrial pyrimidine import is blocked. They additionally show that NME6 supplies rNTPs for mitochondrial transcription and is required for respiration and the maintenance of OXPHOS subunits in proliferating cells in vitro. These impairments can be rescued bey supplementing the NME6 KO HeLa cells with pyrimidine ribonucleotides. The paper is well-written, and the findings are very convincing. The authors are to be congratulated on their elegant work. Their findings are also potential importance given NME6 may serve a target to reduce OXPHOS function in rapidly dividing cancer cells, as the authors suggest. This last assertion remains very speculative, however. The paper would be strengthened if they could demonstrate more directly the physiologic relevance of their findings.

Major concerns

1. The experiments done in this paper are purely in vitro and so the authors assertion that NME6 is a potential therapeutic target for cancer is very speculative. Xenograft or other in vivo experiments may help show therapeutic relevance more directly. At a minimum it is important to assess whether NME6 KO or knockdown causes a more severe OXPHOS defect in rapidly dividing cells compared to slowly dividing or post-mitotic cells?

Minor concerns

1. In Figure 3B, can depletion of the 45 OXPHOS subunits be explained by loss of the the mtDNA. For instance, are they the same subunits that are observed to be destabilized in Rh0 cells (e.g., following annotation using the dataset from PMID: 34542926)?
2. Although not strictly necessary in my view for the revision, it would be interesting to know whether the stress response highlighted in Figure S3 A and B is DELE1 dependent.

Additional non-essential suggestions

1. Figure 5 A and 5C. It is not clear what "+" and "-" mean in the graphs. I recommend labeling with "Nucs" like you did in Figure 5D.
2. Although not strictly necessary in my view for the revision, it would be interesting to know whether the stress response highlighted in Figure S3 A and B is DELE1 dependent.
3. A Related to the observation that NME6 associates with RCC1L and leads to a reduction in RNA granules. Does NME6 itself show a punctate pattern in the mitochondrial matrix and if so does it co-localize with mitochondrial RNA granules? Does the phosphorylation deficient mutant have the same localization pattern? This experiment is also not strictly necessary for the revision in my view but would enhance the manuscript.
4. Related to the observation that NME6 associates with RCC1L and leads to a reduction in RNA granules. Does NME6 itself show a punctate pattern in the mitochondrial matrix and if so does it co-localize with mitochondrial RNA granules? Does the phosphorylation deficient mutant have the same localization pattern? This experiment is also not strictly necessary for the revision in my view but would enhance the manuscript.

Referee #1:

The study uncovers an important, unanticipated, function of a nucleotide salvage pathway enzyme in mitochondria, NME6. First the authors show that NME6 becomes essential for mtDNA maintenance when solute carriers for pyrimidines are deleted in line with other salvage pathway enzymes. More interestingly and unexpectedly the NME6 KO impairs OXPHOS, mitochondrial transcription and cell growth in conditions where the solute carriers are present and there is no substantial decrease in mtDNA copy number. The adverse effects of the KO are largely corrected by transgenic expressed NME6, but the main strength of the paper is that in Figure 5 the authors show that pyrimidines are low in the NME6 deficient cells/mitochondria and that C or U supplementation is sufficient to restore cytochrome b transcript levels and respiration. To my knowledge this is the second mitochondrial gene defect that can be rescued by ribonucleotide supplementation (after a study of YME1L by the same authors), and the first where it is shown to increase transcription (based on 1 mitochondrial mRNA).

We thank the reviewer for their positive evaluation of our study. We have performed new experiments and edited the text to address their comments.

There are some puzzling results that merit more discussion and ideally some further analysis.

• The dCTP level is as low as the CTP level in mitochondria (Figure 5F). How do the authors explain the normal mtDNA level in the face of this marked shortage of an essential DNA precursor?

We agree that this result is intriguing and therefore now discuss this observation in our manuscript (p. 17/18): "*We speculate that proliferating cells lacking NME6 are still able to maintain sufficient dCTP supply for mtDNA replication via the import of cytosolic dCTP.*". We wish to point out that we do not know the nucleotide specificity of NME6 nor the molar concentrations of the mitochondrial nucleotides and we therefore cannot compare the amount of dCTP vs CTP in our analysis. Nucleotide pool sizes differ between nucleotide species and likely vary according to cell type and metabolic status.

The model in 5I needs to reflect the fact that one of the dNTPs is lacking in the absence of NME6.

We have revised the schematic and highlight now the central role of NME6 for mitochondrial transcription and the accumulation of OXPHOS complexes (new Fig. 6).

• Do U and C/dC supplementation rescue cell growth?

To address this question, we performed growth assays in WT and *NME6* knockout cells supplemented with each nucleoside species. We found that U and C, but not dC, partially improved cell growth (new Fig EV5F), in accordance with the rescue of mitochondrial transcripts and OXPHOS in these cells. We comment on the partial restoration of growth in the text (p. 15): "*We quantified mitochondrial nucleotides in cells supplemented with cytidine or uridine by LC-MS and confirmed that both treatments boosted mitochondrial CTP and dCTP levels in NME6 knockout cells (Fig EV5E). Neither nucleoside could fully restore mitochondrial CTP/dCTP in NME6 knockout cells however, which may explain the partial improvement in cell growth upon treatment with cytidine or uridine (Fig EV5F).*"

• Why, when UTP is no lower than GTP (5F), does uridine supplementation work as well as cytidine (Figure 5G)?

We agree that NME6 does not significantly influence mitochondrial UTP and GTP levels (Fig 5F) and now describe our metabolomics data more accurately in the text (p. 14/15). As mentioned above, we have performed new LC/MS analysis of nucleotides in whole cell and mitochondrial fractions from cells supplemented with the pyrimidine nucleosides (new Fig EV5E). Uridine or cytidine supplementation enhance mitochondrial CTP levels in NME6 knockout cells to a similar extent (new Fig EV5E), which explains why

uridine or cytidine can rescue mitochondrial transcripts and OXPHOS. We further write on p.15 that *"Uridine treatment can increase both cellular UTP and CTP levels (Pooler et al, 2005), since UTP is readily converted to CTP by the cytosolic enzyme CTP synthetase (CTPS)."*

• Does C and/or U supplementation restore dCTP levels in mitochondria?

Our new data show that cytidine or uridine supplementation can enhance mitochondrial dCTP levels in NME6 knockout cells (new Fig 5H). The effect of cytidine or uridine supplementation on all detected nucleotide species are also presented in new Appendix Fig. S1.

• More transcripts than CYTB should be analyzed (Figure 5G) when the restoration of transcription by nucleoside supplementation is one of the most important claims.

We agree and therefore include the analysis of ATP8, ATP6, COX3, ND3, ND4 and ND6 transcript levels (new Fig EV5E). All transcripts behave similarly to CYTB and are restored in NME6 knockout cells upon cytidine or uridine supplementation.

On page 20 assign numeric values to the "dramatic depletion of CTP and dCTP and a mild reduction of UTP".
We have included percentage reductions for CTP and dCTP in the text on p. 14. As mentioned above, we now state that there is little change in UTP (like GTP), which more accurately reflects the data.

The information about TFAM on page 7 adds little of value and distracts from the main subject of the manuscript.
We agree that some details of TFAM were superfluous and have trimmed this part (p. 7). We still describe its performance within the sgRNA library because it helps the reader understand our rationale for using the area of mtDNA puncta as an alternative readout in our screen.

The article has not been written to the usual standard of Dr Langer's group. The following points are not exhaustive.

The authors make heavy weather of the lead up to the straightforward idea of checking mitochondrial transcripts and translation products in the context of OXPHOS deficiency, something that is standard procedure. Therefore, be much more direct; e.g. state that the possibility that NME6 might affect OXPHOS via transcription or translation were supported by Gene coessentiality network analysis (and move the FIREWORKS data to Supplemental data).

We have carefully revised the manuscript text and reorganized Fig 4. Now, we demonstrate first the reduced synthesis of mtDNA-encoded subunits in NME6 knockout cells (Fig 4A,B), which allows us to directly and concisely question the role of transcription and translation in the text (p. 12). We now show the FIREWORKS data, which highlight the unique role of NME6 within the NME family for mitochondrial gene expression, to Fig EV4A.

The Discussion is weak. Much of it repeats results or meanders into areas that are not central to the main findings.
We thank the reviewer for their constructive criticism. We have carefully rewritten the entire discussion and believe that the content and readability are greatly improved. We detail some of the most significant changes below.

In the Discussion the authors state: "Our results thus reveal two independent roles of NME6 for the

maintenance and expression of the mitochondrial genome." This is essentially reiterating the results and I would suggest it misses the bigger point that the results speak to the overlaps between deoxynucleotide/nucleotide homeostasis. For this reviewer, the study highlights the need to consider both RNA and DNA synthesis when investigating nucleotide metabolising enzymes and mitochondrial dysfunction - do the authors agree this is one of the main conclusions?

We agree with the reviewer that this is one of the main conclusions from our work and have highlighted this in the first and final paragraphs of our discussion. We have removed repetitive “results-like” bodies of text throughout the discussion.

The part beginning: "While the specific enzymatic activity of NME6 remains to be dissected..." is verbose. Consider going directly to the point that NME6 is redundant in HEPG2 cells implying "alternative pathways can support the maintenance of purine [sic] rNTPs in the absence of NME6". Higher cytosolic NTP synthesis is a possibility.

We have removed this part and minimised speculation on particular differences between cell types (p. 16).

"Alternatively, the loss of mtDNA may result from severe transcription deficiency in mitochondria lacking pyrimidine transporters and NME6 since mtDNA replication requires the synthesis of RNA primers by POLRMT." Delete this part, which is weak. The authors show that several kilobases of the polycistronic transcript are synthesized without problems, therefore, without data there is no reason to suppose that shorter primers will be affected.

We have removed this part.

It is not worth speculating why NME6 is not a MDS gene, it might prove to be in the future, and the authors note that it has not been determined whether mtDNA depletion occurs in quiescent tissues in the absence of NME6.

We have removed this part.

"Our CRISPR-SpCas9 screen revealed that NME6 is also required for the maintenance of mtDNA in cells lacking the mitochondrial pyrimidine transporters, SLC25A33 and SLC25A36. It is likely that mtDNA replication depends on dNTP production by NME6 when mitochondrial pyrimidine import is blocked." This is results and the remainder of the long paragraph about NME4 and inflammation is speculation more suitable to a review article.

We have deleted the sentence and removed distracting discussion of NME4. We believe that a possible link between NME6 and mitochondrial-related inflammatory pathways is an exciting avenue to explore in the future and briefly discuss this on p. 18.

In summary, a more succinct discussion focusing on the clear implications of the current study and the principal questions arising would serve readers much better.

The abstract ends with: "Our work proposes NME6 and mitochondrial nucleotide metabolism to be untapped therapeutic targets in diseases associated with aberrant mitochondrial gene expression including cancer and autoimmune disorders." As there are no experiments in models of cancer or autoimmune disorders this

statement belongs in the Discussion if at all and the authors need to find a conclusion for the Abstract that stems directly from the study.

We agree and have deleted this sentence.

In the introduction delete 'pre-existing'; replace 'sweeping generalisation' and delete 'poorly characterised'.

We have deleted these phrases.

In the introduction it takes most of 2 pages to arrive at the subject of the main finding of the article: "but we know very little regarding the regulation of rNTP supply for mitochondrial transcription". Next instead of being told what little is know, we are treated to a brief description of POLRMT and the paragraph ends with the information that upregulation of POLRMT and TEFM have been reported in several cancers and that mitochondrial transcription is an exciting potential therapeutic target in cancer. In the field of cancer innumerable factors have been reported to be up or down regulated and to be exciting potential therapeutic cancer targets on that basis. Thus, the authors need to explain why it is an exceptional prospect or remove these remarks.

We have carefully edited the introduction to be more direct and succinct. We have removed unnecessary detail on POLRMT and TEFM.

From the end of the introduction: "Strikingly, NME6 is required for constitutive mtRNA synthesis and OXPHOS function in [some] proliferating cells [but not others]."

We have deleted this sentence.

Consider rewording: incompletion of polycistronic transcripts.

We have deleted this phrase.

Referee #2:

In this manuscript, Grotehans et al. investigate the role of mitochondrial nucleoside diphosphate kinase, NME6, in the maintenance of mitochondrial NTP pools necessary for the transcription of mitochondrial genes. The authors convincingly show that inactivation of NME6 leads to the depletion of mitochondrial transcripts, which can be suppressed upon supplementation with NTPs, or all four canonical ribonucleosides plus thymidine or pyrimidine ribonucleosides alone. This is a solid study expanding our understanding of mitochondrial nucleotide metabolism with the data largely supporting the conclusions. I have mostly minor comments concerning certain wordings or interpretations.

We thank the reviewer for their positive evaluation of our study. We have carefully edited the text and provide new data to address their concerns.

In general, words (deoxy)nucleoside and (deoxy)nucleotide (deoxynucleoside with one or more phosphate groups) are often used in place of each other, as detailed below.

Page 2, sentence "Perturbation of NME6 leads to the depletion of mitochondrial transcripts, destabilisation of the electron transport chain and impaired oxidative phosphorylation; deficiencies which are suppressed upon supplementation with pyrimidine ribonucleotides". Technically speaking, the authors never tested if

pyrimidine ribonucleotides could suppress these deficiencies. The experiments were done either with all four ribonucleotides or with pyrimidine ribonucleoSides, so "supplementation with pyrimidine ribonucleotides" should be changed to "supplementation with pyrimidine ribonucleosides".

We appreciate these being pointed out and have improved our accuracy in the use of nucleoside/nucleotide nomenclature.

Page 4, sentence "Enhanced uptake of mitochondrial pyrimidines can cause nuclear genomic instability due to a depletion of cytosolic dNTPs, Ref 14". Please note that this finding (depletion of cytosolic dNTPs due to an enhanced uptake of mitochondrial pyrimidines) has been disputed by PMID: 32778836, which must be mentioned.

We now refer to the study by Sharma et al (PMID: 32778836) on p.4: "*Expression of proofreading-deficient mtDNA polymerase gamma enhances the uptake of mitochondrial dNTPs, which results in the depletion of cytosolic dNTPs and nuclear genomic instability (Hamalainen et al, 2019). This was observed in mouse stem cells but not in whole mouse embryos, pointing to cell type specific regulation of mitochondrial dNTP levels (Sharma et al, 2020).*"

Page 7, sentence "Two outliers corresponded to sgRNA targeting the mitochondrial nucleoside diphosphate kinases (NDPK), NME4 and NME6, which suggested that their loss renders cells unable to maintain mtDNA levels when pyrimidine import is blocked (Fig. 1E)" is confusing. What will NME4 and NME6 phosphorylate in mitochondria if no pyrimidines are imported? Perhaps the authors mean "pyrimidine nucleotide import is blocked"? Note that pyrimidines can mean nucleobases, nucleosides or nucleotides.

We indeed meant "when pyrimidine nucleotide import is blocked" and have changed this sentence accordingly. We have also changed the title of this section to ***The maintenance of mtDNA depends on NME6 when pyrimidine <u>nucleotide</u> import is blocked.***

Page 8, sentence "Collectively, these data demonstrate that NME6 maintains the mitochondrial genome upon reduced pyrimidine nucleotide supply from the cytosol and indicate that NME6 catalyses the final enzymatic step in the salvage of nucleotides within mitochondria (Fig 1A)" is unclear. What brings the nucleotides for salvage into the mitochondria, if pyrimidine nucleotide supply from the cytosol is reduced? Or do the authors mean "the final enzymatic step in the salvage of nucleosides within mitochondria"?

We have edited the sentence on p. 7: *"Collectively, these data demonstrate that NME6 maintains the mitochondrial genome, if the supply of pyrimidine nucleotides from the cytosol is limited, and indicate that NME6 generates dNTPs within mitochondria (Fig 1A)."*

Page 17, the authors state "Heavy-strand RNA transcripts up to ATP8 were barely affected and the levels of the promoter proximal 12S and 16S rRNA were higher in NME6-depleted cells (Fig. 4E)". However, there is first a drop in the level of ND2 transcript, followed by an increase in the CO1 transcript. Is there an explanation to this variation?

This observation led us to analyse mitochondrial transcripts in HLE cells for comparison (new Fig EV4C). The effect of NME6 loss on mitochondrial transcript levels in HLE show a similar trend to HeLa, which we note on p.13: "*Mitochondrial transcripts were also depleted in NME6 knockout HLE cells and the fold change reduction of heavy-strand transcripts was greatest for those furthest from the promoter (Fig EV4C).*" However, CO1 is reduced in NME6 knockout HLE, which, as the reviewer noticed, is not the case in HeLa. There may be a number of explanations for variations between individual transcript steady-state levels upon NME6 depletion. We therefore decided to focus on the general trend that we observed in both cell types and edited the text accordingly on p. 13: "*We measured the levels of mitochondrial messenger RNA (mRNA) and*

ribsosomal RNA (rRNA) by qPCR and observed a striking pattern of mitochondrial mRNA depletion in cells lacking NME6 that correlated with the distance from the heavy strand promoter (Fig 4E, F). Heavy-strand mRNAs from ATP8 onwards, as well as ND6 on the light-strand, were significantly lower in the absence of NME6 compared to WT and NME6-MycFlag complemented cells (Fig 4F)."

Page 20, sentence "We therefore treated cells with a mix of precursor nucleosides that included the four ribonucleosides cytidine, uridine, guanosine and adenosine and the deoxyribonucleoside thymidine". What was the logic behind inclusion of thymidine (especially since dTTP was not included in the NTP treatment)? Note that thymidine can interfere with DNA replication as used in the double thymidine block.

A pre-mixed solution of nucleosides was used for these experiments and included thymidine (EmbryoMax 100 x Nucleosides, Millipore, ES008D). We therefore also tested the impact of individual ribonucleosides on the NME6-dependent phenotypes.

Page 20, sentence "Mitochondria lacking NME6 had a dramatic depletion of CTP and dCTP...". A dramatic reduction in dCTP levels should lead to a strong depletion of mtDNA. However, no such depletion was found (e.g., page 8, sentence "Conversely, while NME6 depletion alone did not reduce mtDNA levels ... (Fig. 1G) or page 11, sentence "The increased dependency on NME6 in galactose or HPLM medium was independent of mtDNA levels, which remained normal in cells lacking NME6 regardless of the growth medium (Fig. 2E, F) "). How can this discrepancy be explained?

We agree that this result is intriguing and therefore now discuss this observation in our manuscript (p. 17/18): *"We speculate that proliferating cells lacking NME6 are still able to maintain sufficient dCTP supply for mtDNA replication via the import of cytosolic dCTP. NME6 nevertheless becomes essential for the maintenance of mtDNA in cells lacking the mitochondrial pyrimidine transporters, SLC25A33 and SLC25A36."*. We wish to point out that we do not know the molar concentrations of the mitochondrial nucleotides and we therefore cannot determine on the size of the dCTP pool within mitochondria. Nucleotide pool sizes differ between nucleotide species and likely vary according to cell type and metabolic status.

Page 24, sentence "We demonstrate an unexpected regulatory role of the mitochondrial nucleotide diphosphate kinase NME6 for mitochondrial transcription". In what way is NME6's role regulatory? To this reviewer the role of NME6 appears to be primarily metabolic. Also, its role for for mitochondrial transcription is quite expected, as NME6's function is to make the final step in the synthesis of nucleoside triphosphates, the building blocks of RNA. Note that the word "nucleotide" should be changed to "nucleoside" in the cited sentence.

We agree that a regulatory role of NME6 remains speculative and have deleted this sentence.

Page 24, sentence "Moreover, NME6 is required for the maintenance of mtDNA and provides dNTPs for mtDNA replication when pyrimidine import from the cytosol is limited". Technically, it has not been shown that NME6 provides (all four) dNTPs when pyrimidine nucleotide import is limited, as the dNTP levels have not been measured in the triple knockout cells. The conclusion is inferred from the results in the sentence on page 8 "Conversely, while NME6 depletion alone did not reduce mtDNA levels, the combined knockout of NME6, SLC25A33 and SLC25A36 caused a dramatic loss of mtDNA to 15-25 % of WT levels (Fig. 1G)" and thus should be rephrased.

We now state more accurately on p.16 that NME6 *"is required for the maintenance of mtDNA when pyrimidine dNTP import from the cytosol is limited."*. We also discuss our data more accurately by focussing on the relevance of dCTP supply by NME6 knockout cells rather than all dNTPs.

Also, "pyrimidine import" in the sentence on page 24 should be changed to "pyrimidine nucleotide import". In general, there are about 8 instances in the manuscript where the authors write "pyrimidine import" but

presumably mean "pyrimidine nucleotide import" as pyrimidine - as mentioned above - can mean the nucleobase, the nucleoside or the nucleotide. This should be corrected.

We have corrected these instances.

Page 24, sentence "The majority of mitochondrial transcripts are depleted in cells lacking NME6, apart from the rRNAs and the four mRNAs most proximal to the HSP (ND1, ND2, CO1 and CO2)." As shown in Fig. 4E and mentioned in one of the previous comments, ND2 is also depleted. This should be commented on.

We have deleted this sentence from the discussion and please see response to comment above.

Page 24, sentence "The restoration of mitochondrial transcript levels in NME6 depleted cells by treatment with exogenous nucleosides or ribonucleotides is strong evidence that NME6 is a primary supplier of rNTPs for mitochondrial transcription". It would be more accurate to write "exogenous ribonucleosides or ribonucleotides".

We have deleted this sentence

Page 28, sentence "It remains to be seen whether NME6 is essential for mtDNA synthesis in quiescent tissues that suppress de novo synthesis of nucleotides and depend on mitochondrial nucleotide salvage for the provision of dNTPs for mtDNA synthesis" - it would be more accurate to write "It remains to be seen whether NME6 is essential for mtDNA synthesis in quiescent tissues that suppress de novo synthesis of DEOXYRIBOnucleotides and depend on mitochondrial NUCLEOSIDE salvage for the provision of dNTPs for mtDNA synthesis".

We agree and have edited this sentence accordingly on p. 17. "*It remains to be seen whether NME6 is required for mtDNA synthesis in quiescent tissues that suppress de novo synthesis of deoxyribonucleotides and depend on mitochondrial nucleoside salvage for the provision of dNTPs for mtDNA synthesis (Ferraro et al., 2005; Mathews & Song, 2007; Pontarin et al, 2007).*"

Referee #3:

Evaluation summary
In their manuscript titled "Ribonucleotide synthesis by NME6 fuels mitochondrial gene expression", Grotehans et al discovered through a targeted CRISPR-SpCas9 screen on SLC25A33/SLC25A36 KO HeLa cells that mitochondrial nucleoside diphosphate kinase NME6 is required for the maintenance of mtDNA when mitochondrial pyrimidine import is blocked. They additionally show that NME6 supplies rNTPs for mitochondrial transcription and is required for respiration and the maintenance of OXPHOS subunits in proliferating cells in vitro. These impairments can be rescued bey supplementing the NME6 KO HeLa cells with pyrimidine ribonucleotides. The paper is well-written, and the findings are very convincing. The authors are to be congratulated on their elegant work. Their findings are also potential importance given NME6 may serve a target to reduce OXPHOS function in rapidly dividing cancer cells, as the authors suggest. This last assertion remains very speculative, however. The paper would be strengthened if they could demonstrate more directly the physiologic relevance of their findings.

We thank the reviewer for their positive evaluation of our study. We have performed new experiments and edited the text to address their comments.

Major concerns

1. The experiments done in this paper are purely in vitro and so the authors assertion that NME6 is a potential therapeutic target for cancer is very speculative. Xenograft or other in vivo experiments may help show therapeutic relevance more directly. At a minimum it is important to assess whether NME6 KO or knockdown causes a more severe OXPHOS defect in rapidly dividing cells compared to slowly dividing or post-mitotic cells?

In our original submission, we speculated that NME6 could be a potential therapeutic target for cancer based on in vitro assays in liver cancer cell lines and the positive correlation between NME6 expression and poor prognosis for liver cancer patients. Small molecule inhibition of mitochondrial transcription was recently shown to inhibit xenograft tumour growth in mice, with minimal toxicity to normal tissues (Bonekamp et al., 2020; PMID: 33328633). These data led us to speculate that proliferating cells may also be particularly susceptible to the inhibition of NME6. We compared the effect of NME6 knockout on mitochondrial transcripts in proliferating versus growth arrested HeLa cells (obtained by serum starvation). As shown in reviewer Fig 1, cell growth was effectively blocked after 24 and 48 h serum starvation. Serum starved WT reduced mitochondrial transcript levels compared to proliferating cells, whereas transcripts remained low in NME6 knockout cells. Mitochondrial DNA levels were reduced in all cell lines upon serum starvation. These experiments do not indicate to us that the loss of NME6 predominantly affects proliferating cells. We also initiated xenograft experiments using NME6 knockout HLE cells, which however remained inconclusive within the limited time of the revision.

We therefore agree with this reviewer that it remains too speculative at this stage to suggest any therapeutic relevance for targeting NME6 in cancer. Instead, we focus on the conceptual advance that our study provides by describing the contribution of ribonucleotide metabolism to mitochondrial gene expression and use the final paragraph of the discussion to suggest exciting avenues for future work, including NME6's role in cancer and innate immunity. These future studies will undoubtedly require more elegant in vivo models that could

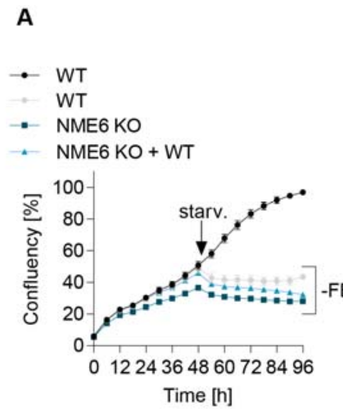

Reviewer Figure 1
A Analysis of cell growth arrest upon serum starvation (starv.) in the indicated HeLa cells. **B** Mitochondrial transcript levels in WT, NME6 KO and NME6 KO + WT HeLa cells measured by qPCR.

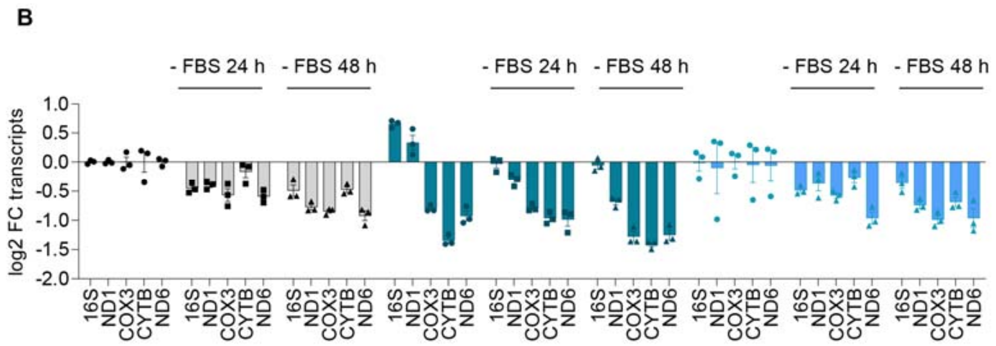

not be generated within the time frame of this revision.

Minor concerns

1. In Figure 3B, can depletion of the 45 OXPHOS subunits be explained by loss of the the mtDNA. For instance, are they the same subunits that are observed to be destabilized in Rh0 cells (e.g., following annotation using the dataset from PMID: 34542926)?

The comprehensive proteomic analysis of p0/Rho0 cells by Guerrero-Castillo et al. reveals a widespread consequence of mtDNA loss on the mitochondrial proteome, including almost complete destabilisation of mitochondrial ribosome subunits, which is not the case in NME6 knockout cells, making a direct comparison of effects on OXPHOS complexes difficult. We would also like to emphasize that the depletion of OXPHOS subunits occurs in NME6 knockout cells despite normal levels of mtDNA in these cells (Fig 1G and Fig 2E, F). NME6/SLC25A33/SLC25A36 triple knockout cells would most likely phenocopy Rho0 cells since they do lose mtDNA (Fig 1G).

2. Although not strictly necessary in my view for the revision, it would be interesting to know whether the stress response highlighted in Figure S3 A and B is DELE1 dependent.

Our proteomic data indicated that the ISR is induced in NME6 knockout cells and we therefore examined the stress response in more detail. Only a fraction of ISR targets are upregulated in NME6 knockout cells (reviewer Fig. 2) and we also analysed the expression of ISR targets by Nanostring, which did not reveal a clear and complete ISR induction. Furthermore, we did not consistently observe enhanced phosphorylation of eIF2a or increased ATF4 expression in cells lacking NME6 (reviewer Fig. 2). These data raise additional questions about transcriptional responses to mitochondrial dysfunction that will be of interest to examine further. However, since this is not of direct relevance for our data showing a role of NME6 for mitochondrial

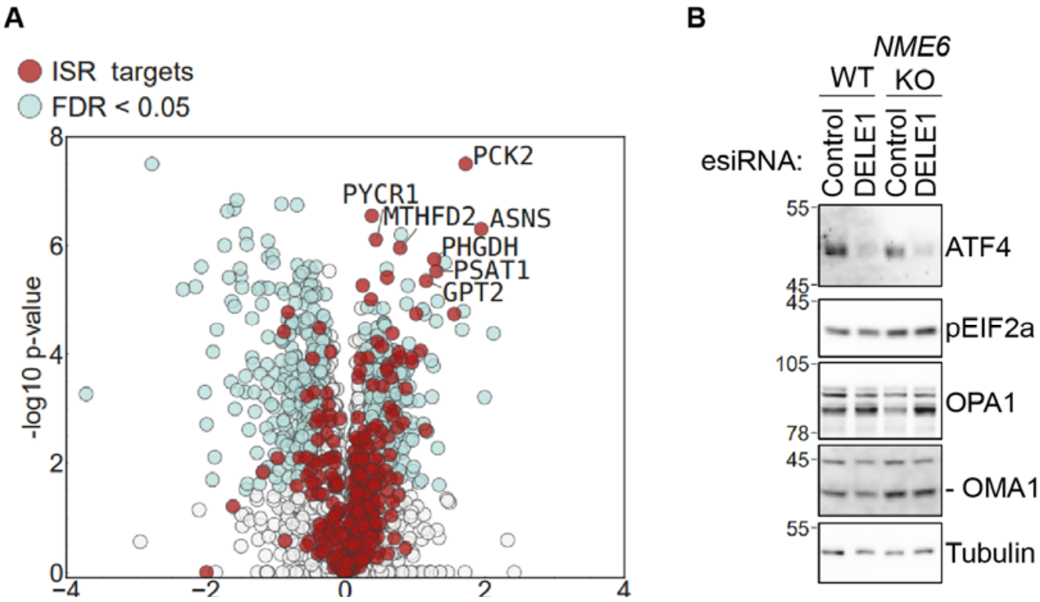

Reviewer Figure 2
A Log2 fold change of protein level in NME6 KO HeLa cells compared to WT HeLa. Known ISR targets are highlighted in red. **B** Immunoblot of WT HeLa and NME6 KO HeLa treated with the indicated esiRNA for 72 h.

transcription, we decided to remove the speculation of NME6's involvement in the ISR.

Additional non-essential suggestions
1. Figure 5 A and 5C. It is not clear what "+" and "-" mean in the graphs. I recommend labeling with "Nucs" like you did in Figure 5D.

We agree with the reviewer and have changed the figure labelling accordingly.

2. Although not strictly necessary in my view for the revision, it would be interesting to know whether the stress response highlighted in Figure S3 A and B is DELE1 dependent.

Please see above.

3. A Related to the observation that NME6 associates with RCC1L and leads to a reduction in RNA granules. Does NME6 itself show a punctate pattern in the mitochondrial matrix and if so does it co-localize with mitochondrial RNA granules? Does the phosphorylation deficient mutant have the same localization pattern? This experiment is also not strictly necessary for the revision in my view but would enhance the manuscript.

To address this question, we imaged endogenous NME6 in combination with mtDNA (with anti-DNA) or nascent mtRNA (with BrU pulse labelling and anti-BrU) with immunofluorescence confocal microscopy (new Fig 4D). We found that NME6 form puncta throughout the mitochondrial network. These puncta did not colocalise with mtDNA nucleoids. While there was more significant overlap between NME6 and mtRNA granules, our data show that NME6 is not exclusively localised to mtRNA granules. To our knowledge, these are the first data to show the localisation pattern of endogenous NME6. We agree with the reviewer that this is a new and intriguing observation and show these data now in the new Fig 4D. In addition, we generated RCC1L knockout cells and found that these cells have reduced levels of NME6 (new Fig EV4C).

We also performed immunofluorescence imaging of WT-NME6-MycFlag and NME6-H137N-MycFlag to address whether the kinase dead mutant has a different localisation pattern. Unfortunately, neither construct formed visible puncta in mitochondria, which we assume is due to the overexpression of these variants. Mutation of endogenous NME6 by CRISPR will likely be required in the future to accurately determine the effect of kinase activity on NME6 localisation.

4. Related to the observation that NME6 associates with RCC1L and leads to a reduction in RNA granules. Does NME6 itself show a punctate pattern in the mitochondrial matrix and if so does it co-localize with mitochondrial RNA granules? Does the phosphorylation deficient mutant have the same localization pattern? This experiment is also not strictly necessary for the revision in my view but would enhance the manuscript.

Please see above.

1st Revision - Editorial Decision **26th May 2023**

Dear Dr. MacVicar,

We have now received re-review reports from all three referees, which I have pasted below. As you will see, you have addressed the concerns of referees 2 and 3 satisfactorily. However, referee 3 has made some suggestions and requests for further clarification. I think it would be best to have a brief Zoom discussion next week about any further revisions which could be made. Would Thursday 1st June work for you?

Thank you again for the opportunity to work with you on this manuscript.

Best wishes,

William

William Teale, PhD
Editor
The EMBO Journal
w.teale@embojournal.org

Instructions for preparing your revised manuscript:

Please check that the title and abstract of the manuscript are brief, yet explicit, even to non-specialists.

When assembling figures, please refer to our figure preparation guideline in order to ensure proper formatting and readability in print as well as on screen:
https://bit.ly/EMBOPressFigurePreparationGuideline
See also figure legend guidelines: https://www.embopress.org/page/journal/14602075/authorguide#figureformat

IMPORTANT: When you send the revision we will require
- a point-by-point response to the referees' comments, with a detailed description of the changes made (as a word file).
- a word file of the manuscript text.
- individual production quality figure files (one file per figure)
- a complete author checklist, which you can download from our author guidelines (https://www.embopress.org/page/journal/14602075/authorguide).
- Expanded View files (replacing Supplementary Information)
Please see out instructions to authors
https://www.embopress.org/page/journal/14602075/authorguide#expandedview

Please remember: Digital image enhancement is acceptable practice, as long as it accurately represents the original data and conforms to community standards. If a figure has been subjected to significant electronic manipulation, this must be noted in the figure legend or in the 'Materials and Methods' section. The editors reserve the right to request original versions of figures and the original images that were used to assemble the figure.

Further information is available in our Guide For Authors: https://www.embopress.org/page/journal/14602075/authorguide

We realize that it is difficult to revise to a specific deadline. In the interest of protecting the conceptual advance provided by the work, we recommend a revision within 3 months (24th Aug 2023). Please discuss the revision progress ahead of this time with the editor if you require more time to complete the revisions.

Referee #1:

On the positive side, the authors have added some additional experiments that firm up one or two conclusions, and have removed a considerable amount of unnecessary text. The new figure 6 summarizing the main conclusions is appreciated, although it lacks one of the conclusions of the abstract (loss of mitochondrial DNA in the absence of the carriers and NME6).

However, the revised abstract is underwhelming and with the best will in the world this reviewer can't escape the feeling that it is not of the level expected of EMBO Journal. The phrase 'shed light on' is particularly disappointing, a phrase that may mean something or nothing. This reviewer began to re-construct the abstract but then stopped, as this is not the task of reviewers.

Also there are some results that are unexplained and others that should be presented and discussed in more depth. Some important statistical comparisons are missing. In Fig 5H there is a comparison of control versus KO, but not KO versus supplements.

Nucleotide supplementation completely rescued OCR but in one of the new experiments it only partially restored cell growth (and several individual results were no different from controls), which is puzzling. It raises the very interesting idea that mitochondrial ribonucleotides support cell growth via a mechanism unrelated to mitochondrial gene expression, or NME6 has another unrecognized function. Therefore, the part on NME6 and cell proliferation feels more like the beginning rather than the end of a project and should be resolved by further experimentation or removed. If it is removed the article contains one principal finding - that NME6 supports (ribo)nucleotide homeostasis in mitochondria. This is novel and interesting but a bit thin for a full article and is perhaps more appropriate to a report format.

The charts of the changes in mitochondrial transcripts in response to nucleotide supplementation should all appear in the main figure 5, as noted previously this rescue effect is important to the main conclusion. These charts are cluttered and difficult to read and the largest decrease caused by adenosine supplementation while interesting is a distraction that compresses the rest of the results. The main point the authors make in the text is that C and U restore mitochondrial transcript levels when NME6 is absent. Therefore, simplify the charts to focus on control vs. KO and KO plus C or U, and show the missing statistical comparisons. Which of the increases in mitochondrial transcripts in the KOs with supplementation is statistically significant, and which not? The full data set, including that the 4 supplements that have no effect in the control cells can be shown in supplemental data.

Other points

Why are ATP6 and ATP8 included as distinct charts in EVF5 when this is a single transcript?

This reviewer was unable to understand the response to the question - How do the authors explain the normal mtDNA level in the face of the marked dCTP shortage in the mitochondria?
The authors replied that: "We speculate that proliferating cells lacking NME6 are still able to maintain sufficient dCTP supply for mtDNA replication via the import of cytosolic dCTP." If dCTP is arriving from the cytosol it will increase the mitochondrial dCTP pool, so the authors are still missing an explanation for the persistent high mitochondrial DNA copy number in the face of the marked dCTP shortage in the mitochondria. The accompanying comment of the authors evades the issue, if the measurements are not in standard units then it is incumbent on the authors to find a means of comparison - there are numerous models of mitochondrial DNA depletion where mitochondrial nucleotide levels could be measured to assess what fold changes are associated with mitochondrial DNA loss. Perhaps extrapolations could be made from other studies. Without further comparisons, the authors should state that although they have these accurate measurements they have no data to say how they relate to mitochondrial DNA replication/copy number.

Delete: "For instance, NME6 is upregulated along with the mitochondrial transcription machinery in certain cases of liver cancer and has been linked to negative prognosis (Fei et al, 2020; Jiang et al, 2019; Wan et al, 2021)." Almost every gene is either up or down regulated in some form of cancer.

Also delete the preceding sentence that is more appropriate to a blog or press release: "It will be exciting to determine whether NME6 is a metabolic vulnerability in the context of enhanced demands on OXPHOS."

Delete inflammation from the abstract as it is misleading to readers, the manuscript contains no data on inflammation.

Referee #2:

The authors have adequately addressed all my concerns.

Referee #3:

The authors have addressed my concerns. Congratulations on this elegant work.

Referee #1:

On the positive side, the authors have added some additional experiments that firm up one or two conclusions, and have removed a considerable amount of unnecessary text. The new figure 6 summarizing the main conclusions is appreciated, although it lacks one of the conclusions of the abstract (loss of mitochondrial DNA in the absence of the carriers and NME6).

However, the revised abstract is underwhelming and with the best will in the world this reviewer can't escape the feeling that it is not of the level expected of EMBO Journal. The phrase 'shed light on' is particularly disappointing, a phrase that may mean something or nothing. This reviewer began to re-construct the abstract but then stopped, as this is not the task of reviewers.

We exchanged *"shed light on"* with *"reveal".*

Also there are some results that are unexplained and others that should be presented and discussed in more depth. Some important statistical comparisons are missing. In Fig 5H there is a comparison of control versus KO, but not KO versus supplements.

Two-way ANOVA is the appropriate statistical test for these data. We have now added further details in the legend to accurately and precisely report the outcome of the test: *"P-values for CTP: two-way ANOVA P-value (supplementation) = <0.0001, P-value (genotype) = <0.0001, P-value (interaction) = 0.3179; P-values for dCTP: two-way ANOVA P-value (supplementation) = <0.0001, P-value (genotype) = <0.0001, P-value (interaction) = 0.0569; P-values for supplementation are shown"*

We have updated all legends corresponding to data where two-way ANOVA were performed.

Nucleotide supplementation completely rescued OCR but in one of the new experiments it only partially restored cell growth (and several individual results were no different from controls), which is puzzling. It raises the very interesting idea that mitochondrial ribonucleotides support cell growth via a mechanism unrelated to mitochondrial gene expression, or NME6 has another unrecognized function. Therefore, the part on NME6 and cell proliferation feels more like the beginning rather than the end of a project and should be resolved by further experimentation or removed. If it is removed the article contains one principal finding - that NME6 supports (ribo)nucleotide homeostasis in mitochondria. This is novel and interesting but a bit thin for a full article and is perhaps more appropriate to a report format.

We point out in the text that neither cytidine nor uridine supplementation *"could fully restore mitochondrial CTP/dCTP in NME6 knockout cells however, which may explain the partial improvement in cell growth upon treatment with cytidine or uridine (Fig EV5F)."* and agree that it will be important to consider other functions for NME6 activity besides OXPHOS in future studies.

The charts of the changes in mitochondrial transcripts in response to nucleotide supplementation should all appear in the main figure 5, as noted previously this rescue effect is important to the main conclusion. These charts are cluttered and difficult to read and the largest decrease caused by adenosine supplementation while interesting is a distraction that compresses the rest of the results.

The main point the authors make in the text is that C and U restore mitochondrial transcript levels when NME6 is absent. Therefore, simplify the charts to focus on control vs. KO and KO plus C or U, and show the missing statistical comparisons. Which of the increases in mitochondrial transcripts in the KOs with supplementation is statistically significant, and which not?
The full data set, including that the 4 supplements that have no effect in the control cells can be shown in supplemental data.

The data presented in Fig5G and EV5E demonstrates the specificity of pyrimidine supplementation for restoration of mitochondrial transcripts in NME6 KO cells and highlights how mitochondrial transcript levels correlate with OCR in Fig5I, which includes the striking effect of adenosine treatment. We also believe it is important and of general interest to report that supplementation with individual nucleosides does not affect mitochondrial transcript levels or OCR in WT cells.

Other points

Why are ATP6 and ATP8 included as distinct charts in EVF5 when this is a single transcript?

Regions corresponding to ATP6 and ATP8 on the bicistronic transcript were detected using separate probes. The log2 FC in transcript level of ATP6 and ATP8 were identical as expected.

This reviewer was unable to understand the response to the question - How do the authors explain the normal mtDNA level in the face of the marked dCTP shortage in the mitochondria?
The authors replied that: "We speculate that proliferating cells lacking NME6 are still able to maintain sufficient dCTP supply for mtDNA replication via the import of cytosolic dCTP." If dCTP is arriving from the cytosol it will increase the mitochondrial dCTP pool, so the authors are still missing an explanation for the persistent high mitochondrial DNA copy number in the face of the marked dCTP shortage in the mitochondria. The accompanying comment of the authors evades the issue, if the measurements are not in standard units then it is incumbent on the authors to find a means of comparison - there are numerous models of mitochondrial DNA depletion where mitochondrial nucleotide levels could be measured to assess what fold changes are associated with mitochondrial DNA loss. Perhaps extrapolations could be made from other studies. Without further comparisons, the authors should state that although they have these accurate measurements they have no data to say how they relate to mitochondrial DNA replication/copy number.

We highlight the fact that mtDNA levels are maintained despite a reduction in mitochondrial dCTP in the discussion: "*NME6 is not required for the maintenance of mtDNA in the cell lines we tested despite a significant reduction in mitochondrial dCTP levels detected in NME6 knockout HeLa cells. Mitochondria contain asymmetric pools of dNTPs and HeLa cells have been reported to have low levels of dCTP in comparison to other dNTP species (Song et al, 2003). Depletion of dCTP is also linked to mtDNA depletion in mitochondrial neurogastrointestinal encephalomyopathy (MNGIE) caused by mutations in thymidine phosphorylase (TYMP) (González-Vioque et al, 2011). We speculate that proliferating cells lacking NME6 are still able to maintain sufficient dCTP supply for mtDNA replication via the import of cytosolic dCTP. NME6 nevertheless becomes essential for the maintenance of mtDNA in cells lacking the mitochondrial pyrimidine transporters, SLC25A33 and SLC25A36. It remains to be seen whether NME6 is required for mtDNA synthesis in quiescent tissues that suppress de novo synthesis of deoxyribonucleotides and depend on mitochondrial nucleotide salvage for the provision of dNTPs for mtDNA synthesis (Ferraro et al., 2005; Mathews & Song, 2007;*

Pontarin et al, 2007). Reduced mitochondrial dCTP may also cause multiple mutations and deletions in the mtDNA of proliferating cells (González-Vioque et al., 2011; Song et al., 2003), which should be explored in future studies alongside the absolute concentrations of mitochondrial dNTPs in the presence and absence of NME6."

Delete: "For instance, NME6 is upregulated along with the mitochondrial transcription machinery in certain cases of liver cancer and has been linked to negative prognosis (Fei et al, 2020; Jiang et al, 2019; Wan et al, 2021)." Almost every gene is either up or down regulated in some form of cancer.

Bioinformatic analysis of TCGA data and published proteomics indicates a robust link between NME6 and liver cancer that we believe is of general interest to report.

Also delete the preceding sentence that is more appropriate to a blog or press release: "It will be exciting to determine whether NME6 is a metabolic vulnerability in the context of enhanced demands on OXPHOS."

Done.

Delete inflammation from the abstract as it is misleading to readers, the manuscript contains no data on inflammation.

Done.

Dear Tom,

I am pleased to inform you that your manuscript has been accepted for publication in the EMBO Journal.

Congratulations on a really insightful study!

Please note that it is EMBO Journal policy for the transcript of the editorial process (containing referee reports and your response letter) to be published as an online supplement to each paper. If you do NOT want this, you will need to inform the Editorial Office via email immediately. More information is available here: https://www.embopress.org/page/journal/14602075/authorguide#transparentprocess

Your manuscript will be processed for publication in the journal by EMBO Press. Manuscripts in the PDF and electronic editions of The EMBO Journal will be copy edited, and you will be provided with page proofs prior to publication. Please note that supplementary information is not included in the proofs.

You will be contacted by Wiley Author Services to complete licensing and payment information. The required 'Page Charges Authorization Form' is available here: https://www.embopress.org/pb-assets/embo-site/tej_apc.pdf - please download and complete the form and return to embopressproduction@wiley.com

EMBO Press participates in many Publish and Read agreements that allow authors to publish Open Access with reduced/no publication charges. Check your eligibility: https://authorservices.wiley.com/author-resources/Journal-Authors/open-access/affiliation-policies-payments/index.html

Should you be planning a Press Release on your article, please get in contact with embojournal@wiley.com as early as possible, in order to coordinate publication and release dates.

If you have any questions, please do not hesitate to call or email the Editorial Office. Thank you for your contribution to The EMBO Journal.

Best wishes,

William

William Teale, PhD
Editor
The EMBO Journal
w.teale@embojournal.org

EMBO Press Author Checklist

Corresponding Author Name: Thomas MacVicar	
Journal Submitted to: EMBO Journal	
Manuscript Number: EMBOJ-2022-113256	

USEFUL LINKS FOR COMPLETING THIS FORM
The EMBO Journal - Author Guidelines
EMBO Reports - Author Guidelines
Molecular Systems Biology - Author Guidelines
EMBO Molecular Medicine - Author Guidelines

Reporting Checklist for Life Science Articles (updated January
This checklist is adapted from Materials Design Analysis Reporting (MDAR) Checklist for Authors. MDAR establishes a minimum set of requirements in transparent reporting in the life sciences (see Statement of Task: 10.31222/osf.io/9sm4x). Please follow the journal's guidelines in preparing your manuscript.
Please note that a copy of this checklist will be published alongside your article.

Abridged guidelines for figures
1. Data
The data shown in figures should satisfy the following conditions:
- the data were obtained and processed according to the field's best practice and are presented to reflect the results of the experiments in an accurate and unbiased manner.
- ideally, figure panels should include only measurements that are directly comparable to each other and obtained with the same assay.
- plots include clearly labeled error bars for independent experiments and sample sizes. Unless justified, error bars should not be shown for technical
- if n<5, the individual data points from each experiment should be plotted. Any statistical test employed should be justified.
- Source Data should be included to report the data underlying figures according to the guidelines set out in the authorship guidelines on Data

2. Captions
Each figure caption should contain the following information, for each panel where they are relevant:
- a specification of the experimental system investigated (eg cell line, species name).
- the assay(s) and method(s) used to carry out the reported observations and measurements.
- an explicit mention of the biological and chemical entity(ies) that are being measured.
- an explicit mention of the biological and chemical entity(ies) that are altered/varied/perturbed in a controlled manner.
- the exact sample size (n) for each experimental group/condition, given as a number, not a range;
- a description of the sample collection allowing the reader to understand whether the samples represent technical or biological replicates (including how many animals, litters, cultures, etc.).
- a statement of how many times the experiment shown was independently replicated in the laboratory.
- definitions of statistical methods and measures:
  - common tests, such as t-test (please specify whether paired vs. unpaired), simple χ2 tests, Wilcoxon and Mann-Whitney tests, can be unambiguously identified by name only, but more complex techniques should be described in the methods section;
  - are tests one-sided or two-sided?
  - are there adjustments for multiple comparisons?
  - exact statistical test results, e.g., P values = x but not P values < x;
  - definition of 'center values' as median or average;
  - definition of error bars as s.d. or s.e.m.

Please complete ALL of the questions below.
Select "Not Applicable" only when the requested information is not relevant for your study.

Materials

Newly Created Materials	Information included in the manuscript?	In which section is the information available? (Reagents and Tools Table, Materials and Methods, Figures, Data Availability Section)
New materials and reagents need to be available; do any restrictions apply?	Not Applicable	

Antibodies	Information included in the manuscript?	In which section is the information available? (Reagents and Tools Table, Materials and Methods, Figures, Data Availability Section)
For **antibodies** provide the following information:<br>- Commercial antibodies: RRID (if possible) or supplier name, catalogue number and or/clone number<br>- Non-commercial: RRID or citation	Yes	Reagents and Tools Table

DNA and RNA sequences	Information included in the manuscript?	In which section is the information available? (Reagents and Tools Table, Materials and Methods, Figures, Data Availability Section)
**Short novel DNA or RNA including primers, probes**: provide the sequences.	Yes	Reagents and Tools Table

Cell materials	Information included in the manuscript?	In which section is the information available? (Reagents and Tools Table, Materials and Methods, Figures, Data Availability Section)
**Cell lines:** Provide species information, strain. Provide accession number in repository **OR** supplier name, catalog number, clone number, and/**OR** RRID.	Yes	Reagents and Tools Table
**Primary cultures:** Provide species, strain, sex of origin, genetic modification status.	Not Applicable	
Report if the cell lines were recently **authenticated** (e.g., by STR profiling) and tested for mycoplasma contamination.	Yes	Material and Methods

Experimental animals	Information included in the manuscript?	In which section is the information available? (Reagents and Tools Table, Materials and Methods, Figures, Data Availability Section)
**Laboratory animals or Model organisms:** Provide species, strain, sex, age, genetic modification status. Provide accession number in repository **OR** supplier name, catalog number, clone number, **OR** RRID.	Not Applicable	
**Animal observed in or captured from the field:** Provide species, sex, and age where possible.	Not Applicable	
Please detail **housing and husbandry conditions**.	Not Applicable	

Plants and microbes	Information included in the manuscript?	In which section is the information available? (Reagents and Tools Table, Materials and Methods, Figures, Data Availability Section)
**Plants:** provide species and strain, ecotype and cultivar where relevant, unique accession number if available, and source (including location for collected wild specimens).	Not Applicable	
**Microbes:** provide species and strain, unique accession number if available, and source.	Not Applicable	

Human research participants	Information included in the manuscript?	In which section is the information available? (Reagents and Tools Table, Materials and Methods, Figures, Data Availability Section)
If collected and within the bounds of privacy constraints report on age, sex and gender or ethnicity for all study participants.	Not Applicable	

Core facilities	Information included in the manuscript?	In which section is the information available? (Reagents and Tools Table, Materials and Methods, Figures, Data Availability Section)
If your work benefited from core facilities, was their service mentioned in the acknowledgments section?	Yes	Acknowledgements

Design

Study protocol	Information included in the manuscript?	In which section is the information available? (Reagents and Tools Table, Materials and Methods, Figures, Data Availability Section)
If study protocol has been **pre-registered, provide DOI in the manuscript**. For clinical trials, provide the trial registration number **OR** cite DOI.	Not Applicable	
Report the **clinical trial registration number** (at ClinicalTrials.gov or equivalent), where applicable.	Not Applicable	

Laboratory protocol	Information included in the manuscript?	In which section is the information available? (Reagents and Tools Table, Materials and Methods, Figures, Data Availability Section)
Provide DOI OR other citation details if **external detailed step-by-step protocols** are available.	Yes	Material and Methods

Experimental study design and statistics	Information included in the manuscript?	In which section is the information available? (Reagents and Tools Table, Materials and Methods, Figures, Data Availability Section)
Include a statement about **sample size** estimate even if no statistical methods were used.	Not Applicable	
Were any steps taken to minimize the effects of subjective bias when allocating animals/samples to treatment (e.g. **randomization procedure**)? If yes, have they been described?	Not Applicable	
Include a statement about **blinding** even if no blinding was done.	Not Applicable	
Describe **inclusion/exclusion criteria** if samples or animals were excluded from the analysis. Were the criteria pre-established?<br><br>If sample or data points were omitted from analysis, report if this was due to attrition or intentional exclusion and provide justification.	Not Applicable	
For every figure, are **statistical tests** justified as appropriate? Do the data meet the assumptions of the tests (e.g., normal distribution)? Describe any methods used to assess it. Is there an estimate of variation within each group of data? Is the variance similar between the groups that are being statistically compared?	Yes	Figure legends

Sample definition and in-laboratory replication	Information included in the manuscript?	In which section is the information available? (Reagents and Tools Table, Materials and Methods, Figures, Data Availability Section)
In the figure legends: state number of times the experiment was **replicated** in laboratory.	Yes	Figure legends
In the figure legends: define whether data describe **technical or biological replicates**.	Yes	Figure legends

Ethics

Ethics	Information included in the manuscript?	In which section is the information available? (Reagents and Tools Table, Materials and Methods, Figures, Data Availability Section)
Studies involving **human participants**: State details of **authority granting ethics approval** (IRB or equivalent committee(s), provide reference number for approval.	Not Applicable	
Studies involving **human participants**: Include a statement confirming that **informed consent** was obtained from all subjects and that the experiments conformed to the principles set out in the WMA Declaration of Helsinki and the Department of Health and Human Services Belmont Report.	Not Applicable	
Studies involving **human participants:** For publication of **patient photos**, include a statement confirming that consent to publish was obtained.	Not Applicable	
Studies involving experimental **animals**: State details of **authority granting ethics approva**l (IRB or equivalent committee(s), provide reference number for approval. Include a statement of compliance with ethical regulations.	Not Applicable	
Studies involving **specimen and field samples:** State if relevant **permits** obtained, provide details of authority approving study; if none were required, explain why.	Not Applicable	

Dual Use Research of Concern (DURC)	Information included in the manuscript?	In which section is the information available? (Reagents and Tools Table, Materials and Methods, Figures, Data Availability Section)
Could your study fall under dual use research restrictions? Please check biosecurity documents and list of **select agents and toxins** (CDC): https://www.selectagents.gov/sat/list.htm	Not Applicable	
If you used a select agent, is the security level of the lab appropriate and reported in the manuscript?	Not Applicable	
If a study is subject to dual use research of concern regulations, is the name of the **authority granting approval and reference number** for the regulatory approval provided in the manuscript?	Not Applicable	

Reporting

The MDAR framework recommends adoption of discipline-specific guidelines, established and endorsed through community initiatives. Journals have their own policy about requiring specific guidelines and recommendations to complement MDAR.

Adherence to community standards	Information included in the manuscript?	In which section is the information available? (Reagents and Tools Table, Materials and Methods, Figures, Data Availability Section)
State if relevant guidelines or checklists (e.g., **ICMJE, MIBBI, ARRIVE, PRISMA**) have been followed or provided.	Yes	
For **tumor marker prognostic studies,** we recommend that you follow the **REMARK** reporting guidelines (see link list at top right). See author guidelines, under 'Reporting Guidelines'. Please confirm you have followed these guidelines.	Not Applicable	
For **phase II and III randomized controlled trials**, please refer to the **CONSORT** flow diagram (see link list at top right) and submit the CONSORT checklist (see link list at top right) with your submission. See author guidelines, under 'Reporting Guidelines'. Please confirm you have submitted this list.	Not Applicable	

Data Availability

Data availability	Information included in the manuscript?	In which section is the information available? (Reagents and Tools Table, Materials and Methods, Figures, Data Availability Section)
Have **primary datasets** been deposited according to the journal's guidelines (see 'Data Deposition' section) and the respective accession numbers provided in the Data Availability Section?	Yes	Data Availability
Were **human clinical and genomic datasets** deposited in a public access-controlled repository in accordance to ethical obligations to the patients and to the applicable consent agreement?	Not Applicable	
Are **computational models** that are central and integral to a study available without restrictions in a machine-readable form? Were the relevant accession numbers or links provided?	Not Applicable	
If publicly available data were reused, provide the respective **data citations in the reference list**.	Yes	References
